# Supplementary material for: Strategies to improve retention in randomised trials: a Cochrane systematic review and meta-analysis
Source: BMJ Open. 2014 Feb 4;4(2):e003821. doi: 10.1136/bmjopen-2013-003821 (PMC3918995; doi:10.1136/bmjopen-2013-003821)
Supplement: Author's manuscript [file bmjopen-2013-003821.draft_revisions.pdf]

Strategies to improve retention in randomised trials: a Cochrane systematic review and meta-analysis

Journal:	*BMJ Open*
Manuscript ID:	bmjopen-2013-003821
Article Type:	Research
Date Submitted by the Author:	15-Aug-2013
Complete List of Authors:	Brueton, Valerie; MRC Clinical Trials Unit, Meta-analysis Group<br>Tierney, Jayne; MRC Clinical Trials Unit, Meta-analysis Group<br>Stenning, Sally; MRC Clinical Trials Unit, Cancer and non infections Group<br>Meredith, Sarah; MRC Clinical Trials Unit,<br>Harding, Seeromanie; MRC, Social and Public Health Sciences Unit<br>Nazareth, Irwin; UCL, Primary Care and Population Health<br>Rait, Greta; UCL, Primary Care and Population Health
**Primary Subject Heading**:	Evidence based practice
Secondary Subject Heading:	Research methods
Keywords:	Retention, Randomised trials, Strategies

SCHOLARONE™ Manuscripts

Title page

Strategies to improve retention in randomised trials: a Cochrane systematic review and meta-analysis[1]

Brueton VC

Research Scientist, Meta-analysis Group
MRC Clinical Trials Unit at UCL
Aviation House
125 Kingsway
London WC2B 6NH
e-mail: vcb@ctu.mrc.ac.uk

Tierney JF

Head of Meta-analysis Group
MRC Clinical Trials Unit at UCL
Aviation House
125 Kingsway
London WC2B 6NH

Stenning S

Senior Statistician
MRC Clinical Trials Unit at UCL
Aviation House
125 Kingsway
London WC2B 6NH

Meredith S

Deputy Director
MRC Clinical Trials Unit at UCL
Aviation House
125 Kingsway
London WC2B 6NH

Harding S

Program leader: Ethnicity and Health
MRC Social & Public Health Sciences Unit
4 Lilybank Gardens
Glasgow G12 8RZ

[1] This article is based on a Cochrane Review to be published in the Cochrane Database of Systematic Reviews (CDSR) 2013, Issue X, DOI:10.1002/14651858.CD00xxxx (see www.thecochranelibrary.com for information)

For peer review only

BMJ Open REVIEW submitted 07.08.2013

Nazareth I

Professor of Primary Care
PRIMENT Clinical Trials Unit
Research Department of Primary care and Population Health
UCL Medical School
Rowland Hill Street
London
NW3 2PF

Rait G

Clinical Senior Lecturer
PRIMENT Clinical Trials Unit
Research Department of Primary care and Population Health
UCL Medical School
Rowland Hill Street
London
NW3 2PF

For peer review only

BMJ Open REVIEW submitted 07.08.2013

Objective

To quantify the effect of strategies to improve retention in randomised trials.

Design

Systematic review and meta-analysis.

Data sources

Sources searched: MEDLINE, EMBASE, PsycINFO, DARE, CENTRAL, CINAHL, C2-SPECTR, ERIC, PreMEDLINE, Cochrane Methodology Register, Current Controlled Trials metaRegister, WHO trials platform, Society for Clinical Trials (SCT) conference proceedings, and a survey of all UK clinical trial research units.

Review methods

Included trials were randomised evaluations of strategies to improve retention embedded within host randomised trials. The primary outcome was retention of trial participants. Data from trials were pooled using the fixed-effect model. Subgroup analyses were used to explore heterogeneity and to determine whether there were any differences in effect by type of strategy.

Results

38 retention trials were identified. Six broad types of strategies were evaluated. Strategies that increased postal questionnaire responses were: adding a monetary incentive (RR 1.18; 95% CI 1.09-1.28) and higher valued incentives (RR 1.12; 95% CI 1.04-1.22). Offering a monetary incentive also increased electronic questionnaire response (RR 1.25; 95% CI 1.14-1.38). The evidence for shorter questionnaires (RR 1.04; 95% CI 1.00-1.08) and questionnaires relevant to the disease/condition (RR 1.07; 95% CI 1.01-1.14) is less clear.

Based on the results of single trials the following strategies appeared effective at increasing questionnaire response: recorded delivery of questionnaires (RR 2.08; 95% CI 1.11-3.87); a "package" of postal communication strategies (RR 1.43; 95% CI 1.22-1.67), and an open trial design (RR 1.37; 95% CI 1.16 -1.63). There is no good evidence that the following strategies impact on trial response/retention: adding a non-monetary incentive (RR=1.00; 95% CI 0.98-1.02); offering a non-monetary incentive (RR=0.99; 95% CI 0.95-1.03); "enhanced" letters (RR=1.01; 95% CI 0.97-1.05); monetary incentives compared to offering prize draw entry (RR=1.04; 95% CI 0.91- 1.19); priority postal delivery (RR=1.02; 95% CI 0.95 - 1.09); behavioural motivational strategies (RR= 1.08; 95% CI 0.93-1.24); additional reminders to participants (RR=1.03; 95% CI 0.99-1.06); and questionnaire question order (RR=1.00, 0.97-1.02).

For peer review only - http://bmjopen.bmj.com/site/about/guidelines.xhtml

For peer review only

Also based on single trials, these strategies do not appear effective: a telephone survey compared to a monetary incentive plus questionnaire (RR=1.08; 95% CI 0.94-1.24); offering a charity donation (RR =1.02, 95% CI; 0.78-1.32); sending sites reminders (RR= 0.96; 95% CI 0.83-1.11); sending questionnaires early (RR=1.10; 95% CI 0.96-1.26); longer and clearer questionnaires (RR= 1.01, 0.95-1.07) and case management (RR=1.00; 95% CI 0.97-1.04).

Conclusion

Most trials evaluated questionnaire response rather than ways to improve participants return to site for follow-up. Monetary incentives and offers of monetary incentives increase postal and electronic questionnaire response. Some strategies need further evaluation. Application of these results would depend on trial context and follow-up procedures.

For peer review only

For peer review only - http://bmjopen.bmj.com/site/about/guidelines.xhtml

Introduction

Loss of participants during study follow-up can introduce bias and reduce power affecting the generalisability, validity, and reliability of results[1,2] . If losses are fewer than 5% they may lead to minimum bias, while 20% loss can threaten trial validity[2]. While missing data from losses to follow-up can be dealt with statistically, the risk of bias can remain[3].

Trialists adopt various strategies to try to improve retention and generate maximum data return or compliance to follow-up procedures. These strategies are designed to motivate and keep participants or site clinicians engaged in a trial, but many are untested[4,5]. A systematic review of strategies to retain participants cohort studies suggests that providing incentives can improve retention[6]. Edwards systematic review on methods to increase response rates to postal and electronic questionnaires across a range of study types found that including monetary incentives, keeping the questionnaire short and contacting people before questionnaires were sent were ways to increase response rates[7]. However, heterogeneity of effects was an issue and it is unclear which strategies are applicable to randomised trials. Moreover, reasons for loss to follow-up in cohort studies and surveys may differ from randomised trials. In trials, participants may be randomised to a study arm that is not their preferred choice and so strategies that improve retention in other study types cannot necessarily be extrapolated to randomised trials.

As loss to follow-up can compromise the validity of findings from randomised trials, delay results and potentially increase trial costs, we conducted a systematic review to assess the effect of strategies to improve retention in randomised trials.

Methods

The methods were pre-specified in the Cochrane review protocol[8].

Trials included

We included randomised trials that compared strategies to increase participant retention embedded in "host" randomised trials across disease areas and settings. These strategies should have been designed for use after participants were recruited and randomised. Retention trials embedded in cohort studies and surveys were excluded.

Identification of retention trials

We searched MEDLINE, EMBASE, PsycINFO, DARE, Cochrane CENTRAL and CINAHL to May 2012 using randomised controlled trial filters, where possible and free text terms for

For peer review only - http://bmjopen.bmj.com/site/about/guidelines.xhtml

retention. C2-SPECTR and ERIC were only searched to May 2009 because of difficulties encountered with database and search platform changes. PreMedline was searched to May 2009 but not subsequently because the free text records ultimately appear in MEDLINE. For search updates we also included the Cochrane Methodology Register, Current Controlled Trials metaRegister of Controlled Trials and WHO trials registry. Reference lists of relevant publications, reviews, included studies and abstracts of Society for Clinical Trials meetings from 1980-2012 were also reviewed. No language restrictions were applied. All UK clinical trial units were surveyed to identify further eligible trials and the review was advertised at the Society for Clinical Trials Meeting in 2010.

Trial selection

Two reviewers (VB, GR) independently screened potentially eligible trials with disagreements resolved by a third author (SS). Information was sought from investigators to clarify eligibility where this was unclear.

Data extraction

Data were extracted for each retention and host trial by one author (VB) and checked by another (JT). For retention trials, data were extracted on start time in relation to the host trial, aim, primary outcome, follow-up type, strategy to improve retention and comparator/s, including the frequency and time the strategy was administered, and numbers randomised, included and retained at the primary analysis. Data on sequence generation, allocation concealment, blinding and outcome reporting were extracted for each retention trial to assess risk of bias[9]. Data extracted for each host trial were: aim, comparators, primary outcome, disease area and setting. In addition, information on the sequence generation and allocation concealment was extracted to confirm that host trials were randomised. Missing or ambiguous data were queried or obtained through contact with trial authors.

Statistical analysis

Retention was the primary outcome. Where retention trials specified the primary outcome as the retention rate at a particular time point, this was used in the analysis. Where trials reported retention at multiple time points, without specifying which one was the primary outcome, we used the earliest time point in the analysis. Where trials reported time to retention, without specifying the primary time point, we used the final time point in the analysis, taking account of any censoring if data were available.

Retention trials with insufficient data could not be included in meta-analyses and were described qualitatively. Otherwise, risk ratios and their 95% confidence intervals for retention were used to determine the effect of strategies on this outcome. The participant was the unit

For peer review only

of analysis. Where clustering was ignored in the analysis of cluster randomised trials we inflated the standard errors using the intra-class correlation coefficients from appropriate external sources[10,11 12].

For factorial trials [13,14] that investigated different categories of strategies to improve retention, we included all trial comparisons in the relevant analyses and labelled these accordingly. For one factorial trial[15], where the data were not available to do this, only the broad trial comparisons (main effects) were included in the analyses. Where there were multiple comparisons in a single trial[16] within the same category of strategy, to avoid double counting, the intervention arms were combined and compared with the control arm. Similarly, for three-armed trials[17,18] that compared two similar intervention arms with one control arm, the intervention arms were combined and compared with the control arm. For these trials, we also compared each intervention arm with the control arm, as separate trial comparisons, in exploratory analyses. Note that these approaches resulted in more trial comparisons than trials.

Heterogeneity was examined the chi^2 test, at 0.10 level of significance, and the I^2 statistic[19], and explored through subgroup analyses. If there was no substantial heterogeneity, risk ratios were pooled using the fixed effect model, but if heterogeneity was detected and was not explained by subgroup or sensitivity analyses, we did not pool results. To assess the robustness of the results, sensitivity analyses were conducted that excluded quasi-randomised trials.

The diversity of trials and interventions identified meant that not all of our pre-specified subgroup analyses were appropriate or possible. Therefore, different types of strategies were analysed separately and new subgroups were defined within these prior to analysis. These new analyses are listed in tables 1- 4.

Absolute benefits of effective retention strategies were based on applying meta-analysis risk ratios to representative control arm retention rates[20].

Results

We identified 38 eligible randomised retention trials from 24,304 records (Fig 4). Twenty-eight of these were published in full[13-18,21-38], two in the grey literature[14,34] and eight are unpublished (*unpublished trials by Edwards, Svobodva, Letley, Maclennan, Land, Bailey 1, Bailey 2 Marson).* Four retention trial publications contained two trials each[18,32,33,35].

For peer review only - http://bmjopen.bmj.com/site/about/guidelines.xhtml

BMJ Open REVIEW submitted 07.08.2013

Participants and settings

Eligible retention trials were from different geographical areas and clinical settings. Clinical areas ranged from exercise and alcohol dependency to treatment and screening for cancer (Tables 1- 4)[12].

Outcomes for strategies to improve retention were measured by: return of postal or electronic questionnaires[13-15,18,21,22,24,25,27,29-34,36-41] (*unpublished trials by Edwards, Svobodva, Letley, Maclennan, Land, Bailey 1, Bailey 2 Marson)* or biomedical data [17] (*Bailey unpublished*) a combination of postal, telephone, and email follow-up[35] or face to face follow-up/retention[16,42].

Design of included retention trials

One retention trial was cluster randomised (*Land unpublished*), four were factorial trials[13-16] and there was one three-armed[17] and three four-armed trials[18,32]. Five trials were quasi randomised[16,29,33,42], allocating participants by either their identification numbers[29,42], day of clinic visit[16] or by random selection of half the sample for the intervention and half for the control group[33]. All strategies targeted individual trial participants except one which targeted sites (*Land unpublished*).

Twenty nine retention trials commenced during follow-up of the host trial[13,15,16,18,21,22,24-27,29-36,38,42,43] (*Edwards, Land, Maclennan, Bailey, Svoboda, unpublished*). One trial followed children of mothers who participated in the MRC ORACLE trial[39]. Two trials followed up participants in smoking cessation trials after the host trial finished[17,40]. Another retention trial randomised participants before the host trial commenced[23]. Four trials commenced during the pilot phase of the host trial[18,32,37] (*Letley unpublished*). For one trial it is unclear when the retention trial commenced in relation to the host trial[14].

Incentive strategies

There were 14 retention trials of incentives and 19 trial comparisons. Thirteen trials investigating incentive strategies targeted questionnaire response, with only one targeting participant retention[16]. Incentive strategies aimed at improving questionnaire response were: vouchers[18,29,39], cash[25], a charity donation[18], entry into a prize draw[14,18,30], cheques[14,17] offers of study results[24,40] and a certificate of appreciation[15,16]. Incentive strategies aimed at participant retention were: lapel pins and a certificate of appreciation[16]. UK incentives ranged in value from £5-£20[18,29,39] (*Bailey unpublished*) and from $2-$10 for US based

For peer review only - http://bmjopen.bmj.com/site/about/guidelines.xhtml

trials, and were provided as either cash or voucher. Offers of entry into prize draws ranged from £25- £250 for UK[18,30] and $US50 for US based trials[14] (Table 1). One trial evaluated giving a monetary incentive with a promise of a further incentive for return of trial data (*Bailey 2 unpublished*).

Communication strategies

There were 14 retention trials of communication strategies and 20 trial comparisons. Most communication strategies targeted questionnaire response, with only one targeted at the return of biomedical test kits[35]. Strategies evaluated were: enhanced letters[15] (*Marson unpublished*) use of additional telephone reminders[35] (*Maclennan unpublished*); a calendar including reminders of when to return a questionnaire[34]; text and/or email reminders[21,31,35] and reminders to sites of upcoming assessments versus no additional reminder (*Land unpublished*). One trial used a package of postal communication strategies called the Total Design Method (TDM)[37] and another used recorded delivery of questionnaires[38] (Table 2). Five trials evaluated both communication and incentive strategies[13-15,25,35] (Tables 1 and 2). The incentives were: certificates of appreciation for study involvement[15], study branded pens[13], a US$2 coin[14] and a US$5 bill [25] or fridge magnets[35]. The communication strategies were: 1st or 2nd class outward post[13-15] stamped and business reply envelopes[13], letters signed by different study personnel[15], letters posted at different times[15], telephone survey[25] and text messages[35].

New questionnaire formats

The effect of a change in questionnaire format on response to questionnaires was evaluated in eight trials. The 10 comparison formats evaluated were (Table 3): questionnaire length[27,32,36] (*Edwards unpublished Svoboda unpublished*) order of questions (Letley *unpublished*)[33] and relevance of questionnaires in the context of research in alcohol dependence [32].

Behavioural strategies

There were two retention trials of motivational behavioural strategies, one in an exercise trial[26] and another in a parenting trial[23] (Table 4). One retention trial was run prior to the host trial[23], where only participants who completed the orientation/retention trial were included in the subsequent parenting trial.

Case management

Case management defined as outreach, service planning linkage, monitoring, and advocacy, was compared within usual follow-up in a cancer screening trial[28](Table 4).

Methodology strategies

For peer review only

One trial included an open trial versus blind trial design to evaluate the impact on questionnaire response[22] (Table 4).

Trials not included in the meta-analyses

Two included trials could not be included in the meta-analysis[30] (*Letley unpublished*). For one, the host trial participants included randomised and non-randomised participants[30] and the author confirmed that participants in the retention trial were from both cohorts and these data could not be separated. For the other, retention trial (*Letley unpublished*) outcome data were not available.

Risk of bias in included trials

Twenty four trials describe adequate sequence generation[15,16,18,22-24,26,30-32,34,35,37,39,40] (*unpublished trials Bailey2 Bailey1 Letley, Land, Maclennan, Marson*). There was insufficient information about the sequence generation for ten trials, but they were all described as randomised[13,14,17,21,25,27,36,38] (*Edwards, Svoboda unpublished*). Five trials used quasi randomisation[16,28,29,33]. Fifteen trials reported both adequate sequence generation and allocation concealment[18,22,24,26,31,32,34,39,40] (*Letley, Maclennan, Bailey [1,2,] unpublished*).

Blinding of participants to the intervention was not possible for incentive strategies offers of incentives, behavioural or case management strategies, and different types of communication and questionnaire format strategies and for one trial that evaluated the effect of a blind versus open design on retention this was not applicable[22]. For some trials, participants were aware of the intervention but unaware of the evaluation[14,16,23,30,33,39] (*Maclennan, Marson unpublished*). For another trial[26] exercise sessions were not separated according to the behavioural intervention i.e. walking and swimming, and potential contamination between groups could have led to bias. For other trials, blinding of participants or trial personnel to the outcome or intervention was not reported. The primary outcome measure for this review was retention, and this was well reported. Authors were contacted for clarification of any exclusions after randomisation if this was unclear from retention trial reports. Although retention trial protocols were not available for included trials, the published and unpublished reports included reported all expected outcomes for retention.

The effects of strategies

1. Incentive Strategies

There were 14 retention trials of incentives, 19 trial comparisons with 16,253 comparisons. Across incentive subgroups there was considerable heterogeneity (p<0.00001) Figure 1a. So

For peer review only

we did not pool the results for incentives. Three trials (3166 participants) that evaluated the effect of giving monetary incentives to participants showed that the addition of monetary incentives is more effective than no incentive at increasing response to postal questionnaires (RR=1.18; 95% CI 1.09-1.28; p<0.0001, heterogeneity p=0.21 Figure 1a). A sensitivity analysis excluding the quasi randomised trial by Gates shows a similar effect (RR=1.31; 95% CI 1.11-1.55; p=0.002)[29]. Also, based on two web based trials (3613 participants, Figure 1a), an offer of a monetary incentive promotes greater return of electronic questionnaires than no offer (RR=1.25; 95% CI 1.14-1.38, p<0.00001, heterogeneity p=0.14). However, a single trial comparison suggests that an offer of a monetary donation to charity does not increase response to electronic questionnaires (RR =1.02, 95% CI; 0.78-1.32; p=0.90 Figure 1a)

Based on three trials (6322 participants) there is no clear evidence that the addition of non-monetary incentives improved questionnaire response (RR=1.00; 95% CI 0.98-1.02; p=0.91) but there is some heterogeneity (p=0.02 Figure 1a). A sensitivity analysis excluding the quasi randomised trial by Bowen showed a similar effect (RR=1.00; 95% CI 0.93-1.08; p=0.99, heterogeneity p=0.01) [16]. Two trials (1,138 participants) evaluating offers of non-monetary incentives suggest that an offer of a non-monetary incentive is neither more nor less effective than no offer (RR=0.99; 95% CI 0.95-1.03; p=0.60; heterogeneity p=0.52) at improving questionnaire response Figure 1a.

In exploratory analyses, the different incentive arms that were combined for the main analysis do not appear to show differential effects (Figure 5).

Two trials (902 participants) show that higher value incentives are better at increasing response to postal questionnaires than lower value incentives (RR 1.12; 95% CI 1.04-1.22; p =0.005; heterogeneity p=0.39) irrespective of how they are given (Figure 1b).

Two trial comparisons (297 participants) provide no clear evidence that giving a monetary incentive is better than an offer of entry into a prize draw for improving response to postal questionnaires (RR=1.04; 95% CI 0.91- 1.19; p=0.56, heterogeneity p=0.18, Figure 1c).

One trial could not be included in the analysis[30], but showed a higher response in the group offered entry into a prize draw (70.5%) compared with the group not offered entry into the draw (65.8%).

For peer review only - http://bmjopen.bmj.com/site/about/guidelines.xhtml

BMJ Open REVIEW submitted 07.08.2013

2. Communication strategies

There were 14 trials of communication strategies and 20 comparisons with 9,822 participants.

Results from two trials (2479 participants) show that an enhanced letter is neither more nor less effective than a standard letter for increasing response to postal questionnaires (RR=1.01; 95% CI 0.97-1.05; p=0.70; heterogeneity p=0.80, Figure 2a) . Although based on a single trial (226 participants), the TDM package seems much more effective than a customary postal communication method at increasing questionnaire return (RR=1.43, 95% CI 1.22-1.67; p<0.0001 Figure 2b). Based on the relevant arms of three trials (1888 participants), there is no clear evidence that priority post is either more or less effective than regular post at increasing trial questionnaire return (RR=1.02; 95% CI 0.95-1.09; p=0.55; heterogeneity p=0.53 Figure 2c).

Six trials (3401 participants) evaluated the effect of different types of reminders to participants on questionnaire response. There is no clear evidence that a reminder is either more or less effective than no reminder (RR=1.03; 95% CI 0.99-1.06; p=0.13; heterogeneity p=0.73) at improving trial questionnaire response (Figure 2d). One trial (700 participants) showed no clear evidence that a telephone survey is either more or less effective than a monetary incentive and a questionnaire for improving questionnaire response (RR=1.08; 95% CI 0.94-1.24; p=0.27, Fig 2e). Based on one cluster randomised trial (272 participants), a monthly reminder to sites of upcoming assessment was neither more nor less effective than the usual follow-up (RR=0.96; 95% CI 0.83-1.11; p=0.57). However, one small trial (192 participants) suggested that recorded delivery is more effective than a telephone reminder (RR= 2.08; 95% CI 1.11-3.87; p=0.02). Based on one other trial (664 participants), there is no clear evidence that sending questionnaires early increased or decreased response (RR=1.10; 95% CI 0.96-1.26; p=0.19).

3. New questionnaire strategies

Eight trials with ten comparisons (21,505 participants) evaluated the effect of a new questionnaire format on questionnaire response. Although there is modest heterogeneity between the questionnaire subgroups p=0.11 (Figure 3), it did not seem reasonable to pool the results based on such different interventions.

Five trials (7277 participants) compared the effect of short questionnaires versus long on postal questionnaire response. There is only a suggestion that short questionnaires may be

For peer review only

better (RR=1.04; 95% CI 1.00-1.08; p=0.07, heterogeneity p=0.14, Figure 3). Based on one trial (900 participants), there is no clear evidence that long and clear questionnaires are more or less effective than shorter condensed questionnaires for increasing questionnaire response (RR= 1.01, 0.95-1.07; p=0.86, Figure 3). Two quasi randomised trials (9435 participants) also show no good evidence that placing disease/condition questions before generic questions is either more or less effective than vice versa at increasing questionnaire response (RR=1.00, 0.97-1.02; p=0.75, heterogeneity (p=0.44), Figure 3). One trial by Letley (*unpublished*) not included in this analysis, provided no estimate of effect.

In the context of research on reducing alcohol consumption there is also evidence that more relevant questionnaires i.e. those relating to alcohol use, increase response rates (RR 1.07; 95% CI 1.01-1.14; p= 0.03, Figure 3).

4. Behavioural / motivational strategies

Two community based trials (273 participants) show no clear evidence that the behavioural / motivational strategies used are either more or less effective than standard information for retaining participants (RR= 1.08; 95% CI 0.93-1.24; p=0.31 heterogeneity p=0.93)

5. Case management strategies

One trial (703 participants) evaluated the effect of intensive case management procedures on retention. There is no evidence that intensive case management is either more or less effective than usual follow-up in the population examined (RR=1.00; 95% CI 0.97-1.04; p=0.99)

6. Methodology strategies

One fracture prevention trial (538 participants) evaluated the effect of participants knowing their treatment allocation (open trial) compared to participants blind/unaware of their allocation on questionnaire response. The open design led to higher response rates (RR=1.37; 95% CI 1.16 -1.63; p=0.0003).

Absolute benefits of strategies to improve retention

The absolute benefits of effective strategies on typical questionnaire response are illustrated in Table 5. Based on a 40% baseline response rate for postal questionnaires, the addition of a monetary incentive is estimated to increase response by 92 questionnaires per 1000 sent (95% CI 50-131). With a baseline response rate of 30%, as seen in the included online trial, the

For peer review only - http://bmjopen.bmj.com/site/about/guidelines.xhtml

addition of an offer of a monetary incentive is estimated to increase response by 140 questionnaires per 1000 (95% CI 86-193).

Discussion

Thirty-eight randomised retention trials were included in this review, evaluating six broad types of strategies to increase questionnaire response and retention in randomised trials. Trials were conducted across a spectrum of disease areas, countries, health care, and community settings. Strategies with the clearest impact on questionnaire response were: addition of monetary incentives compared to no incentive for return of postal questionnaires, addition of an offer of a monetary incentive when compared to none for return of electronic questionnaires, and an offer of £20 vouchers when compared to £10 for return of postal questionnaires and biomedical test kits. The evidence was less clear about the effect of shorter questionnaires rather than longer questionnaires and for questionnaires of greater relevance to the questions being studied. Recorded delivery of questionnaires, the Total Design Method a "package" of postal communication strategies with reminder letters and an open trial design appear more effective than standard procedures. These strategies were tested in single trials and may need further evaluation. The addition of a non-monetary incentive or an offer of a non-monetary incentive compared to no incentive did not increase or decrease trial questionnaire response. "Enhanced" letters, letters delivered by priority post or additional reminders were also no more effective than standard communication. Altering questionnaire structure does not seem to increase response. No strategy had a clear impact on increasing the number of participants returning to sites for follow-up.

Strengths and weaknesses

This is the most comprehensive review of strategies specifically designed to improve retention in randomised trials, including many unpublished trials and data. Although our searches were extensive, some less well reported, on-going, or unpublished trials, or trials conducted outside the UK might have been missed.

Most trials used appropriate methods for randomisation or at least stated that they were randomised. For trials that did not describe their methods well or provide further information, there remains a potential risk of selection bias. Sensitivity analyses excluding quasi-randomised trials did not affect the results. In this context, where motivating participants to

For peer review only - http://bmjopen.bmj.com/site/about/guidelines.xhtml
For peer review only

provide data or attend clinics is often the target of the interventions and so appropriately influences the outcome, lack of blinding is less of a concern. Retention is the outcome and was obtained for all but two trials so similarly, attrition and selective outcome reporting bias are probably unimportant. Although the retention trials were fairly well conducted, this could be improved, and they were often poorly reported. This may be because they were designed when loss to follow-up became a problem in a trial, rather than pre planned prior to host trial commencement.

All included studies were conducted in higher income countries. Therefore, the effective strategies may not be socially, culturally or economically appropriate to trials conducted in low resource settings. The diversity of strategies and the low number of trials meant that we could not examine the impact of, for example, trial setting and disease area as planned. Moreover, most of the evidence relates to increasing questionnaire response rather than participant retention in follow-up.

Edwards extensive review of methods to increase response to postal and electronic questionnaires found that monetary incentives and recorded delivery of questionnaires improved response[7]. However, unlike our review they also found that non-monetary incentives, shorter questionnaires, use of handwritten addresses, stamped return envelopes (as opposed to franked return envelopes) and, first class outward mailing were effective. We did however find that a "package" including an enhanced letter with several reminders was effective. The trials included in the Edwards review were embedded in surveys, cohort studies and trials and there was substantial heterogeneity in the results, which was not a particular problem in this review [7]. Moreover, we included seven unpublished trials and 18 other trials not included by Edwards[12].

Nakash's small systematic review of ways to increase response to postal questionnaires in health care was not exclusive to randomised trials[44]. They found reminder letters, telephone contact, and short questionnaires increased response to postal questionnaires. There was no evidence that incentives were effective. A systematic review of methods to increase retention in population based cohort studies had no meta-analysis, but suggested that incentives were associated with increased retention[6].

For peer review only

Prior to our review, it was not clear which if any of these strategies could be extrapolated to randomised trials. We also identified additional strategies that may improve trial questionnaire response or retention for example, methodological strategies.

Implications

Although giving monetary incentives up front seems effective, offering and giving these after receipt of data could be a cost effective strategy, because those not returning questionnaires would not receive an incentive. The addition of non-monetary incentives for example, lapel pins and certificates of appreciation, or offers of these did not increase response or retention, perhaps because these items are not valued by participants. Offers of monetary incentives were also an effective strategy in the context of an online electronic questionnaire, thus it would be beneficial for trialists to know which is more effective: an offer of a monetary incentive or an upfront monetary incentive.

Priority post, enhanced letters (e.g. signed by the principal investigator) and different types of additional reminders are used by trialists in current research practice, but were not found to be effective. The former may not be considered important and too many reminders, over and above standard procedures, could be counterproductive.

Although appearing very effective, the total design method for postal questionnaires could be labour intensive to implement, expensive, and may no longer be applicable to some participant groups e.g. young people used to other modes of communication, or in trials using email, text or online data collection. Recorded delivery could be useful to ensure trial follow-up supplies reach their intended destination, but careful planning to avoid inconvenience for the participant might be necessary. Open trials to increase questionnaire response can only be used where blinding is not required. This could be counterproductive, however, as unblinded trials can cause biased outcome assessment or loss to follow-up if a participant or clinician has a treatment preference.

Questionnaire length and relevance may need further evaluation as there is only a suggestion that these are effective in the context of randomised trials. Also, telephone follow-up compared with a monetary incentive sent with a questionnaire needs further evaluation possibly with a cost benefit analysis as both could be expensive in time and human resources.

For peer review only

For peer review only - http://bmjopen.bmj.com/site/about/guidelines.xhtml

Evaluations of strategies that encourage participants to return to sites for follow-up visits and monitoring are particularly needed because many trials collect outcome data in this way.

Conclusions

Trialists should consider using monetary incentives and offers of monetary incentives to increase postal and electronic questionnaire response, depending on trial setting, population, disease area, budget, and usual follow-up procedures.

Future evaluations of retention strategies in randomised trials should be carefully planned and adequately powered, and the retention strategies and measures of retention clearly defined. More research on ways to increase return of participants to sites for follow-up, and on ways to retain sites in cluster and individual randomised trials are also needed.

Acknowledgements

We would like to thank the following: all authors of included published trials for providing extra unreported data; principal investigators for data on trials in progress or completed and unpublished (Julia Bailey UCL for data for Bailey 1 and Bailey 2; Graeme MacLennan for data for MacLennan; Stephanie Land data for Land) and the coordinators of UK Clinical Trials Units who responded to our survey with information about on going and/or unpublished completed trials. We also thank Cara Booker, SPHRU for search strategy information; Angela Young, Librarian UCL for assistance with searching databases and Ian White MRC Bio Statistics Unit Cambridge for helpful comments on the analysis of multi arm trials. We also acknowledge Shaun Treweek, Mike Clarke, Andy Oxman, Karen Robinson and Anne Eisinga for comments on the review protocol; and Phil Edwards, Elie Akl, Lisa Maguire, Jean Suvan, Shaun Treweek, Mike Clarke and Karen Robinson for comments on the review. This project was funded by the MRC Population Health Sciences Research Network (Grant Number PHSRN 30).

Statement of funding and competing interests

All authors have completed the ICMJE uniform disclosure form and declare no competing interests. This project was funded by the Medical Research Council Population Health Sciences Research Network grant number PHSRN 30.

For peer review only

For peer review only - http://bmjopen.bmj.com/site/about/guidelines.xhtml

Article summary

Article focus

Loss to follow-up in randomised trials can cause bias and loss of power.

Many strategies are routinely used in an attempt to improve retention in randomised trials.

The effect of strategies used to improve retention in randomised trials has not been formally evaluated until now. This systematic review identifies strategies that have been evaluated in randomised trials and quantifies the effect of these strategies to improve retention in randomised trials.

Key messages

This is the first systematic review to evaluate the effect of strategies to improve retention in randomised trials.

Effective strategies for increasing postal questionnaire response were: monetary incentives, offers of monetary incentives, and higher valued incentives.

Strategies that encourage participant to return to sites for follow-up visits and monitoring are particularly needed. Other strategies need further evaluation.

Such evaluations need to be rigorous and adequately reported

Strengths and limitations of this study

This is the most comprehensive review of strategies specifically designed to improve retention in randomised trials, including many unpublished trials and data.

Although our searches were extensive, some less well reported, on-going, or unpublished trials, or trials conducted outside the UK might have been missed.

For peer review only

For peer review only

Reference List

(1) Fewtrell MS, Kennedy K, Singhal A, Martin RM, Ness A, Hadders-Algra M et al. How much loss to follow-up is acceptable in long-term randomised trials and prospective studies? *Arch Dis Child* 2008; 93(6):458-461.

(2) Schulz KF, Grimes DA. Sample size slippages in randomised trials: exclusions and the lost and wayward. *The Lancet* 2002; 359(9308):781-785.

(3) Hollis S, Campbell F. What is meant by intention to treat analysis? Survey of published randomised controlled trials. *BMJ* 1999; 319:670-674.

(4) Robinson KA, Dennison CR, Wayman DM, Pronovost PJ, Needham DM. Systematic review identifies number of strategies important for retaining study participants. *J Clin Epidemiol* 2007; 60(8):757.

(5) Davis L, Broome M, Cox R. Maximizing Retention in Community-based Clinical Trials. *Journal of Nursing Scholarship* 2002; 34(1):47-53.

(6) Booker C, Harding S, Benzeval M. A systematic review of the effect of retention methods in population-based cohort studies. *BMC Public Health* 2011; 11(1):249.

(7) Edwards PJ, Roberts IG, Clarke MJ, DiGuiseppi C, Wentz R, Kwan I et al. Methods to increase response rates to postal and electronic questionnaires. *Cochrane Database of Systematic Reviews 2009, Issue 3 Art No : MR000008* 2009;(3).

(8) Brueton VC, Rait G, Tierney J, Meredith S, Darbyshire J, Harding S et al. Strategies to reduce attrition in randomised trials. *Cochrane Database of Systematic Reviews Art No :MR000032 DOI: 10 1002/14651858 MR000032* 2011;(2).

For peer review only - http://bmjopen.bmj.com/site/about/guidelines.xhtml

(9) Higgins J, Altman D. Assessing risk of bias in included studies. In: Higgins J, Sally Green, editors. Cochrane Hanbook for Systematic Reviews of Interventions. John Wiley; 2008. 187-242.

(10) Higgins J, Deeks.J, Altman D. Special topics in statistics. In: Julian PT Higgins, Sally Green, editors. Cochrane Handbook for Systematic Reviews of Intervenions. John Wiley; 2008. 482-529.

(11) University of Aberdeen. Aberdeen ICCs. 2013.

Ref Type: Online Source

(12) Brueton VC, Tierney J, Stenning S, Nazareth I, Meredith S, Harding S et al. Strategies to improve retention in randomised trials . *Cochrane Methodology Group* 2013; in press.

(13) Sharp L, Cochran C, Cotton SC, Gray NM, Gallagher ME. Enclosing a pen with a postal questionnaire can significantly increase the response rate. *J Clin Epidemiol* 2006; 59(7):747-754.

(14) Kenton L, Dennis CL, Weston J, and Kiss A. Abstracts from the 28th Meeting of the Society of Clinical Trials, Montreal, May 20–23, 2007:The effect of incentives and high priority mailing on postal questionnaire response rates: A Mini-RCT. <*[11] Journal*> 1-8-2007; 4(4):371-455.

(15) Renfroe EG, Heywood G, Foreman L, Schron E, Powell J, Baessler C et al. The end-of-study patient survey: methods influencing response rate in the AVID Trial. *Control Clin Trials* 2002; 23(5):521-533.

(16) Bowen D, Thornquist M, Goodman G, Omenn GS, Anderson K, Barnett M et al. Effects of Incentive Items on Participation in a Randomized Chemoprevention Trial. *J Health Psychol* 2000; 5(1):109-115.

(17) Bauer JE, Rezaishiraz H, Head K, Cowell J, Bepler G, Aiken M et al. Obtaining DNA from a geographically dispersed cohort of current and former smokers: Use of mail-based mouthwash collection and monetary incentives. *Nicotine & Tobacco Research* 2004; 6(3):439-446.

(18) Khadjesari Z, Murray E, Kalaitzaki E, White I, Mc Cambridge J, Thompson S et al. Impact and costs of incentives to reduce attrition in online trials:Two randomised controlled trials. *Journal of Medical Internet Research* 2011; 13(1):e26.

(19) Higgins JPT, Thompson SG, Deeks JJ, Altman DG. Measuring inconsistency in meta-analyses. *BMJ* 2003; 327(7414):557-560.

(20) Schunemann H, Oxman AD, Visr G, Higgins J, Deeks D, Glasziou P et al. Interpreting results and drawing conclusions. In: Higgens J, Green S, editors. Cochrane Handbook

For peer review only

for Systematic Reviews of Interventions. Chichester West Sussex: John Wiley and Sons Ltd; 2008. 359-387.

(21) Ashby R, Turner G, Cross B, Mitchell N, Torgerson D. A randomized trial of electronic reminders showed a reduction in the time to respond to postal questionnaires. *J Clin Epidemiol* 2011; 64(2):208-212.

(22) Avenell A, Grant AM, McGee M, McPherson G, Campbell MK, McGee MA et al. The effects of an open design on trial participant recruitment, compliance and retention - a randomized controlled trial comparison with a blinded, placebo-controlled design. *Clinical Trials* 2004; 1(6):490-498.

(23) Chaffin M, Valle LA, Funderburk B, Gurwitch R, Silovsky J, Bard D et al. A Motivational Intervention Can Improve Retention in PCIT for Low-Motivation Child Welfare Clients. *Child Maltreatment* 2009; 14(4):356-368.

(24) Cockayne S, Torgerson D. A randomised controlled trial to assess the effectiveness of offering study results as an incentive to increase response rates to postal questionnaires [ISRCTN26118436]. *BMC Medical Research Methodology* 2005; 5(1):34.

(25) Couper PM, Peytchev A, Strecher JV, Rothert K, Anderson J. Following Up Nonrespondents to an Online Weight Management Intervention: Randomized Trial Comparing Mail versus Telephone. *J Med Internet Res* 2007; 9(2):e16.

(26) Cox KL, Burke V, Beilin LJ, Derbyshire AJ, Grove JR, Blanksby BA et al. Short and long-term adherence to swimming and walking programs in older women -- The Sedentary Women Exercise Adherence Trial (SWEAT 2). *Prev Med* 2008; 46(6):511-517.

(27) Dorman P, Slattery J, Farrell B, Dennis MS, Sandercock PA. A randomised comparison of the EuroQol and Short Form-36 after stroke. United Kingdom collaborators in the International Stroke Trial. *BMJ* 1997; 315(7106):461.

(28) Ford ME, Havstad S, Vernon SW, Davis SD, Kroll D, Lamerato L et al. Enhancing Adherence Among Older African American Men Enrolled in a Longitudinal Cancer Screening Trial. *Gerontologist* 2006; 46(4):545-550.

(29) Gates S, Williams M, Withers E, Williamson E, Mt-Isa S, Lamb S. Does a monetary incentive improve the response to a postal questionnaire in a randomised controlled trial? The MINT incentive study. *Trials* 2009; 10(1):44.

(30) Leigh Brown AP, Lawrie H, Kennedy A, Webb A, Torgerson D, Grant A. Cost effectiveness of a prize draw on response to a postal questionnaire:results of a randomised trial among orthopaedic outpatients in Edinburgh. *Journal of Epidemiology and Public Health* 1997; 51:463-464.

For peer review only

(31) Man MS, Tilbrook HE, Jayakody S, Hewitt CE, Cox H, Cross B et al. Electronic reminders did not improve postal questionnaire response rates or response times: a randomized controlled trial. *J Clin Epidemiol* 2011; 64(9):1001-1004.

(32) McCambridge J, Kalaitzaki E, White RI, Khadjesari Z, Murray E, Linke S et al. Impact of Length or Relevance of Questionnaires on Attrition in Online Trials: Randomized Controlled Trial. *J Med Internet Res* 2011; 13(4):e96.

(33) McColl EM, Eccles MPM, Rousseau NSB, Steen INP, Parkin DWD, Grimshaw JMP. From the Generic to the Condition-specific?: Instrument Order Effects in Quality of Life Assessment. [Article]. *Med Care* 2003; 41(7):777-790.

(34) Nakash R. A study of response and non-response to postal questionnaire follow-up in clinical trials. Chapter 6: A radomised controlled trial of a method of improving response to postal questionnaire follow-up in a clinical trial. [University of Warwick; 2007.

(35) Severi E, Free C, Knight R, Robertson S, Edwards P, Hoile E. Two controlled trials to increase participant retention in a randomized controlled trial of mobile phone-based smoking cessation support in the United Kingdom. *Clinical Trials* 2011; 8(5):654-660.

(36) Subar AF, Ziegler RG, Thompson FE, Johnson CC, Weissfeld JL, Reding D et al. Is Shorter Always Better? Relative Importance of Questionnaire Length and Cognitive Ease on Response Rates and Data Quality for Two Dietary Questionnaires. *Am J Epidemiol* 2001; 153(4):404-409.

(37) Sutherland HJ, Beaton M, Mazer R, Kriukov V, Boyd NF. A randomized trial of the total design method for the postal follow-up of women in a cancer prevention trial. *Eur J Cancer Prev* 1996; 5(3):165-168.

(38) Tai SS, Nazareth I, Haines A, Jowett C. A randomized trial of the impact of telephone and recorded delivery reminders on the response rate to research questionnaires. *J Public Health* 1997; 19(2):219-221.

(39) Kenyon S, Pike K, Jones D, Taylor D, Salt A, Marlow N et al. The effect of a monetary incentive on return of a postal health and development questionnaire: a randomised trial [ISRCTN53994660]. *BMC Health Services Research* 2005; 5(1):55.

(40) JR Hughes. Free reprints to increase the return of follow-up questionnaires. <*[11] Journal*> 1989.

(41) The International Stroke Trial (IST): a randomised trial of aspirin, subcutaneous heparin, both, or neither among 19[punctuation space]435 patients with acute ischaemic stroke. *The Lancet* 1997; 349(9065):1569-1581.

(42) Ford ME, Havstad S, Vernon SW, Davis SD, Kroll D, Lamerato L et al. Enhancing Adherence Among Older African American Men Enrolled in a Longitudinal Cancer Screening Trial. *Gerontologist* 2006; 46(4):545-550.

For peer review only - http://bmjopen.bmj.com/site/about/guidelines.xhtml

BMJ Open REVIEW submitted 07.08.2013

(43) Marson A, Appleton R, BakerG, Chadwick D, Doughty J, Eaton B et al. A randomised controlled trial examining the longer-term outcomes of standard versus new antiepileptic drugs The SANAD trial. *NIHR HTA Report* 2007; 11(37).

(44) Nakash R, Hutton J, Jorstad-Stein E, Gates S, Lamb S. Maximising response to postal questionnaires - A systematic review of randomised trials in health research. *BMC Medical Research Methodology* 2006; 6(1):5.

For peer review only

Reference list of host trials within which retention trials were embedded [1-28] .

Reference List

(1) Boyd N, Cousins M, Lockwood G, Tritchler D. Dietary fat and breast cancer risk: The feasibility of a clinical trial of breast cancer prevention. *Lipids* 1992; 27(10):821-826.

(2) Buys SS, Partridge E, Greene MH, Prorok PC, Reding D, Riley TL et al. Ovarian cancer screening in the Prostate, Lung, Colorectal and Ovarian (PLCO) cancer screening trial: Findings from the initial screen of a randomized trial. *Am J Obstet Gynecol* 2005; 193(5):1630-1639.

(3) Chaffin M, Valle LA, Funderburk B, Gurwitch R, Silovsky J, Bard D et al. A Motivational Intervention Can Improve Retention in PCIT for Low-Motivation Child Welfare Clients. *Child Maltreatment* 2009; 14(4):356-368.

(4) Cox KL, Burke V, Beilin LJ, Derbyshire AJ, Grove JR, Blanksby BA et al. Short and long-term adherence to swimming and walking programs in older women -- The Sedentary Women Exercise Adherence Trial (SWEAT 2). *Prev Med* 2008; 46(6):511-517.

(5) Cooke MW, Marsh JL, Clarke M, Nakash R, Jarvis RM, Hutton JL et al. Treatment of severe ankle sprain: a pragmatic randomised controlled trial comparing the clinical effectiveness and cost effectiveness of three types of mechanical ankle support with tubular bandage. The CAST trial. 13, 1-144. 2009. NIHR Health Technology Assessment Programme.
Ref Type: Report

(6) Effect of intravenous corticosteroids on death within 14 days in 10[punctuation space]008 adults with clinically significant head injury (MRC CRASH trial): randomised placebo-controlled trial. *The Lancet* 2004; 364(9442):1321-1328.

(7) Dennis CL, Hodnett E, Kenton L, Weston J, Zupancic J, Stewart DE et al. Effect of peer support on prevention of postnatal depression among high risk women: multisite randomised controlled trial. *BMJ* 2009; 338(jan15_2):a3064.

(8) Eccles M, McColl E, Steen N, Rousseau N, Grimshaw J, Parkin D et al. Effect of computerised evidence based guidelines on management of asthma and angina in adults in primary care: cluster randomised controlled trial. *BMJ* 2002; 325(7370):941.

(9) Free C, Knight R, Robertson S, Whittaker R, Edwards P, Zhou W et al. Smoking cessation support delivered via mobile phone text messaging (txt2stop): a single-blind, randomised trial. *The Lancet* 2011; 378(9785):49-55.

(10) Gail MH, Byar DP, Pechacek TF, Corle DK. Aspects of statistical design for the community intervention trial for smoking cessation (COMMIT). *Control Clin Trials* 1992; 13(1):6-21.

(11) Hughes JR, Hatsukami D, Pickens R, Krahn D, Malin S, Luknic A. Effect of nicotine on the tobacco withdrawal syndrome. *Psychopharmacology (Berl)* 1984; 83(1):82-87.

(12) The International Stroke Trial (IST): a randomised trial of aspirin, subcutaneous heparin, both, or neither among 19[punctuation space]435 patients with acute ischaemic stroke. *The Lancet* 1997; 349(9065):1569-1581.

(13) Kenyon SL, Taylor DJ, Tarnow-Mordi W. Broad-spectrum antibiotics for preterm, prelabour rupture of fetal membranes: the ORACLE I randomised trial. *The Lancet* 2001; 357(9261):979-988.

(14) Kenyon SL, Taylor DJ, Tarnow-Mordi W. Broad-spectrum antibiotics for spontaneous preterm labour: the ORACLE II randomised trial. *The Lancet* 2001; 357(9261):989-994.

(15) Leigh Brown A, Kennedy A, Torgerson D, Campbell J, Webb J, Grant A. The OMENS trial: opportunistic evaluation of musculo-skeletal physician care among orthopaedic outpatients unlikely to require surgery. *Health Bull (Edinb)* 2001; 59(3):198-210.

(16) Marson AG, Al Kharusi AM, Alwaidh M, Appleton R, Baker GA, Chadwick DW et al. The SANAD study of effectiveness of valproate, lamotrigine, or topiramate for generalised and unclassifiable epilepsy: an unblinded randomised controlled trial. *The Lancet* 2007; 369(9566):1016-1026.

(17) Lamb S, Gates S, Underwood M, Cooke M, Ashby D, Szczepura A et al. Managing Injuries of the Neck Trial (MINT): design of a randomised controlled trial of treatments for whiplash associated disorders. *BMC Musculoskeletal Disorders* 2007; 8(1):7.

(18) Murray E, McCambridge J, Khadjesari Z, White I, Thompson S, Godfrey C et al. The DYD-RCT protocol: an on-line randomised controlled trial of an interactive computer-based intervention compared with a standard information website to reduce alcohol consumption among hazardous drinkers. *BMC Public Health* 2007; 7(1):306.

(19) Omenn GS, Goodman GE, Thornquist MD, Balmes J, Cullen MR, Glass A et al. Effects of a Combination of Beta Carotene and Vitamin A on Lung Cancer and Cardiovascular Disease. *N Engl J Med* 1996; 334(18):1150-1155.

For peer review only

For peer review only - http://bmjopen.bmj.com/site/about/guidelines.xhtml

(20) Porthouse J, Sarah C, Christine K, Lucy S, Elizabeth S, Terry A et al. Randomised controlled trial of calcium and supplementation with cholecalciferol (vitamin D3) for prevention of fractures in primary care. *BMJ* 2005; 330.

(21) Oral vitamin D3 and calcium for secondary prevention of low-trauma fractures in elderly people (Randomised Evaluation of Calcium Or vitamin D, RECORD): a randomised placebo-controlled trial. *The Lancet* 2007; 365(9471):1621-1628.

(22) Tai S, Nazareth I, Donegan C, Haines A. Evaluation of General Practice Computer Templates. *Methods Inf Med* 1999; 38:177-181.

(23) The Antiarrhythmics versus Implantable Defibrillators (AVID) Investigators. A Comparison of Antiarrhythmic-Drug Therapy with Implantable Defibrillators in Patients Resuscitated from Near-Fatal Ventricular Arrhythmias. *N Engl J Med* 1997; 337(22):1576-1584.

(24) Tilbrook HE, Cox H, Hewitt CE, Kang'ombe AR, Chuang LH, Jayakody S et al. Yoga for Chronic Low Back PainA Randomized Trial. *Ann Intern Med* 2011; 155(9):569-578.

(25) Rothert K, Strecher VJ, Doyle LA, Caplan WM, Joyce JS, Jimison HB et al. Web-based Weight Management Programs in an Integrated Health Care Setting: A Randomized, Controlled Trial[ast]. *Obesity* 2006; 14(2):266-272.

(26) TOMBOLA Group. Cytological surveillance compared with immediate referral for colposcopy in management of women with low grade cervical abnormalities: multicentre randomised controlled trial. *BMJ* 2009; 339(jul28_2):b2546.

(27) TOMBOLA Group. Biopsy and selective recall compared with immediate large loop excision in management of women with low grade abnormal cervical cytology referred for colposcopy: multicentre randomised controlled trial. *BMJ* 2009; 339(jul28_2):b2548.

(28) UK BEAM Trial Team. United Kingdom back pain exercise and manipulation (UK BEAM) randomised trial: effectiveness of physical treatments for back pain in primary care. *BMJ* 2004;bmj.

For peer review only

For peer review only - http://bmjopen.bmj.com/site/about/guidelines.xhtml

Strategies to improve retention in randomised trials: a systematic review and meta-analysis: tables

Table 1 Characteristics of included incentive trials

Trial	Number randomised	Disease/Condition	Participant in main trials	Setting	Intervention(s)	Control	Outcome attrition trial	Time point used in analysis
**Addition of monetary incentive vs none**								
Bauer 2004 (ab)	300	Treatment smoking dependence	Smokers (Gail 1992)	USA Community	*a)* $10 cheque<br>*b)* $2 cheque<br>Arms combined	No cheque	DNA specimen kit return plus postal questionnaire response	Overall number of kits returned
Gates 2009	2144	Treatment neck injury	Patients with whiplash injury (Lamb 2007)	UK hospital trusts	£5 voucher	No voucher	Postal questionnaire response at 2 weeks	2 week response
Kenyon 2005	722	Treatment preterm labour	Women 7 years post participation in ORACLE trial (Kenyon 2001)	UK secondary care/community	£5 voucher	No voucher	Postal questionnaire response	Overall response
**Addition of offer of monetary incentive/prize draw vs none**								
Khadjesari 2011 (1ac)	1022	Treatment alcohol dependence	Adults scoring +5 on Audit C (Murray 2007)	UK Community: Web based	*a)* Offer £5 voucher<br>*c)* Offer entry £250 prize draw<br>Arms combined	No offer	Web based questionnaire response	Response within 40 days of first reminder
Khadjesari 2011 (2)	2591	Treatment alcohol dependence	Adults scoring +5 on Audit C (Murray 2007)	Community: Web based	Offer £10 Amazon voucher	No offer	Web based questionnaire response	Response within 40 days of first reminder
**Addition of non-monetary incentive vs none**								

For peer review only

Trial	Number randomised	Disease/Condition	Participant in main trials	Setting	Intervention(s)	Control	Outcome attrition trial	Time point used in analysis
Bowen 2000 (abc)	4728	Prevention lung cancer	Adults exposed to smoking and asbestos (Omenn 1996)	USA sites	a) Certificate<br>b) Pin<br><br>c) Pin and certificate<br><br>Arms combined	No certificate /pin	Trial retention	Time from randomisation to first inactivation (stop taking vitamins or placebo) during PRIDE 2 year follow-up
Renfroe 2002 (a)	664	Treatment ventricular fibrillation ventricular tachycardia	Adultscardioverted from VT or resuscitated from VF (AVID 1997)	USA hospital	Certificate of appreciation	No certificate	Postal questionnaire response	Overall response
Sharp 2006 (a)	231	Screening cervical cancer	Women with low grade abnormal cervical smear (TOMBOLA group 2009)	UK primary care	Pen	No pen	Postal questionnaire response	Overall response
Sharp 2006 (b)	232	Screening cervical cancer	Women with low grade abnormal cervical smear (TOMBOLA Group 2009)	UK primary care	Pen	No pen	Postal questionnaire response	Overall response
Sharp 2006 (c)	233	Screening cervical cancer	Women with low grade abnormal cervical smear (TOMBOLA	UK primary care	Pen	No pen	Postal questionnaire response	Overall response

For peer review only - http://bmjopen.bmj.com/site/about/guidelines.xhtml

Trial	Number randomised	Disease/Condition	Participant in main trials	Setting	Intervention(s)	Control	Outcome attrition trial	Time point used in analysis
			Group 2009)					
Sharp 2006 (d)	234	Screening cervical cancer	Women with low grade abnormal cervical smear (TOMBOLA Group 2009)	UK primary care	Pen	No pen	Postal questionnaire response	Overall response
**Addition of offer of non-monetary incentive vs no offer**								
Cockayne 2005	1038	Prevention fracture	Women with hip fracture risk factors micro nutrient trial (Porthouse 2005)	UK primary care	Offer of study results	No offer	Postal questionnaire response	Overall response
Hughes 1989	100	Treatment smoking dependence	Adult smokers (Hughes 1984)	USA community	Offer results reprint	No offer	Postal questionnaire response	Overall response
**Addition of offer of monetary donation to charity vs no offer**								
Khadjesari 2011 (1b)	815	Treatment alcohol dependence	Adults scoring +5 on Audit C (Murray 2007)	Community: on line	Offer £5 charity donation	No offer	Web based questionnaire response	Response within 40 days of first reminder
**Addition of £10 plus offer of £10 vs addition of £5 plus offer of £5**								
Bailey (unpublished)	417	Promotion sexual health	Young people (feasibility study sex un zipped	Community UK on line	Offer of £20 shopping voucher	Offer of £10 shopping	Postal questionnaire response	Response at 3 month follow-up

For peer review only - http://bmjopen.bmj.com/site/about/guidelines.xhtml

Trial	Number randomised	Disease/Condition	Participant in main trials	Setting	Intervention(s)	Control	Outcome attrition trial	Time point used in analysis
			trial)			voucher		
**Addition of £20 voucher offer vs addition of £10 voucher offer**								
Bailey (unpublished)	485	Promotion sexual health	Young (feasibility study sex un zipped trial)	Community UK on line	£10 shopping voucher + offer of £10 shopping voucher	£5 shopping voucher + offer of £5 shopping voucher	Postal questionnaire response and chlamydia kit return	Response at 3 month follow-up
**Addition of monetary incentive vs offer of entry into prize draw**								
Kenton 2007 (a)	147	Prevention post natal depression	Women postpartum at high risk of postnatal depression (Dennis 2009)	Canada community	$2 coin	Draw for $50 gift voucher	Postal questionnaire response	Overall Response
Kenton 2007 (b)	150	Prevention post natal depression	Women postpartum at high risk of postnatal depression (Dennis 2009)	Canada community	$2 coin	Draw for $50 gift voucher	Postal questionnaire response	Overall response
**Offer of prize draw entry vs no offer**								
Leighbrown 1997	1307	Clinical management orthopaedics	Adults non-surgical musculoskeletal conditions (Leigh Brown 2001)	UK Hosp out patients department	Aware Offer of monthly prize draw of £25 gift voucher	No offer	Postal questionnaire response after first and 2nd reminder	No data available

For peer review only - http://bmjopen.bmj.com/site/about/guidelines.xhtml

Table 2 Characteristics of included communication trials

Trial	Number randomised	Main/ Attrition trial area	Participants	Setting	Intervention	Control	Outcome attrition trial	Time point used in analysis
**Enhanced letter vs standard letter**								
Renfroe 2002 (c)	664	Treatment ventricular fibrillation ventricular tachycardia	Adults cardioverted from VT or resuscitated from VF (AVID Investigators 1997)	USA hospital	Cover letter signed by physician	Cover letter signed by coordinator	Postal questionnaire response	Overall response
Marson 2007	1815	Treatment epilepsy	Adults with epilepsy mean SANAD trial. (Marson 2007)	UK hospital outpatient departments	Letter explaining the approximate time needed to complete questionnaire	Standard letter	Postal questionnaire response	Overall response
**Total design postal method for postal questionnaires vs customary method**								
Sutherland 1 996	226	Prevention breast cancer	Women with 50% breast volume dysplasia (Boyd 1992)	Canada Hosp clinic	Total design method for postal follow-up	Customary method for postal follow-up	Postal questionnaire response	Response at day 70.
**Priority vs regular post**								
Renfroe 2002 (b)	664	Treatment ventricular fibrillation ventricular tachycardia	Adults cardioverted from VT or resuscitated from VF (AVID) Investigators 1997)	USA hospital	Overnight questionnaire delivery	Standard questionnaire delivery	Postal questionnaire response	Overall response No of questionnaires returned

For peer review only - http://bmjopen.bmj.com/site/about/guidelines.xhtml

Trial	Number randomised	Main/ Attrition trial area	Participants	Setting	Intervention	Control	Outcome attrition trial	Time point used in analysis
Sharp 2006 (*e*)	233	Screening cervical cancer	Women with low grade abnormal cervical smear (TOMBOLA Group 2009)	UK primary care	1st class outward post	2nd class outward post	Postal questionnaire response	Overall response
Sharp 2006 (*f*)	231	Screening cervical cancer	Women with low grade abnormal cervical smear (TOMBOLA Group 2009)	UK primary care	1st class outward post	2nd class outward post	Postal questionnaire response	Overall response
Sharp 2006 (*g*)	240	Screening cervical cancer	Women with low grade abnormal cervical smear (TOMBOLA Group 2009)	UK primary care	Stamped reply envelope	Business reply envelope	Postal questionnaire response	Overall response
Sharp 2006 (*h*)	223	Screening cervical cancer	Women with low grade abnormal cervical smear (TOMBOLA Group 2009)	UK primary care	Stamped reply envelope	Business reply envelope	Postal questionnaire response	Overall response
Kenton 2007 (*c*)	149	Screening post natal depression	Women postpartum at high risk of postnatal depression (Dennis 2009)	Canada community	Priority outward mail	Regular outward mail	Postal questionnaire response	Overall response
Kenton 2007	148	Screening	Women postpartum at	Canada	Priority	Regular outward mail	Postal	Overall

For peer review only - http://bmjopen.bmj.com/site/about/guidelines.xhtml

Trial	Number randomised	Main/ Attrition trial area	Participants	Setting	Intervention	Control	Outcome attrition trial	Time point used in analysis
(d)		post natal depression	high risk of postnatal depression (Dennis 2009)	community	outward mail		questionnaire response	response
**Additional reminder vs usual follow-up procedures**								
Ashby 2011	148	Prevention migraine	Adults history of two migraine attacks	UK community	Electronic reminder (email and /or SMS text)	No electronic reminder	Postal questionnaire response	Response at 40 days
Maclennan unpublished	753	Prevention fracture	Adults with history of osteoporotic fracture (RECORD Trial Group 2005)	UK hospital	Telephone reminder (before receiving first reminder)	No telephone reminder	Postal questionnaire response	Overall response Response rate
Nakash unpublished	298	Treatment of ankle injury	Cast trial: Adults with acute severe ankle sprain (Cooke 2009)	UK Accident and emergency departments	Trial calendar with questionnaire. due dates	No calendar	Postal questionnaire response at 4, 12 weeks, and 9 months.	Response at 4 weeks
Severi 2011 (1)	1950	Treatment smoking dependence	Adult smokers willing to quit in Txt2stop (Free 2011)	UK community	Text message and fridge magnet emphasising social benefits of study	Text message 3 days after questionnaire sent reminding questionnaire is due	Postal questionnaire response	Response at 30 weeks from randomisation.

For peer review only - http://bmjopen.bmj.com/site/about/guidelines.xhtml

Trial	Number randomised	Main/ Attrition trial area	Participants	Setting	Intervention	Control	Outcome attrition trial	Time point used in analysis
					participation.			
Severi 2011 (2)	127	Treatment smoking dependence	Adult smokers willing to quit in Txt2stop (Free 2011)	UK community	Telephone reminder from principle investigator that participants six weeks overdue returning their specimens	Standard text and no phone call from principle investigator	Return of cotinine samples	Completed cotinine sample follow-up for Txt2stop at end of May 2009
Man 2011	125	Treatment back pain	Adults with back pain (Tilbrook 2011)	UK primary care	SMS text reminder message as follow-up questionnaire sent out	No SMS text message	Postal questionnaire response	Overall Response rate
**Monthly reminder of upcoming assessment to site vs usual reminders**								
Land 2007	429	Treatment breast cancer	Women with ductal carcinoma in situ (unpublished)	Hospital sites USA, Canada, Puerto Rico	Prospective monthly reminder of upcoming assessments to sites	No extra reminders to sites	Postal questionnaire response	Overall Response rate
**Early vs late administartion of questionnaire**								
Renfroe 2002 (d)	664	Treatment ventricular fibrillation ventricular tachycardia	Adults cardioverted from VT or resuscitated from VF (AVID) Investigators 1997)	USA hospital	Questionnaire sent 2-3 weeks after last AVID follow-up visit	Questionnaire sent 1-4 months after last AVID follow-up visit	Postal questionnaire response	Overall response Number of questionnaires returned
**Recorded delivery vs telephone reminder**								

For peer review only - http://bmjopen.bmj.com/site/about/guidelines.xhtml

Trial	Number randomised	Main/ Attrition trial area	Participants	Setting	Intervention	Control	Outcome attrition trial	Time point used in analysis
Tai 1997	192	Clinical managemen t asthma and diabetes	Adults with asthma or diabetes (Tai 1999)	UK primary care	Recorded delivery reminder	Telephone reminder	Postal questionnaire response	Overall response Number of questionnaires returned used
**Telephone interview vs questionnaire and monetary incentive**								
Couper 2007	700	Weight managemen t	Adults with BMI >25 (Rothert 2006	USA community web based	Telephone interview by trained interviewer	Postal questionnaires with $5 bill	Post and telephone questionnaire response	Response at 6 months

For peer review only - http://bmjopen.bmj.com/site/about/guidelines.xhtml

Table 3 Characteristics of included trials evaluating new questionnaire strategies

Trial	Number of participants	Main/ attrition trial area	Participants	Setting	Intervention	Control	Outcome attrition trial	Time point used in analysis
**Short versus long questionnaire**								
Dorman 1997	2253	Treatment Stroke	Stroke patients (International Stroke Trial 1997)	UK hospital	Short EUROQOL questionnaire	Long SF 36 questionnaire	Postal questionnaire response after first mail out and reminder	Response at first time point.
Edwards 2001 unpublished	99	Treatment head injury	Head injury patients (CRASH Trial 2004)	UK hospital intensive care units	1-page, 7 question functional dependence questionnaire	3-page, 16 question functional dependence questionnaire.	Postal questionnaire response	Response at 3 months
Svoboda 2001 unpublished	91	Treatment head injury	Head injury patients (CRASH Trial 2004)	Czech republic hospital intensive care units	1-page, 7 question functional dependence questionnaire	3-page, 16 question functional dependence questionnaire.	Postal questionnaire response	Response at 3 months
Mc Cambridge 2011 1b	2835	Treatment alcohol dependence	Adults scoring +5 on Audit C (Murray 2007)	Community web based	Audit Short (alcohol use disorders questionnaire)<br><br>+<br><br>LDQ (Leeds dependancy	APQ (alcohol problems questionnaire)	Web based questionnaire response at 1 month and 3 months	Response at 1 month

For peer review only - http://bmjopen.bmj.com/site/about/guidelines.xhtml

Trial	Number of participants	Main/ attrition trial area	Participants	Setting	Intervention	Control	Outcome attrition trial	Time point used in analysis
					questionnaire)			
Mc Cambridge 2011 2b	1999	Treatment Alcohol dependence	Adults scoring +5 on Adults scoring +5 on Audit C (Murray 2007)	Community web based	Audit Short (alcohol use disorders questionnaire) + LDQ (Leeds dependancy questionnaire)	APQ (alcohol problems questionnaire)	Web based questionnaire response at 3 month and 12 months	Response at 3 months
**Long and clear versus short and condensed questionnaires**								
Subar 2001	900	Screening prostate, lung, ovarian, colorectal cancer	Adults in PLCO trial (Prorok 2000)	USA sites	DHQ (36-page food frequency questionnaire)	PLCO (16-page food frequency questionnaire)	Postal questionnaire/ response on site completion	Overall response
**Question order: condition first vs generic first questions**								
Mc Coll 2003 (1)	4751	Clinical management asthma	Adult with asthma in COGENT Trial: (Eccles 2002)	UK primary care	Condition specific questions first followed by generic	Generic questions followed by condition specific	Postal questionnaireresponse	Overall response
Mc Coll 2003 (2)	4684	Clinical management angina	Adult with angina in the COGENT Trial: (Eccles 2002)	UK primary care	Condition specific questions followed by generic	Generic questions followed by condition specific	Postal questionnaire response	Overall response

For peer review only - http://bmjopen.bmj.com/site/about/guidelines.xhtml

Trial	Number of participants	Main/ attrition trial area	Participants	Setting	Intervention	Control	Outcome attrition trial	Time point used in analysis
Letley unpublished. No data available	Data not available	Treatment back pain	Adults with low back pain (UK BEAM trial team 2004)	UK primary care	23 page self-completion questionnaire Roland disability questionnaire at front and SF 36 at back	vice versa	Questionnaire response	No data
**Questionnaire: relevant versus less relevant to condition**								
Mc Cambridge 2011 1a	1892	Treatment alcohol dependence	Adults scoring +5 on Audit C (Murray 2007)	Community web based	Alcohol problem questionnaire (APQ)23 items	Core OM Mental health assessment 23/34 items	Web based questionnaire response at 1 and 3 months	Response at 1 month
Mc Cambridge 2011 2a	2001	Treatment alcohol dependence	Adults scoring +5 on Audit C (Murray 2007)	Community web based	Audit Short (alcohol use disorders questionnaire) + LDQ (Leeds dependancy questionnaire)	Core OM Mental health assessment 10 items	Web based questionnaire response at 3 month and 12 months	Response at 3 months

For peer review only - http://bmjopen.bmj.com/site/about/guidelines.xhtml

Table 4 Characteristics of other trials

Trial	Number of participants	Main/ attrition trial area	Participants	Country	Behavioural strategy	Control arms	Outcome attrition trial	Time point used in analysis
**Motivation vs information**								
Cox 2008	120	Exercise improvement	Sedentary Women in SWEAT 2 Trial (Cox 2008)	Australia Community	Motivational workshops and newsletters	Information sheets and newsletters	Program and trial retention at 6 and 12 months	6 month and 12 month data. Data for 6 months used
Chaffin 2009	153	Parenting improvement	Adults referred for parenting improvement (Chaffin 2009)	USA community	Self-motivation information	Standard information	Program attendance/ trial retention	Retention at 12 weeks
**Case management vs usual follow-up**								
Ford 2006	703	Screening prostate, lung, ovarian, colorectal cancer	Adults in the PLCO screening trial (Prorok 2000)	USA sites	In-depth case management	Regular trial procedures	Attendance at face to face cancer screening	Retention at 3 years
**Open vs blind trial design**								
Avenell 2004	538	Prevention fracture	Adults with history of osteoporotic fracture in the RECORD micronutrient trial (RECORD Trial Group 2005)	UK hospital	Open trial design	Blind trial design	Postal questionnaire response at 4, 8,12 months	Response at12 months

For peer review only - http://bmjopen.bmj.com/site/about/guidelines.xhtml

Table 5 Absolute benefit of effective strategies to improve retention

**Example of proportion of questionnaires returned in control arm**			**30%**	**40%**	**50%**	**60%**	**70%**	**80%**	**90%**
Strategy to improve retention	RR	1/ RR							
Addition of monetary incentive versus no incentive	1.18	0.847	107	92	76	61	5	3	2
Addition of offer of monetary incentive/prize draw versus no offer	1.25	0.800	140	120	100	80	60	40	20
Addition of higher value monetary incentive versus addition of lower amount	1.12	0.890	77	66	55	44	33	22	11

For peer review only

For peer review only - http://bmjopen.bmj.com/site/about/guidelines.xhtml

Figure 1 Incentive strategies: main analysis addition of incentive versus no incentive

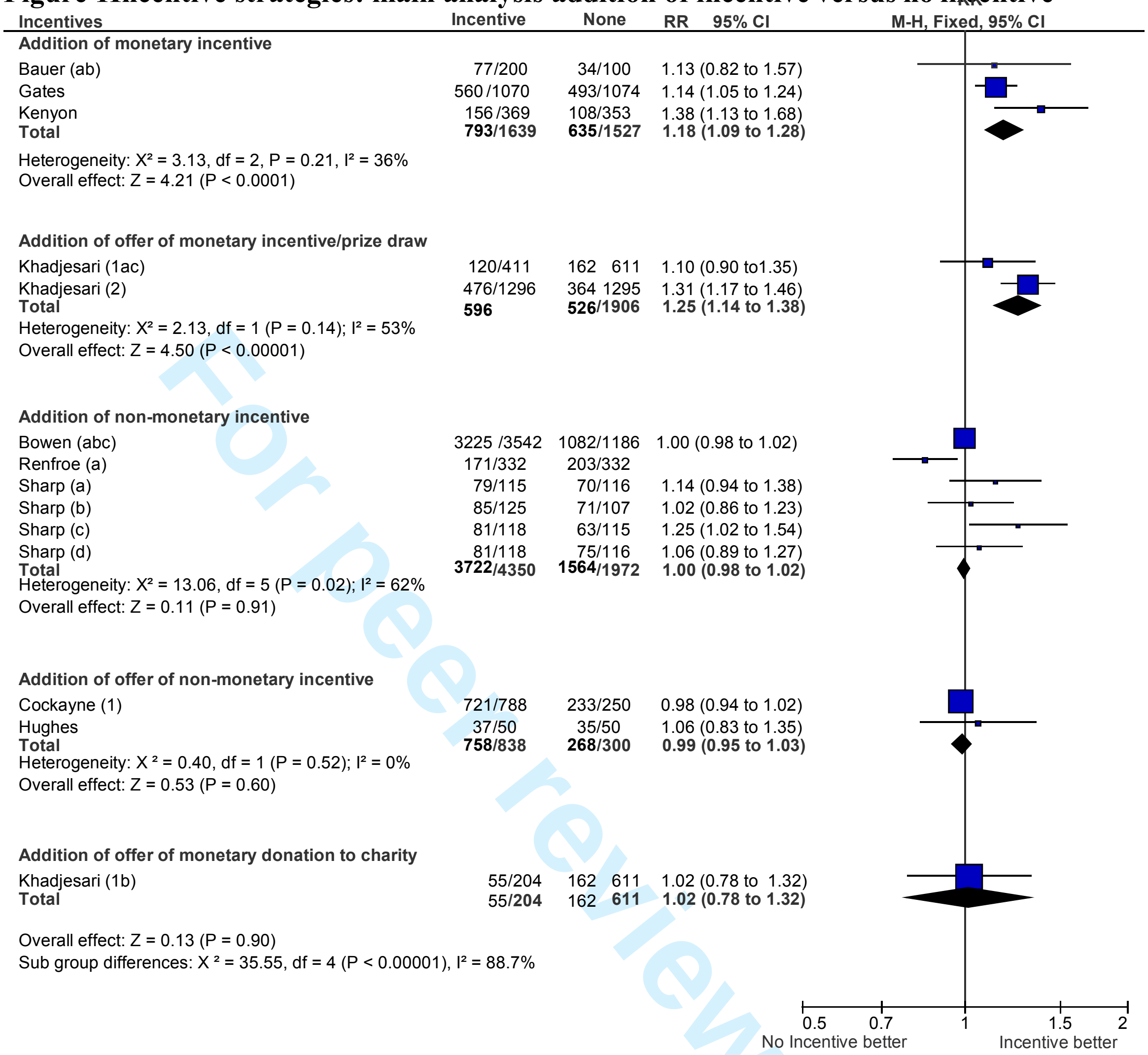

Fig 1b Incentives: addition of £20 vs £10 incentive

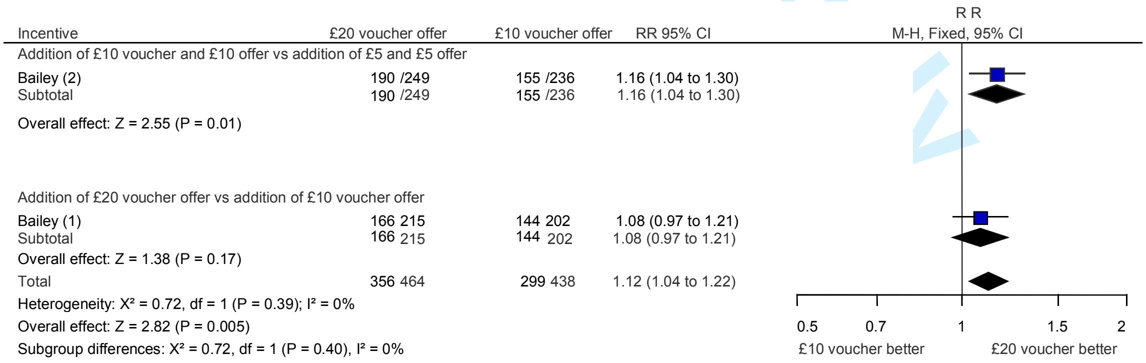

Fig 1c Incentives addition of: monetary incentive vs offer of entry into prize draw

Incentive	Monetary incentive	Entry into draw	RR Fixed, 95% CI
**Addition of monetary incentive vs offer of entry into prize draw**			
Kenton (a)	58 /72	53 /75	1.14 (0.95 to 1.37)
Kenton (b)	55 /77	55 /73	0.95 (0.78 to 1.15)
**Total**	113 /149	108 /148	**1.04 (0.91 to 1.19)**

Heterogeneity: Chi² = 1.83, df = 1 (P = 0.18); I² = 45%
Overall effect: Z = 0.58 (P = 0.56)

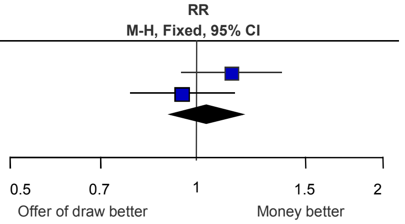

Fig 2a Communication strategies: enhanced vs standard letter

Letters	Enhanced	Standard	RR 95% CI
**Enhanced vs standard**			
Renfroe (c)	180 /332	181 /332	0.99 (0.87 to 1.14)
Marson	756 /891	775 /924	1.01 (0.97 to 1.05)
**Total**	**936** /1223	**956** /1256	**1.01 (0.97 to 1.05)**

Heterogeneity: X² = 0.06, df = 1 (P = 0.80); I² = 0%
Overall effect: Z = 0.39 (P = 0.70)

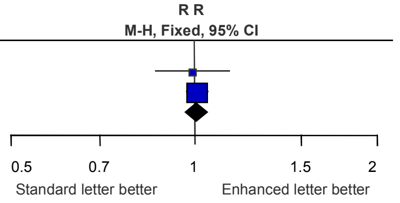

Fig 2b Communication: total design vs customary post

Postal method	Total design	Customary post	RR, 95% CI
**Total design for postal questionnaires vs customary method**			
Sutherland	100 /113	70/113	1.43 (1.22 to 1.67)
**Total**	100 /113	70 /113	**1.43 (1.22 to 1.67)**

Overall effect: Z = 4.39 (P < 0.0001)

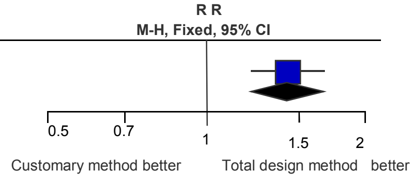

Fig 2c Communication: post priority vs regular post

Post type	Priority	Regular	RR, 95% CI
**Priority vs regular post**			
Renfroe (b)	188 /332	173/332	1.09 (0.95 to 1.25)
Sharp (f)	70 /116	63/115	1.10 (0.88 to 1.38)
Sharp (e)	79 /115	81/118	1.00 (0.84 to 1.19)
Sharp (h)	70 /116	71/107	0.91 (0.74 to 1.11)
Sharp (g)	79 /115	85/125	1.01 (0.85 to 1.20)
Kenton (d)	55 /73	53 /75	1.07 (0.88 to 1.30)
Kenton (c)	55 /77	58 /72	0.89 (0.74 to 1.06)
**Total**	**596** /944	**584**/944	**1.02 (0.95 to 1.09)**

Heterogeneity: X² = 5.08, df = 6 (P = 0.53); I² = 0%
Overall effect: Z = 0.59 (P = 0.55)

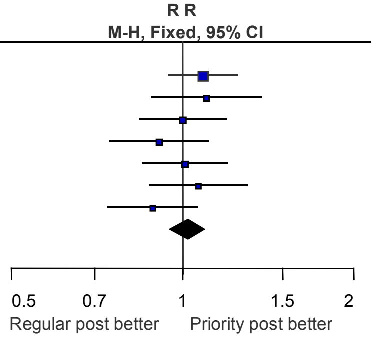

Fig 2d Communication: additional reminders to participants vs usual follow-up

Participant reminder	Additional reminder Response/	No reminders sent	Usual follow-up Response/	No reminders sent	RR 95% CI
**Extra reminder vs usual follow-up**					
Ashby	68	74	64	74	1.06 (0.95 to 1.19)
MacLennan	267	390	227	363	1.09 (0.99 to 1.22)
Man	54	62	53	63	1.04 (0.90 to 1.20)
Nakash	117	152	114	146	0.99 (0.87 to 1.11)
Severi (2)	20	65	20	62	0.95 (0.57 to 1.59)
Severi (1)	813	976	801	974	1.01 (0.97 to 1.05)
**Total**	1339	**1719**	1279	**1682**	**1.03 (0.99 to 1.06)**

Heterogeneity: X² = 2.78, df = 5 (P = 0.73); I² = 0%
Overall effect: Z = 1.52 (P = 0.13)

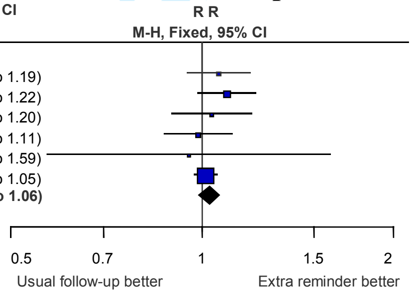

Fig 2e Communication: telephone survey versus monetary incentive and questionnaire

Study or Subgroup	Telephone survey Events	Telephone survey Total	Monetary + questionnaire Events	Monetary + questionnaire Total	Risk Ratio M-H, Fixed, 95% CI
**Telephone survey vs monetary incentive and questionnaire**					
Couper 2007	170	300	210	400	1.08 [0.94, 1.24]
**Subtotal (95% CI)**		**300**		**400**	**1.08 [0.94, 1.24]**
Total events	170		210		

Heterogeneity: Not applicable
Test for overall effect: Z = 1.10 (P = 0.27)

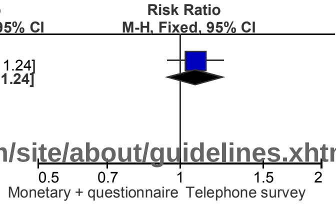

For peer review only - http://bmjopen.bmj.com/site/about/guidelines.xhtml

Fig 3 Questionnaires: new format vs standard format

Questionnaires	New questionnaires Response /No sent	Standard questionnaires Response /No sent	RR 95% CI
**Short vs long**			
Edwards	31/50	35/49	0.87 (0.66 to 1.15)
Dorman	747/1125	679/1128	1.10 (1.04 to 1.17)
Svoboda	29/45	31/46	0.96 (0.71 to 1.29)
Mc Cambridge 2(b)	653/1333	316/666	1.03 (0.94 to 1.14)
Mc Cambridge 1(b)	1049/1888	529/947	0.99 (0.93 to 1.07)
**Total**	2509/**4441**	1590/**2836**	**1.04 (1.00 to 1.08)**
Heterogeneity: X^2 = 6.87, df = 4 (P = 0.14); I^2 = 42%			
Overall effect: Z = 1.82 (P = 0.07)			
**Long and clear vs short and condensed**			
Subar	369/450	367/450	1.01 (0.95 to 1.07)
**Total**	369/**450**	367/**450**	**1.01 (0.95 to 1.07)**
Overall effect: Z = 0.17 (P = 0.86)			
**Question order: condition questions first vs generic first**			
Mc Coll (2)	1779/2363	1738/2321	1.01 (0.97 to 1.04)
Mc Coll (1)	1522/2382	1537/2369	0.98 (0.94 to 1.03)
**Total**	3301/**4745**	3275/**4690**	**1.00 (0.97 to1.02)**
Heterogeneity: X^2 = 0.59, df = 1 (P = 0.44); I^2 = 0%			
Overall effect: Z = 0.31 (P = 0.75)			
**Questionnaire relevant versus less relevant to condition**			
Mc Cambridge 2(a)	653/1333	308/668	1.06 (0.96 to 1.17)
Mc Cambridge 1(a)	529/947	489/945	1.08 (0.99 to 1.17)
**Total**	1182/**2280**	797/**1613**	**1.07 (1.01 to 1.14)**
Heterogeneity: X^2 = 0.06, df = 1 (P = 0.81); I^2 = 0%			
Overall effect: Z = 2.12 (P = 0.03)			
Subgroup differences: X^2 = 6.13, df = 3 (P = 0.11), I^2 = 51.0%			

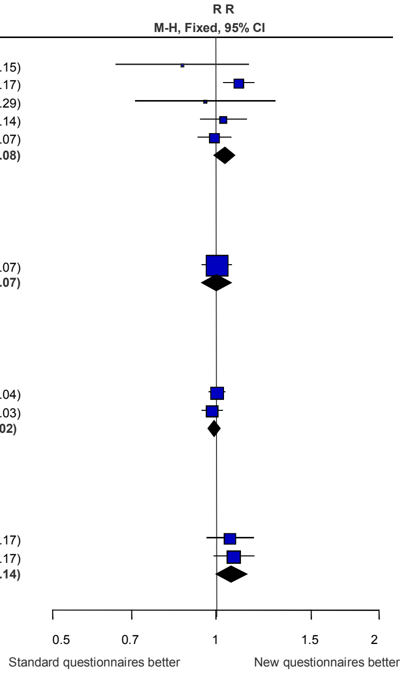

For peer review only

Fig 4 PRISMA diagram

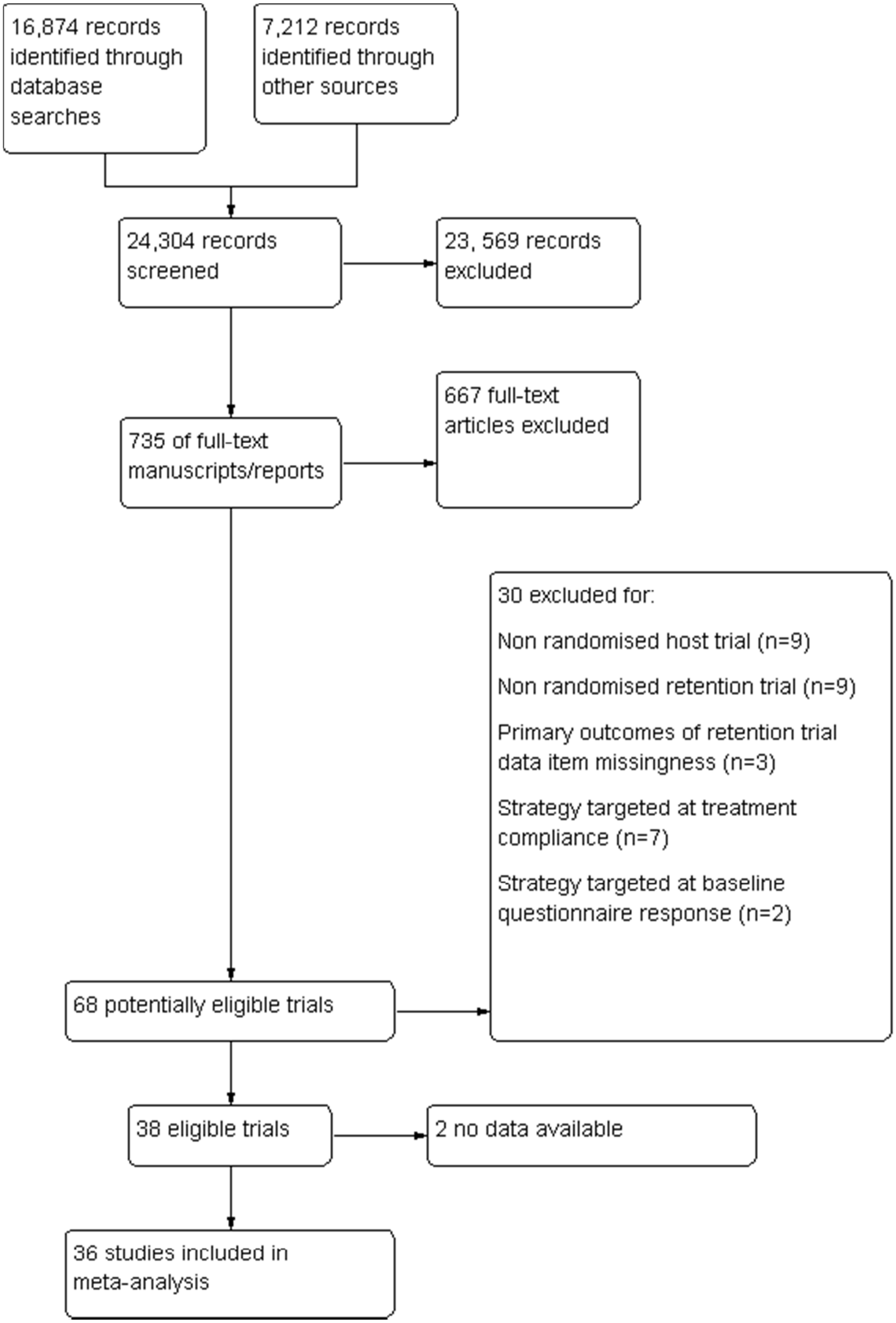

Figure 5 Exploratory analyses for the main incentives analysis (web appendix)

Study or Subgroup	Incentive Events	Incentive Total	No incentive Events	No incentive Total	Weight	Risk Ratio M-H, Fixed, 95% CI
**3.1.1 Addition of monetary incentive**						
Bauer 2004a	43	100	34	100	5.1%	1.26 [0.89, 1.80]
Bauer 2004b	34	100	34	100	5.1%	1.00 [0.68, 1.47]
Gates 2009	560	1070	493	1074	73.4%	1.14 [1.05, 1.24]
Kenyon 2005	156	369	108	353	16.5%	1.38 [1.13, 1.68]
**Subtotal (95% CI)**		**1639**		**1627**	**100.0%**	**1.18 [1.09, 1.27]**
Total events	793		669			
Heterogeneity: Chi² = 3.91, df = 3 (P = 0.27); I² = 23%						
Test for overall effect: Z = 4.24 (P < 0.0001)						
**3.1.2 Offer of monetary incentive**						
Khadjesari 2011 1a	66	206	162	611	15.5%	1.21 [0.95, 1.53]
Khadjesari 2011 1c	54	205	162	611	15.4%	0.99 [0.76, 1.29]
Khadjesari 2011 2	476	1296	364	1295	69.1%	1.31 [1.17, 1.46]
**Subtotal (95% CI)**		**1707**		**2517**	**100.0%**	**1.24 [1.13, 1.37]**
Total events	596		688			
Heterogeneity: Chi² = 3.57, df = 2 (P = 0.17); I² = 44%						
Test for overall effect: Z = 4.49 (P < 0.00001)						
**3.1.3 Addition of non monetary incentive**						
Bowen 2000a	988	1092	1082	1186	21.7%	0.99 [0.97, 1.02]
Bowen 2000b	1120	1211	1082	1186	22.9%	1.01 [0.99, 1.04]
Bowen 2000c	1117	1239	1082	1186	23.1%	0.99 [0.96, 1.01]
Renfroe 2002a	171	332	203	332	4.2%	0.84 [0.74, 0.96]
Sharp 2006a	79	115	70	116	1.5%	1.14 [0.94, 1.38]
Sharp 2006b	1201	1239	1062	1092	23.6%	1.00 [0.98, 1.01]
Sharp 2006c	81	118	75	116	1.6%	1.06 [0.89, 1.27]
Sharp 2006d	81	118	63	115	1.3%	1.25 [1.02, 1.54]
**Subtotal (95% CI)**		**5464**		**5329**	**100.0%**	**1.00 [0.98, 1.01]**
Total events	4838		4719			
Heterogeneity: Chi² = 15.47, df = 7 (P = 0.03); I² = 55%						
Test for overall effect: Z = 0.38 (P = 0.70)						

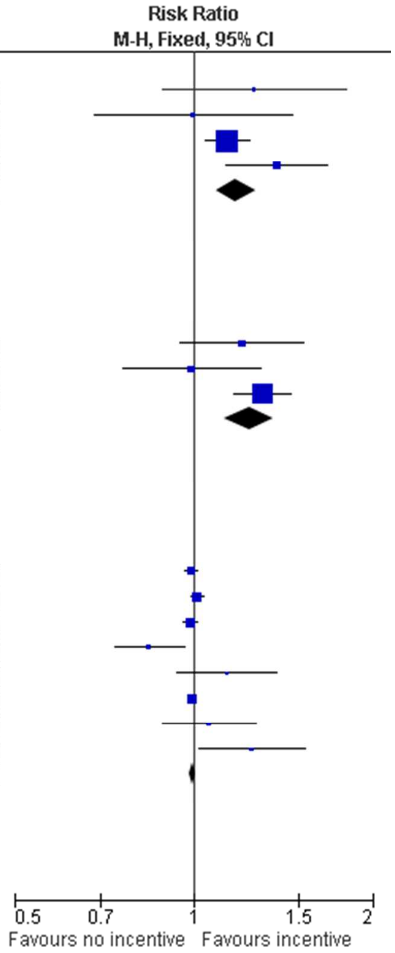

Test for subgroup differences: Chi² = 37.44, df = 2 (P < 0.00001), I² = 94.7%

For peer review only

Strategies to improve retention in randomised trials: a Cochrane systematic review and meta-analysis

Journal:	*BMJ Open*
Manuscript ID:	bmjopen-2013-003821.R1
Article Type:	Research
Date Submitted by the Author:	22-Nov-2013
Complete List of Authors:	Brueton, Valerie; MRC Clinical Trials Unit, Meta-analysis Group<br>Tierney, Jayne; MRC Clinical Trials Unit, Meta-analysis Group<br>Stenning, Sally; MRC Clinical Trials Unit, Cancer and non infections Group<br>Meredith, Sarah; MRC Clinical Trials Unit,<br>Harding, Seeromanie; MRC, Social and Public Health Sciences Unit<br>Nazareth, Irwin; UCL, Primary Care and Population Health<br>Rait, Greta; UCL, Primary Care and Population Health
**Primary Subject Heading**:	Evidence based practice
Secondary Subject Heading:	Research methods
Keywords:	Retention, Randomised trials, Strategies

SCHOLARONE™
Manuscripts

Title page

Strategies to improve retention in randomised trials: a Cochrane systematic review and meta-analysis[1]

Brueton VC

Research Scientist, Meta-analysis Group
MRC Clinical Trials Unit at UCL
Aviation House
125 Kingsway
London WC2B 6NH
e-mail: vcb@ctu.mrc.ac.uk

Tierney JF

Head of Meta-analysis Group
MRC Clinical Trials Unit at UCL
Aviation House
125 Kingsway
London WC2B 6NH

Stenning S

Senior Statistician
MRC Clinical Trials Unit at UCL
Aviation House
125 Kingsway
London WC2B 6NH

Meredith S

Deputy Director
MRC Clinical Trials Unit at UCL
Aviation House
125 Kingsway
London WC2B 6NH

Harding S

Program leader: Ethnicity and Health
MRC Social & Public Health Sciences Unit
4 Lilybank Gardens
Glasgow G12 8RZ

[1] This article is based on a Cochrane Review to be published in the Cochrane Database of Systematic Reviews (CDSR) 2013, Issue X, DOI:10.1002/14651858.CD00xxxx (see www.thecochranelibrary.com for information)

For peer review only

BMJ Open REVIEW submitted 07.08.2013

Nazareth I

Professor of Primary Care
PRIMENT Clinical Trials Unit
Research Department of Primary care and Population Health
UCL Medical School
Rowland Hill Street
London
NW3 2PF

Rait G

Clinical Senior Lecturer
PRIMENT Clinical Trials Unit
Research Department of Primary care and Population Health
UCL Medical School
Rowland Hill Street
London
NW3 2PF

For peer review only

BMJ Open REVIEW submitted 07.08.2013

Objective

To quantify the effect of strategies to improve retention in randomised trials.

Design

Systematic review and meta-analysis.

Data sources

Sources searched: MEDLINE, EMBASE, PsycINFO, DARE, CENTRAL, CINAHL, C2-SPECTR, ERIC, PreMEDLINE, Cochrane Methodology Register, Current Controlled Trials metaRegister, WHO trials platform, Society for Clinical Trials (SCT) conference proceedings, and a survey of all UK clinical trial research units.

Review methods

Included trials were randomised evaluations of strategies to improve retention embedded within host randomised trials. The primary outcome was retention of trial participants. Data from trials were pooled using the fixed-effect model. Subgroup analyses were used to explore heterogeneity and to determine whether there were any differences in effect by type of strategy.

Results

38 retention trials were identified. Six broad types of strategies were evaluated. Strategies that increased postal questionnaire responses were: adding i.e. giving a monetary incentive (RR 1.18; 95% CI 1.09-1.28) and higher valued incentives (RR 1.12; 95% CI 1.04-1.22). Offering a monetary incentive i.e. incentive given on receipt of a completed questionnaire, also increased electronic questionnaire response (RR 1.25; 95% CI 1.14-1.38). The evidence for shorter questionnaires (RR 1.04; 95% CI 1.00-1.08) and questionnaires relevant to the disease/condition (RR 1.07; 95% CI 1.01-1.14) is less clear.

Based on the results of single trials the following strategies appeared effective at increasing questionnaire response: recorded delivery of questionnaires (RR 2.08; 95% CI 1.11-3.87); a "package" of postal communication strategies (RR 1.43; 95% CI 1.22-1.67), and an open trial design (RR 1.37; 95% CI 1.16 -1.63). There is no good evidence that the following strategies impact on trial response/retention: adding a non-monetary incentive (RR=1.00; 95% CI 0.98-1.02); offering a non-monetary incentive (RR=0.99; 95% CI 0.95-1.03); "enhanced" letters (RR=1.01; 95% CI 0.97-1.05); monetary incentives compared to offering prize draw entry (RR=1.04; 95% CI 0.91- 1.19); priority postal delivery (RR=1.02; 95% CI 0.95 - 1.09); behavioural motivational strategies (RR= 1.08; 95% CI 0.93-1.24); additional reminders to

For peer review only

participants (RR=1.03; 95% CI 0.99-1.06); and questionnaire question order (RR=1.00, 0.97-1.02).

Also based on single trials, these strategies do not appear effective: a telephone survey compared to a monetary incentive plus questionnaire (RR=1.08; 95% CI 0.94-1.24); offering a charity donation (RR =1.02, 95% CI; 0.78-1.32); sending sites reminders (RR= 0.96; 95% CI 0.83-1.11); sending questionnaires early (RR=1.10; 95% CI 0.96-1.26); longer and clearer questionnaires (RR= 1.01, 0.95-1.07) and participant case management by trial assistants (RR=1.00; 95% CI 0.97-1.04).

Conclusion

Most trials evaluated questionnaire response rather than ways to improve participants return to site for follow-up. Monetary incentives and offers of monetary incentives increase postal and electronic questionnaire response. Some strategies need further evaluation. Application of these results would depend on trial context and follow-up procedures.

For peer review only

BMJ Open REVIEW submitted 07.08.2013

Article summary

Article focus

Loss to follow-up in randomised trials can cause bias and loss of power.

Many strategies are routinely used in an attempt to improve retention in randomised trials.

The effect of strategies used to improve retention in randomised trials has not been formally evaluated until now. This systematic review identifies strategies that have been evaluated in randomised trials and quantifies the effect of these strategies to improve retention in randomised trials.

Key messages

This is the first systematic review to evaluate the effect of strategies to improve retention in randomised trials.

Effective strategies for increasing postal questionnaire response were: monetary incentives, offers of monetary incentives, and higher valued incentives.

Strategies that encourage participant to return to sites for follow-up visits and monitoring are particularly needed. Other strategies need further evaluation.

Such evaluations need to be rigorous and adequately reported

Strengths and limitations of this study

This is the most comprehensive review of strategies specifically designed to improve retention in randomised trials, including many unpublished trials and data.

Although our searches were extensive, some less well reported, on-going, or unpublished trials, or trials conducted outside the UK might have been missed.

For peer review only

For peer review only - http://bmjopen.bmj.com/site/about/guidelines.xhtml

Introduction

Loss of participants during study follow-up can introduce bias and reduce power affecting the generalisability, validity, and reliability of results[1;2] . If losses are fewer than 5% they may lead to minimum bias, while 20% loss can threaten trial validity[2]. While missing data from losses to follow-up can be dealt with statistically, the risk of bias can remain[3].

Trialists adopt various strategies to try to improve retention and generate maximum data return or compliance to follow-up procedures. These strategies are designed to motivate and keep participants or site clinicians engaged in a trial, but many are untested[4;5]. A systematic review of strategies to retain participants cohort studies suggests that providing incentives can improve retention[6]. Edwards systematic review on methods to increase response rates to postal and electronic questionnaires across a range of study types found that including monetary incentives, keeping the questionnaire short and contacting people before questionnaires were sent were ways to increase response rates[7]. However, heterogeneity of effects was an issue and it is unclear which strategies are applicable to randomised trials. Moreover, reasons for loss to follow-up in cohort studies and surveys may differ from randomised trials. In trials, participants may be randomised to a study arm that is not their preferred choice and so strategies that improve retention in other study types cannot necessarily be extrapolated to randomised trials.

As loss to follow-up can compromise the validity of findings from randomised trials, delay results and potentially increase trial costs, we conducted a systematic review to assess the effect of strategies to improve retention in randomised trials.

Methods

The methods were pre-specified in the Cochrane review protocol[8].

Trials included

We included randomised trials that compared strategies to increase participant retention embedded in "host" randomised trials across disease areas and settings. These strategies should have been designed for use after participants were recruited and randomised. Retention trials embedded in cohort studies and surveys were excluded.

For peer review only

Identification of retention trials

We searched MEDLINE (1950 to May 2012), EMBASE (1980 to May 2012), PsycINFO (1806 to May 2012), DARE(to May 2012), Cochrane CENTRAL and CINAHL (1981 to May 2012) using randomised controlled trial filters, where possible and free text terms for retention. C2-SPECTR (to May 2009) and ERIC (1966 to May 2009) were only searched to May 2009 because of difficulties encountered with database and search platform changes. PreMedline was searched to May 2009 but not subsequently because the free text records ultimately appear in MEDLINE. For search updates we also included the Cochrane Methodology Register, Current Controlled Trials metaRegister of Controlled Trials and WHO trials registry. Reference lists of relevant publications, reviews, included studies and abstracts of Society for Clinical Trials meetings from 1980-2012 were also reviewed. No language restrictions were applied. All UK clinical trial units were surveyed to identify further eligible trials and the review was advertised at the Society for Clinical Trials Meeting in 2010.

Trial selection

Two reviewers (VB, GR) independently screened potentially eligible trials with disagreements resolved by a third author (SS). Information was sought from investigators to clarify eligibility where this was unclear.

Data extraction

Data were extracted for each retention and host trial by one author (VB) and checked by another (JT). For retention trials, data were extracted on start time in relation to the host trial, aim, primary outcome, follow-up type, strategy to improve retention and comparator/s, including the frequency and time the strategy was administered, and numbers randomised, included and retained at the primary analysis. Data on sequence generation, allocation concealment, blinding and outcome reporting were extracted for each retention trial to assess risk of bias[9]. Data extracted for each host trial were: aim, comparators, primary outcome, disease area and setting. In addition, information on the sequence generation and allocation concealment was extracted to confirm that host trials were randomised. Missing or ambiguous data were queried or obtained through contact with trial authors.

Statistical analysis

Retention was the primary outcome. Most retention strategies were applied during follow-up for the host trial. For three host trials the retention strategy was applied in further follow-up of trial participants after completion. For four host trials the strategy was applied during the pilot phase and for one other host trial the retention strategy was applied before the host trial commenced. Where retention trials specified the primary outcome as the retention rate at a

For peer review only

For peer review only - http://bmjopen.bmj.com/site/about/guidelines.xhtml

particular time point, this was used in the analysis. Where trials reported retention at multiple time points, without specifying which one was the primary outcome, we used the earliest time point in the analysis to see the initial impact on retention or response of introducing the strategy. Where trials reported time to retention, without specifying the primary time point, we used the final time point in the analysis, taking account of any censoring if data were available.

Retention trials with insufficient data could not be included in meta-analyses and were described qualitatively. Otherwise, risk ratios and their 95% confidence intervals for retention were used to determine the effect of strategies on this outcome. The participant was the unit of analysis. Where clustering was ignored in the analysis of cluster randomised trials we inflated the standard errors using the intra-class correlation coefficients from appropriate external sources[10;11 12].

For factorial trials [13;14] that investigated different categories of strategies to improve retention, we included all trial comparisons in the relevant analyses and labelled these accordingly. For one factorial trial[15], where the data were not available to do this, only the broad trial comparisons (main effects) were included in the analyses. Where there were multiple comparisons in a single trial[16] within the same category of strategy, to avoid double counting, the intervention arms were combined and compared with the control arm. Similarly, for three-armed trials[17;18] that compared two similar intervention arms with one control arm, the intervention arms were combined and compared with the control arm. For these trials, we also compared each intervention arm with the control arm, as separate trial comparisons, in exploratory analyses. Note that these approaches resulted in more trial comparisons than trials.

Heterogeneity was examined by the chi^2 test, at 0.10 level of significance, and the I^2 statistic[19], and explored through subgroup analyses. If there was no substantial heterogeneity, risk ratios were pooled using the fixed effect model, but if heterogeneity was detected and was not explained by subgroup or sensitivity analyses, we did not pool results. If heterogeneity could not be explained we used the random effects model to assess the robustness of the results to the choice of model. To assess the robustness of the results, sensitivity analyses were conducted that excluded quasi-randomised trials.

The diversity of trials and interventions identified meant that not all of our pre-specified subgroup analyses were appropriate or possible. Therefore, different types of strategies were analysed separately and new subgroups were defined within these prior to analysis. These new analyses are listed in tables 1- 4.

For peer review only - http://bmjopen.bmj.com/site/about/guidelines.xhtml

Absolute benefits of effective retention strategies were based on applying meta-analysis risk ratios to representative control arm retention rates[20]. All statistical analyses were conducted using RevMan5.

Results

We identified 38 eligible randomised retention trials from 24,304 records (Fig 4). Twenty-eight of these were published in full[13-18;21-38], two in the grey literature[14;34] and eight are unpublished (*unpublished trials by Edwards, Svobodva, Letley, Maclennan, Land, Bailey 1, Bailey 2 Marson)*. Unpublished trials were identified by word of mouth, reference lists of relevant literature and a survey of UK clinical trials units. Four retention trial publications contained two trials each[18;32;33;35].

Participants and settings

Eligible retention trials were from different geographical areas and clinical settings. Clinical areas ranged from exercise and alcohol dependency to treatment and screening for cancer (Tables 1- 4)[12].

Outcomes for strategies to improve retention were measured by: return of postal or electronic questionnaires[13-15;18;21;22;24;25;27;29-34;36-41] (*unpublished trials by Edwards, Svobodva, Letley, Maclennan, Land, Bailey 1, Bailey 2 Marson)* or biomedical data [17] (*Bailey unpublished*) a combination of postal, telephone, and email follow-up[35] or face to face follow-up/retention[16;42].

Design of included retention trials

One retention trial was cluster randomised (*Land unpublished*), four were factorial trials[13-16] and there was one three-armed[17] and three four-armed trials[18;32]. Five trials were quasi randomised[16;29;33;42], allocating participants by either their identification numbers[29;42], day of clinic visit[16] or by random selection of half the sample for the intervention and half for the control group[33]. All strategies targeted individual trial participants except one which targeted sites (*Land unpublished*).

Twenty nine retention trials commenced during follow-up of the host trial[13;15;16;18;21;22;24-27;29-36;38;42;43] (*Edwards, Land, Maclennan, Bailey, Svoboda, unpublished*). One trial followed

children of mothers who participated in the MRC ORACLE trial[39]. Two trials followed up participants in smoking cessation trials after the host trial finished[17;40]. Another retention trial randomised participants before the host trial commenced[23]. Four trials commenced during the pilot phase of the host trial[18;32;37] (*Letley unpublished*). For one trial it is unclear when the retention trial commenced in relation to the host trial[14].

Incentive strategies

There were 14 retention trials of incentives and 19 trial comparisons. Thirteen trials investigating incentive strategies targeted questionnaire response, with only one targeting participant retention[16]. Incentive strategies aimed at improving questionnaire response were: vouchers[18;29;39], cash[25], a charity donation[18], entry into a prize draw[14;18;30], cheques[14;17] offers of study results[24;40] and a certificate of appreciation[15;16]. Incentive strategies aimed at participant retention were: lapel pins and a certificate of appreciation[16]. UK incentives ranged in value from £5-£20[18;29;39] (*Bailey unpublished*) and from $2-$10 for US based trials, and were provided as either cash or voucher. Offers of entry into prize draws ranged from £25- £250 for UK[18;30] and $US50 for US based trials[14] (Table 1), there was no information available on the chance of winning a prize. One trial evaluated giving a monetary incentive with a promise of a further incentive for return of trial data (*Bailey 2 unpublished*).

Communication strategies

There were 14 retention trials of communication strategies and 20 trial comparisons. Most communication strategies targeted questionnaire response, with only one targeted at the return of biomedical test kits[35]. Strategies evaluated were: enhanced letters i.e. those with additional information about trial processes or with an extra feature e.g. signed by a principal investigator[15;15] (*Marson unpublished*) use of additional telephone reminders[35] (*Maclennan unpublished*); a calendar including reminders of when to return a questionnaire[34]; text and/or email reminders[21;31;35] and reminders to sites of upcoming assessments versus no additional reminder (*Land unpublished*). One trial used a package of postal communication strategies called the Total Design Method (TDM)[37] and another used recorded delivery of questionnaires[38] (Table 2).

Five trials evaluated both communication and incentive strategies[13-15;25;35] (Tables 1 and 2). The incentives were: certificates of appreciation for study involvement[15], study branded pens[13], a US$2 coin[14] and a US$5 bill [25] or fridge magnets[35]. The communication strategies were: 1st or 2nd class outward post[13-15] stamped and business reply envelopes[13], letters

For peer review only - http://bmjopen.bmj.com/site/about/guidelines.xhtml

signed by different study personnel[15], letters posted at different times[15], telephone survey[25] and text messages[35].

New questionnaire formats

The effect of a change in questionnaire format on response to questionnaires was evaluated in eight trials. The 10 comparison formats evaluated were (Table 3): questionnaire length[27;32;36] (*Edwards unpublished Svoboda unpublished*) order of questions (Letley *unpublished*)[33] and relevance of questionnaires in the context of research in alcohol dependence [32].

Behavioural strategies

There were two retention trials of motivational behavioural strategies, one in an exercise trial[26] and another in a parenting trial[23] (Table 4). A behavioural strategy was defined as giving participants information about goal setting and time management to facilitate successful trial completion. One retention trial was run prior to the host trial[23], where only participants who completed the orientation/retention trial were included in the subsequent parenting trial.

Case management

Case management defined as outreach, service planning linkage, monitoring, and advocacy, was compared within usual follow-up in a cancer screening trial[28](Table 4). This strategy involved trial assistants managing participant follow-up by arranging services to enable participants to keep trial follow-up appointments.

Methodology strategies

One trial included an open trial versus blind trial design to evaluate the impact on questionnaire response[22] (Table 4).

Trials not included in the meta-analyses

Two included trials could not be included in the meta-analysis[30] (*Letley unpublished*). For one, the host trial participants included randomised and non-randomised participants[30] and the author confirmed that participants in the retention trial were from both cohorts and these data could not be separated. For the other, retention trial (*Letley unpublished*) outcome data were not available.

Risk of bias in included trials

For peer review only - http://bmjopen.bmj.com/site/about/guidelines.xhtml

Twenty four trials describe adequate sequence generation[15;16;18;22-24;26;30-32;34;35;37;39;40] (*unpublished trials Bailey2 Bailey1 Letley, Land, Maclennan, Marson*). There was insufficient information about the sequence generation for ten trials, but they were all described as randomised[13;14;17;21;25;27;36;38] (*Edwards, Svoboda unpublished*). Five trials used quasi randomisation[16;28;29;33]. Fifteen trials reported both adequate sequence generation and allocation concealment[18;22;24;26;31;32;34;39;40] (*Letley, Maclennan, Bailey [1,2,] unpublished*).

Blinding of participants to the intervention was not possible for incentive strategies offers of incentives, behavioural or case management strategies, and different types of communication and questionnaire format strategies and for one trial that evaluated the effect of a blind versus open design on retention this was not applicable[22]. For some trials, participants were aware of the intervention but unaware of the evaluation[14;16;23;30;33;39] (*Maclennan, Marson unpublished*). For another trial[26] exercise sessions were not separated according to the behavioural intervention i.e. walking and swimming, and potential contamination between groups could have led to bias. For other trials, blinding of participants or trial personnel to the outcome or intervention was not reported. The primary outcome measure for this review was retention, and this was well reported. Authors were contacted for clarification of any exclusions after randomisation if this was unclear from retention trial reports. Although retention trial protocols were not available for included trials, the published and unpublished reports included reported all expected outcomes for retention.

The effects of strategies

1. Incentive Strategies

There were 14 retention trials of incentives, 19 trial comparisons with 16,253 comparisons. Across incentive subgroups there was considerable heterogeneity (p<0.00001) Figure 1a. So we did not pool the results for incentives. Unless otherwise stated results from the random effects model were similar. Three trials (3166 participants) that evaluated the effect of giving monetary incentives to participants showed that the addition of monetary incentives is more effective than no incentive at increasing response to postal questionnaires (RR=1.18; 95% CI 1.09-1.28; p<0.0001, heterogeneity p=0.21 Figure 1a). A sensitivity analysis excluding the quasi randomised trial by Gates shows a similar effect (RR=1.31; 95% CI 1.11-1.55; p=0.002)[29]. Also, based on two web based trials (3613 participants, Figure 1a), an offer of a monetary incentive promotes greater return of electronic questionnaires than no offer (RR=1.25; 95% CI 1.14-1.38, p<0.00001, heterogeneity p=0.14). However, a single trial

For peer review only - http://bmjopen.bmj.com/site/about/guidelines.xhtml

comparison suggests that an offer of a monetary donation to charity does not increase response to electronic questionnaires (RR =1.02, 95% CI; 0.78-1.32; p=0.90 Figure 1a)

Based on three trials (6322 participants) there is no clear evidence that the addition of non-monetary incentives improved questionnaire response (RR=1.00; 95% CI 0.98-1.02; p=0.91) but there is some heterogeneity (p=0.02 Figure 1a). A sensitivity analysis excluding the quasi randomised trial by Bowen showed a similar effect (RR=1.00; 95% CI 0.93-1.08; p=0.99, heterogeneity p=0.01) [16]. Two trials (1,138 participants) evaluating offers of non-monetary incentives suggest that an offer of a non-monetary incentive is neither more nor less effective than no offer (RR=0.99; 95% CI 0.95-1.03; p=0.60; heterogeneity p=0.52) at improving questionnaire response Figure 1a.

In exploratory analyses, the different incentive arms that were combined for the main analysis do not appear to show differential effects (Figure 5).

Two trials (902 participants) show that higher value incentives are better at increasing response to postal questionnaires than lower value incentives (RR 1.12; 95% CI 1.04-1.22; p =0.005; heterogeneity p=0.39) irrespective of how they are given (Figure 1b).

Two trial comparisons (297 participants) provide no clear evidence that giving a monetary incentive is better than an offer of entry into a prize draw for improving response to postal questionnaires (RR=1.04; 95% CI 0.91- 1.19; p=0.56, heterogeneity p=0.18, Figure 1c).

One trial could not be included in the analysis[30], but showed a higher response in the group offered entry into a prize draw (70.5%) compared with the group not offered entry into the draw (65.8%).

2. Communication strategies

There were 14 trials of communication strategies and 20 comparisons with 9,822 participants. The communication strategies were so diverse that these were analysed separately.

Results from two trials (2479 participants) show that an enhanced letter is neither more nor less effective than a standard letter for increasing response to postal questionnaires (RR=1.01; 95% CI 0.97-1.05; p=0.70; heterogeneity p=0.80, Figure 2a) . Although based on a single trial (226 participants), the TDM package seems much more effective than a customary postal

For peer review only

communication method at increasing questionnaire return (RR=1.43, 95% CI 1.22-1.67; p<0.0001 Figure 2b). Based on the relevant arms of three trials (1888 participants), there is no clear evidence that priority post is either more or less effective than regular post at increasing trial questionnaire return (RR=1.02; 95% CI 0.95-1.09; p=0.55; heterogeneity p=0.53 Figure 2c).

Six trials (3401 participants) evaluated the effect of different types of reminders to participants on questionnaire response. There is no clear evidence that a reminder is either more or less effective than no reminder (RR=1.03; 95% CI 0.99-1.06; p=0.13; heterogeneity p=0.73) at improving trial questionnaire response (Figure 2d). One trial (700 participants) showed no clear evidence that a telephone survey is either more or less effective than a monetary incentive and a questionnaire for improving questionnaire response (RR=1.08; 95% CI 0.94-1.24; p=0.27, Fig 2e). Based on one cluster randomised trial (272 participants), a monthly reminder to sites of upcoming assessment was neither more nor less effective than the usual follow-up (RR=0.96; 95% CI 0.83-1.11; p=0.57). However, one small trial (192 participants) suggested that recorded delivery is more effective than a telephone reminder (RR= 2.08; 95% CI 1.11-3.87; p=0.02). Based on one other trial (664 participants), there is no clear evidence that sending questionnaires early increased or decreased response (RR=1.10; 95% CI 0.96-1.26; p=0.19).

3. New questionnaire strategies

Eight trials with ten comparisons (21,505 participants) evaluated the effect of a new questionnaire format on questionnaire response. Although there is only some heterogeneity between the questionnaire subgroups p=0.11 (Figure 3), it did not seem reasonable to pool the results based on such different interventions.

Five trials (7277 participants) compared the effect of short questionnaires versus long on postal questionnaire response. There is only a suggestion that short questionnaires may be better (RR=1.04; 95% CI 1.00-1.08; p=0.07, heterogeneity p=0.14, Figure 3). Based on one trial (900 participants), there is no clear evidence that long and clear questionnaires are more or less effective than shorter condensed questionnaires for increasing questionnaire response (RR= 1.01, 0.95-1.07; p=0.86, Figure 3). Two quasi randomised trials (9435 participants) also show no good evidence that placing disease/condition questions before generic questions is either more or less effective than vice versa at increasing questionnaire response (RR=1.00,

For peer review only

For peer review only - http://bmjopen.bmj.com/site/about/guidelines.xhtml

0.97-1.02; p=0.75, heterogeneity (p=0.44), Figure 3). One trial by Letley (*unpublished*) not included in this analysis, provided no estimate of effect.

In the context of research on reducing alcohol consumption there is also evidence that more relevant questionnaires i.e. those relating to alcohol use, increase response rates (RR 1.07; 95% CI 1.01-1.14; p= 0.03, Figure 3).

4. Behavioural / motivational strategies

Two community based trials (273 participants) show no clear evidence that the behavioural / motivational strategies used are either more or less effective than standard information for retaining participants (RR= 1.08; 95% CI 0.93-1.24; p=0.31 heterogeneity p=0.93)

5. Case management strategies

One trial (703 participants) evaluated the effect of intensive case management procedures on retention. There is no evidence that intensive case management is either more or less effective than usual follow-up in the population examined (RR=1.00; 95% CI 0.97-1.04; p=0.99)

6. Methodology strategies

One fracture prevention trial (538 participants) evaluated the effect of participants knowing their treatment allocation (open trial) compared to participants blind/unaware of their allocation on questionnaire response. The open design led to higher response rates (RR=1.37; 95% CI 1.16 -1.63; p=0.0003).

Absolute benefits of strategies to improve retention

The absolute benefits of effective strategies on typical questionnaire response are illustrated in Table 5. Based on a 40% baseline response rate for postal questionnaires, the addition of a monetary incentive is estimated to increase response by 92 questionnaires per 1000 sent (95% CI 50-131). With a baseline response rate of 30%, as seen in the included online trial, the addition of an offer of a monetary incentive is estimated to increase response by 140 questionnaires per 1000 (95% CI 86-193).

Discussion

Thirty-eight randomised retention trials were included in this review, evaluating six broad types of strategies to increase questionnaire response and retention in randomised trials. Trials

For peer review only - http://bmjopen.bmj.com/site/about/guidelines.xhtml

were conducted across a spectrum of disease areas, countries, health care, and community settings. Strategies with the clearest impact on questionnaire response were: addition of monetary incentives compared to no incentive for return of postal questionnaires, addition of an offer of a monetary incentive when compared to none for return of electronic questionnaires, and an offer of £20 vouchers when compared to £10 for return of postal questionnaires and biomedical test kits. The evidence was less clear about the effect of shorter questionnaires rather than longer questionnaires and for questionnaires of greater relevance to the questions being studied. Recorded delivery of questionnaires, the Total Design Method a "package" of postal communication strategies with reminder letters and an open trial design appear more effective than standard procedures. These strategies were tested in single trials and may need further evaluation. The addition of a non-monetary incentive or an offer of a non-monetary incentive compared to no incentive did not increase or decrease trial questionnaire response. "Enhanced" letters, letters delivered by priority post or additional reminders were also no more effective than standard communication. Altering questionnaire structure does not seem to increase response. No strategy had a clear impact on increasing the number of participants returning to sites for follow-up.

Strengths and weaknesses

This is the most comprehensive review of strategies specifically designed to improve retention in randomised trials, including many unpublished trials and data. Although our searches were extensive, some less well reported, on-going, or unpublished trials, or trials conducted outside the UK might have been missed.

Most trials used appropriate methods for randomisation or at least stated that they were randomised. For trials that did not describe their methods well or provide further information, there remains a potential risk of selection bias. Sensitivity analyses excluding quasi-randomised trials did not affect the results. In this context, where motivating participants to provide data or attend clinics is often the target of the interventions and so appropriately influences the outcome, lack of blinding is less of a concern. Retention is the outcome and was obtained for all but two trials so similarly, attrition and selective outcome reporting bias are probably unimportant. Although the retention trials were fairly well conducted, this could be improved, and they were often poorly reported. This may be because they were designed

For peer review only - http://bmjopen.bmj.com/site/about/guidelines.xhtml

when loss to follow-up became a problem in a trial, rather than pre planned prior to host trial commencement.

Few trials are available for behavioural, case management and methodological strategies (only one or two each) and this affects the power of the result for these strategies. The use of open trials to increase questionnaire response can only be applied to trials where blinding is not required, based on our result this strategy would need to be evaluated in different trial contexts if it were to be applied in other areas. All included studies were conducted in higher income countries. Therefore, the effective strategies may not be socially, culturally or economically appropriate to trials conducted in low resource settings. The diversity of strategies and the low number of trials meant that we could not examine the impact of, for example, trial setting and disease area as planned. Moreover, most of the evidence relates to increasing questionnaire response rather than participant retention in follow-up. Many trials require participants to return to sites for follow-up and monitoring; however barriers to follow-up do exist and are trial and participant specific depending on the disease area, treatment and population group. Return for follow-up at sites depends upon participant preferences and the demands of the trial.[44] Barriers to follow-up at site could be alleviated by using tailored strategies to encourage participants to return to sites for follow-up and monitoring. Studies that evaluate such strategies are particularly needed.

Edwards extensive review of methods to increase response to postal and electronic questionnaires found that monetary incentives and recorded delivery of questionnaires improved response[7]. However, unlike our review they also found that non-monetary incentives, shorter questionnaires, use of handwritten addresses, stamped return envelopes (as opposed to franked return envelopes) and, first class outward mailing were effective. We did however find that a "package" including an enhanced letter with several reminders was effective. The trials included in the Edwards review were embedded in surveys, cohort studies and trials and there was substantial heterogeneity in the results, which was not a particular problem in this review [7]. Moreover, we included seven unpublished trials and 18 other trials not included by Edwards[12].

Nakash's small systematic review of ways to increase response to postal questionnaires in health care was not exclusive to randomised trials[45]. They found reminder letters, telephone contact, and short questionnaires increased response to postal questionnaires. There was no

For peer review only

evidence that incentives were effective. A systematic review of methods to increase retention in population based cohort studies had no meta-analysis, but suggested that incentives were associated with increased retention[6].

Prior to our review, it was not clear which if any of these strategies could be extrapolated to randomised trials. We also identified additional strategies that may improve trial questionnaire response or retention for example, methodological strategies.

Implications

Although giving monetary incentives up front seems effective, offering and giving these after receipt of data could be a cost effective strategy, because those not returning questionnaires would not receive an incentive. The addition of non-monetary incentives for example, lapel pins and certificates of appreciation, or offers of these did not increase response or retention, perhaps because these items are not valued by participants. Offers of monetary incentives were also an effective strategy in the context of an online electronic questionnaire, thus it would be beneficial for trialists to know which is more effective: an offer of a monetary incentive or an upfront monetary incentive in a head to head trial comparison.

The value of incentives used in UK evaluations ranged from GBP5 to GBP20 and for US-based studies was USD2 to USD10. For offers of entries into prize draws, the values were higher, ranging from GBP25 to GBP250 for UK prize draws and USD50 for US-based prize draws. The value of monetary incentive should not be so high as to be perceived as payment or coercion for data but more as an appreciation for efforts made by participants. A cost effectiveness analysis for additional responses gained after incentive strategies were introduced was reported for only some incentive trials. As costs increase the cost benefit associated with incentive strategies would need to be updated if incentives were to be used to improve retention in future trials [25;29;39 18;30].

Priority post, enhanced letters (e.g. signed by the principal investigator) and different types of additional reminders are used by trialists in current research practice, but were not found to be effective. The former may not be considered important and too many reminders, over and above standard procedures, could be counterproductive.

For peer review only - http://bmjopen.bmj.com/site/about/guidelines.xhtml

Although appearing very effective, the total design method for postal questionnaires could be labour intensive to implement, expensive, and may no longer be applicable to some participant groups e.g. young people used to other modes of communication, or in trials using email, text or online data collection. Recorded delivery could be useful to ensure trial follow-up supplies reach their intended destination, but careful planning to avoid inconvenience for the participant might be necessary. Open trials to increase questionnaire response can only be used where blinding is not required. This could be counterproductive, however, as unblinded trials can cause biased outcome assessment or loss to follow-up if a participant or clinician has a treatment preference.

Questionnaire length and relevance may need further evaluation as there is only a suggestion that these are effective in the context of randomised trials. Also, telephone follow-up compared with a monetary incentive sent with a questionnaire needs further evaluation possibly with a cost benefit analysis as both could be expensive in time and human resources. Evaluations of strategies that encourage participants to return to sites for follow-up visits and monitoring are particularly needed because many trials collect outcome data in this way.

Trialists should consider including well thought out and adequately powered evaluations of strategies to increase retention in randomised trials with a clear definition of retention strategies and retention measures. Trialists could incorporate evaluations of strategies to improve retention at the design stage so that power, sample size and funding are taken into account. Retention trials were often poorly reported and trialists should adhere to the consort guidelines for trial reporting to facilitate the synthesis of results in future methodology reviews.

There is less research on ways to increase return of participants to trial sites for follow-up and on the effectiveness of strategies to retain trial sites in cluster and individual randomised trials. Research in both areas would be very beneficial to trialists. Application of the results of this review would depend on trial setting, population, disease area, budget allowance and follow-up procedures.

Conclusions

For peer review only

For peer review only - http://bmjopen.bmj.com/site/about/guidelines.xhtml

Trialists should consider using monetary incentives and offers of monetary incentives to increase postal and electronic questionnaire response, depending on trial setting, population, disease area, budget, and usual follow-up procedures.

Future evaluations of retention strategies in randomised trials should be carefully planned and adequately powered, and the retention strategies and measures of retention clearly defined. More research on ways to increase return of participants to sites for follow-up, and on ways to retain sites in cluster and individual randomised trials are also needed.

Acknowledgements

We would like to thank the following: all authors of included published trials for providing extra unreported data; principal investigators for data on trials in progress or completed and unpublished (Julia Bailey UCL for data for Bailey 1 and Bailey 2; Graeme MacLennan for data for MacLennan; Stephanie Land data for Land) and the coordinators of UK Clinical Trials Units who responded to our survey with information about on going and/or unpublished completed trials. We also thank Cara Booker, SPHRU for search strategy information; Angela Young, Librarian UCL for assistance with searching databases and Ian White MRC Bio Statistics Unit Cambridge for helpful comments on the analysis of multi arm trials. We also acknowledge Shaun Treweek, Mike Clarke, Andy Oxman, Karen Robinson and Anne Eisinga for comments on the review protocol; and Phil Edwards, Elie Akl, Lisa Maguire, Jean Suvan, Shaun Treweek, Mike Clarke and Karen Robinson for comments on the review. This project was funded by the MRC Population Health Sciences Research Network (Grant Number PHSRN 30).

Statement of funding and competing interests

All authors have completed the ICMJE uniform disclosure form and declare no competing interests. This project was funded by the Medical Research Council Population Health Sciences Research Network grant number PHSRN 30.

Contributorship Statement

VB wrote the protocol for the review with comments from JT, GR, SS, SM, IN, SH. JT and VB designed the searches with comments from SH. VB conducted the searches, screened all abstracts, and full papers of potentially eligible trials. VB and GR screened potentially

For peer review only - http://bmjopen.bmj.com/site/about/guidelines.xhtml

eligible trial papers. SS acted as a third reviewer. Data extraction was conducted by VB and checked by JT. JT designed the analysis plan with VB. VB conducted the analysis with advice on interpretation of results from JT, SS, IN, GR. VB wrote the first draft of the review with critical comments from all authors.

Competing Interests

None

Data Sharing Statement

There is no additional data available

For peer review only

Reference List

(1) Fewtrell MS, Kennedy K, Singhal A, Martin RM, Ness A, Hadders-Algra M et al. How much loss to follow-up is acceptable in long-term randomised trials and prospective studies? *Arch Dis Child* 2008; 93(6):458-461.

(2) Schulz KF, Grimes DA. Sample size slippages in randomised trials: exclusions and the lost and wayward. *The Lancet* 2002; 359(9308):781-785.

(3) Hollis S, Campbell F. What is meant by intention to treat analysis? Survey of published randomised controlled trials. *BMJ* 1999; 319:670-674.

(4) Robinson KA, Dennison CR, Wayman DM, Pronovost PJ, Needham DM. Systematic review identifies number of strategies important for retaining study participants. *J Clin Epidemiol* 2007; 60(8):757.

(5) Davis L, Broome M, Cox R. Maximizing Retention in Community-based Clinical Trials. *Journal of Nursing Scholarship* 2002; 34(1):47-53.

(6) Booker C, Harding S, Benzeval M. A systematic review of the effect of retention methods in population-based cohort studies. *BMC Public Health* 2011; 11(1):249.

(7) Edwards PJ, Roberts IG, Clarke MJ, DiGuiseppi C, Wentz R, Kwan I et al. Methods to increase response rates to postal and electronic questionnaires. *Cochrane Database of Systematic Reviews 2009, Issue 3 Art No : MR000008* 2009;(3).

(8) Brueton VC, Rait G, Tierney J, Meredith S, Darbyshire J, Harding S et al. Strategies to reduce attrition in randomised trials. *Cochrane Database of Systematic Reviews Art No :MR000032 DOI: 10 1002/14651858 MR000032* 2011;(2).

(9) Higgins J, Altman D. Assessing risk of bias in included studies. In: Higgins J, Sally Green, editors. Cochrane Hanbook for Systematic Reviews of Interventions. John Wiley; 2008. 187-242.

(10) Higgins J, Deeks.J, Altman D. Special topics in statistics. In: Julian PT Higgins, Sally Green, editors. Cochrane Handbook for Systematic Reviews of Intervenions. John Wiley; 2008. 482-529.

(11) University of Aberdeen. Aberdeen ICCs. 2013.

Ref Type: Online Source

For peer review only

(12) Brueton VC, Tierney J, Stenning S, Nazareth I, Meredith S, Harding S et al. Strategies to improve retention in randomised trials . *Cochrane Methodology Group* 2013; in press.

(13) Sharp L, Cochran C, Cotton SC, Gray NM, Gallagher ME. Enclosing a pen with a postal questionnaire can significantly increase the response rate. *J Clin Epidemiol* 2006; 59(7):747-754.

(14) Kenton L, Dennis CL, Weston J, Kiss A. Abstracts from the 28th Meeting of the Society of Clinical Trials, Montreal, May 20–23, 2007:The effect of incentives and high priority mailing on postal questionnaire response rates: A Mini-RCT. Clinical Trials 4[4], 371-455. 1-8-2007.

Ref Type: Abstract

(15) Renfroe EG, Heywood G, Foreman L, Schron E, Powell J, Baessler C et al. The end-of-study patient survey: methods influencing response rate in the AVID Trial. *Control Clin Trials* 2002; 23(5):521-533.

(16) Bowen D, Thornquist M, Goodman G, Omenn GS, Anderson K, Barnett M et al. Effects of Incentive Items on Participation in a Randomized Chemoprevention Trial. *J Health Psychol* 2000; 5(1):109-115.

(17) Bauer JE, Rezaishiraz H, Head K, Cowell J, Bepler G, Aiken M et al. Obtaining DNA from a geographically dispersed cohort of current and former smokers: Use of mail-based mouthwash collection and monetary incentives. *Nicotine & Tobacco Research* 2004; 6(3):439-446.

(18) Khadjesari Z, Murray E, Kalaitzaki E, White I, Mc Cambridge J, Thompson S et al. Impact and costs of incentives to reduce attrition in online trials:Two randomised controlled trials. *Journal of Medical Internet Research* 2011; 13(1):e26.

(19) Higgins JPT, Thompson SG, Deeks JJ, Altman DG. Measuring inconsistency in meta-analyses. *BMJ* 2003; 327(7414):557-560.

(20) Schunemann H, Oxman AD, Visr G, Higgins J, Deeks D, Glasziou P et al. Interpreting results and drawing conclusions. In: Higgens J, Green S, editors. Cochrane Handbook for Systematic Reviews of Interventions. Chichester West Sussex: John Wiley and Sons Ltd; 2008. 359-387.

(21) Ashby R, Turner G, Cross B, Mitchell N, Torgerson D. A randomized trial of electronic reminders showed a reduction in the time to respond to postal questionnaires. *J Clin Epidemiol* 2011; 64(2):208-212.

(22) Avenell A, Grant AM, McGee M, McPherson G, Campbell MK, McGee MA et al. The effects of an open design on trial participant recruitment, compliance and retention - a randomized controlled trial comparison with a blinded, placebo-controlled design. *Clinical Trials* 2004; 1(6):490-498.

For peer review only

(23) Chaffin M, Valle LA, Funderburk B, Gurwitch R, Silovsky J, Bard D et al. A Motivational Intervention Can Improve Retention in PCIT for Low-Motivation Child Welfare Clients. *Child Maltreatment* 2009; 14(4):356-368.

(24) Cockayne S, Torgerson D. A randomised controlled trial to assess the effectiveness of offering study results as an incentive to increase response rates to postal questionnaires [ISRCTN26118436]. *BMC Medical Research Methodology* 2005; 5(1):34.

(25) Couper PM, Peytchev A, Strecher JV, Rothert K, Anderson J. Following Up Nonrespondents to an Online Weight Management Intervention: Randomized Trial Comparing Mail versus Telephone. *J Med Internet Res* 2007; 9(2):e16.

(26) Cox KL, Burke V, Beilin LJ, Derbyshire AJ, Grove JR, Blanksby BA et al. Short and long-term adherence to swimming and walking programs in older women -- The Sedentary Women Exercise Adherence Trial (SWEAT 2). *Prev Med* 2008; 46(6):511-517.

(27) Dorman P, Slattery J, Farrell B, Dennis MS, Sandercock PA. A randomised comparison of the EuroQol and Short Form-36 after stroke. United Kingdom collaborators in the International Stroke Trial. *BMJ* 1997; 315(7106):461.

(28) Ford ME, Havstad S, Vernon SW, Davis SD, Kroll D, Lamerato L et al. Enhancing Adherence Among Older African American Men Enrolled in a Longitudinal Cancer Screening Trial. *Gerontologist* 2006; 46(4):545-550.

(29) Gates S, Williams M, Withers E, Williamson E, Mt-Isa S, Lamb S. Does a monetary incentive improve the response to a postal questionnaire in a randomised controlled trial? The MINT incentive study. *Trials* 2009; 10(1):44.

(30) Leigh Brown AP, Lawrie H, Kennedy A, Webb A, Torgerson D, Grant A. Cost effectiveness of a prize draw on response to a postal questionnaire:results of a randomised trial among orthopaedic outpatients in Edinburgh. *Journal of Epidemiology and Public Health* 1997; 51:463-464.

(31) Man MS, Tilbrook HE, Jayakody S, Hewitt CE, Cox H, Cross B et al. Electronic reminders did not improve postal questionnaire response rates or response times: a randomized controlled trial. *J Clin Epidemiol* 2011; 64(9):1001-1004.

(32) McCambridge J, Kalaitzaki E, White RI, Khadjesari Z, Murray E, Linke S et al. Impact of Length or Relevance of Questionnaires on Attrition in Online Trials: Randomized Controlled Trial. *J Med Internet Res* 2011; 13(4):e96.

(33) McColl EM, Eccles MPM, Rousseau NSB, Steen INP, Parkin DWD, Grimshaw JMP. From the Generic to the Condition-specific?: Instrument Order Effects in Quality of Life Assessment. [Article]. *Med Care* 2003; 41(7):777-790.

For peer review only - http://bmjopen.bmj.com/site/about/guidelines.xhtml

(34) Nakash R. A study of response and non-response to postal questionnaire follow-up in clinical trials. Chapter 6: A radomised controlled trial of a method of improving response to postal questionnaire follow-up in a clinical trial. [University of Warwick; 2007.

(35) Severi E, Free C, Knight R, Robertson S, Edwards P, Hoile E. Two controlled trials to increase participant retention in a randomized controlled trial of mobile phone-based smoking cessation support in the United Kingdom. *Clinical Trials* 2011; 8(5):654-660.

(36) Subar AF, Ziegler RG, Thompson FE, Johnson CC, Weissfeld JL, Reding D et al. Is Shorter Always Better? Relative Importance of Questionnaire Length and Cognitive Ease on Response Rates and Data Quality for Two Dietary Questionnaires. *Am J Epidemiol* 2001; 153(4):404-409.

(37) Sutherland HJ, Beaton M, Mazer R, Kriukov V, Boyd NF. A randomized trial of the total design method for the postal follow-up of women in a cancer prevention trial. *Eur J Cancer Prev* 1996; 5(3):165-168.

(38) Tai SS, Nazareth I, Haines A, Jowett C. A randomized trial of the impact of telephone and recorded delivery reminders on the response rate to research questionnaires. *J Public Health* 1997; 19(2):219-221.

(39) Kenyon S, Pike K, Jones D, Taylor D, Salt A, Marlow N et al. The effect of a monetary incentive on return of a postal health and development questionnaire: a randomised trial [ISRCTN53994660]. *BMC Health Services Research* 2005; 5(1):55.

(40) JR Hughes. Free reprints to increase the return of follow-up questionnaires. Controlled Clinical Trials . 1989.

Ref Type: Abstract

(41) The International Stroke Trial (IST): a randomised trial of aspirin, subcutaneous heparin, both, or neither among 19[punctuation space]435 patients with acute ischaemic stroke. *The Lancet* 1997; 349(9065):1569-1581.

(42) Ford ME, Havstad S, Vernon SW, Davis SD, Kroll D, Lamerato L et al. Enhancing Adherence Among Older African American Men Enrolled in a Longitudinal Cancer Screening Trial. *Gerontologist* 2006; 46(4):545-550.

(43) Marson A, Appleton R, BakerG, Chadwick D, Doughty J, Eaton B et al. A randomised controlled trial examining the longer-term outcomes of standard versus new antiepileptic drugs The SANAD trial. *NIHR HTA Report* 2007; 11(37).

(44) Ross S, Grant A, Counsell C, Gillespie W, Russell I, Prescott R. Barriers to Participation in Randomised Controlled Trials: A Systematic Review. *J Clin Epidemiol* 1999; 52(12):1143-1156.

For peer review only

For peer review only - http://bmjopen.bmj.com/site/about/guidelines.xhtml

(45) Nakash R, Hutton J, Jorstad-Stein E, Gates S, Lamb S. Maximising response to postal questionnaires - A systematic review of randomised trials in health research. *BMC Medical Research Methodology* 2006; 6(1):5.

(46) Boyd N, Cousins M, Lockwood G, Tritchler D. Dietary fat and breast cancer risk: The feasibility of a clinical trial of breast cancer prevention. *Lipids* 1992; 27(10):821-826.

(47) Buys S, Partridge E, Greene M, Prorok P, Reding D, Riley T et al. Ovarian cancer screening in the Prostate, Lung, Colorectal and Ovarian (PLCO) cancer screening trial: Findings from the initial screen of a randomized trial. *Am J Obstet Gynecol* 2005; 193(5):1630-1639.

(48) Cooke MW, Marsh JL, Clarke M, Nakash R, Jarvis RM, Hutton JL et al. Treatment of severe ankle sprain: a pragmatic randomised controlled trial comparing the clinical effectiveness and cost effectiveness of three types of mechanical ankle support with tubular bandage. The CAST trial. 13, 1-144. 2009. NIHR Health Technology Assessment Programme.

Ref Type: Report

(49) CRASH trial collaborators. Effect of intravenous corticosteroids on death within 14 days in 10[punctuation space]008 adults with clinically significant head injury (MRC CRASH trial): randomised placebo-controlled trial. *The Lancet* 2004; 364(9442):1321-1328.

(50) Dennis CL, Hodnett E, Kenton L, Weston J, Zupancic J, Stewart DE et al. Effect of peer support on prevention of postnatal depression among high risk women: multisite randomised controlled trial. *BMJ* 2009; 338(jan15_2):a3064.

(51) Eccles M, McColl E, Steen N, Rousseau N, Grimshaw J, Parkin D et al. Effect of computerised evidence based guidelines on management of asthma and angina in adults in primary care: cluster randomised controlled trial. *BMJ* 2002; 325(7370):941.

(52) Free C, Knight R, Robertson S, Whittaker R, Edwards P, Zhou W et al. Smoking cessation support delivered via mobile phone text messaging (txt2stop): a single-blind, randomised trial. *The Lancet* 2011; 378(9785):49-55.

(53) Gail MH, Byar DP, Pechacek TF, Corle DK. Aspects of statistical design for the community intervention trial for smoking cessation (COMMIT). *Control Clin Trials* 1992; 13(1):6-21.

(54) Hughes JR, Hatsukami D, Pickens R, Krahn D, Malin S, Luknic A. Effect of nicotine on the tobacco withdrawal syndrome. *Psychopharmacology (Berl)* 1984; 83(1):82-87.

(55) Kenyon SL, Taylor DJ, Tarnow-Mordi W. Broad-spectrum antibiotics for preterm, prelabour rupture of fetal membranes: the ORACLE I randomised trial. *The Lancet* 2001; 357(9261):979-988.

For peer review only

(56) Kenyon SL, Taylor DJ, Tarnow-Mordi W. Broad-spectrum antibiotics for spontaneous preterm labour: the ORACLE II randomised trial. *The Lancet* 2001; 357(9261):989-994.

(57) Leigh Brown A, Kennedy A, Torgerson D, Campbell J, Webb J, Grant A. The OMENS trial: opportunistic evaluation of musculo-skeletal physician care among orthopaedic outpatients unlikely to require surgery. *Health Bull (Edinb)* 2001; 59(3):198-210.

(58) Marson AG, Al Kharusi AM, Alwaidh M, Appleton R, Baker GA, Chadwick DW et al. The SANAD study of effectiveness of valproate, lamotrigine, or topiramate for generalised and unclassifiable epilepsy: an unblinded randomised controlled trial. *The Lancet* 2007; 369(9566):1016-1026.

(59) Lamb S, Gates S, Underwood M, Cooke M, Ashby D, Szczepura A et al. Managing Injuries of the Neck Trial (MINT): design of a randomised controlled trial of treatments for whiplash associated disorders. *BMC Musculoskeletal Disorders* 2007; 8(1):7.

(60) Murray E, McCambridge J, Khadjesari Z, White I, Thompson S, Godfrey C et al. The DYD-RCT protocol: an on-line randomised controlled trial of an interactive computer-based intervention compared with a standard information website to reduce alcohol consumption among hazardous drinkers. *BMC Public Health* 2007; 7(1):306.

(61) Omenn GS, Goodman GE, Thornquist MD, Balmes J, Cullen MR, Glass A et al. Effects of a Combination of Beta Carotene and Vitamin A on Lung Cancer and Cardiovascular Disease. *N Engl J Med* 1996; 334(18):1150-1155.

(62) Porthouse J, Sarah C, Christine K, Lucy S, Elizabeth S, Terry A et al. Randomised controlled trial of calcium and supplementation with cholecalciferol (vitamin D3) for prevention of fractures in primary care. *BMJ* 2005; 330.

(63) The RECORD Trial Group. Oral vitamin D3 and calcium for secondary prevention of low-trauma fractures in elderly people (Randomised Evaluation of Calcium Or vitamin D, RECORD): a randomised placebo-controlled trial. *The Lancet* 2007; 365(9471):1621-1628.

(64) Tai S, Nazareth I, Donegan C, Haines A. Evaluation of General Practice Computer Templates. *Methods Inf Med* 1999; 38:177-181.

(65) The Antiarrhythmics versus Implantable Defibrillators (AVID) Investigators. A Comparison of Antiarrhythmic-Drug Therapy with Implantable Defibrillators in Patients Resuscitated from Near-Fatal Ventricular Arrhythmias. *N Engl J Med* 1997; 337(22):1576-1584.

(66) Tilbrook HE, Cox H, Hewitt CE, Kang'ombe AR, Chuang LH, Jayakody S et al. Yoga for Chronic Low Back PainA Randomized Trial. *Ann Intern Med* 2011; 155(9):569-578.

For peer review only - http://bmjopen.bmj.com/site/about/guidelines.xhtml

(67) Rothert K, Strecher VJ, Doyle LA, Caplan WM, Joyce JS, Jimison HB et al. Web-based Weight Management Programs in an Integrated Health Care Setting: A Randomized, Controlled Trial[ast]. *Obesity* 2006; 14(2):266-272.

(68) TOMBOLA Group. Cytological surveillance compared with immediate referral for colposcopy in management of women with low grade cervical abnormalities: multicentre randomised controlled trial. *BMJ* 2009; 339(jul28_2):b2546.

(69) TOMBOLA Group. Biopsy and selective recall compared with immediate large loop excision in management of women with low grade abnormal cervical cytology referred for colposcopy: multicentre randomised controlled trial. *BMJ* 2009; 339(jul28_2):b2548.

(70) UK BEAM Trial Team. United Kingdom back pain exercise and manipulation (UK BEAM) randomised trial: effectiveness of physical treatments for back pain in primary care. *BMJ* 2004;bmj.

For peer review only

Reference list of host trials within which retention trials were embedded [23;26;41;46-70].

For peer review only - http://bmjopen.bmj.com/site/about/guidelines.xhtml

Title page

Strategies to improve retention in randomised trials: a Cochrane systematic review and meta-analysis[1]

Brueton VC

Research Scientist, Meta-analysis Group
MRC Clinical Trials Unit at UCL
Aviation House
125 Kingsway
London WC2B 6NH
e-mail: vcb@ctu.mrc.ac.uk

Tierney JF

Head of Meta-analysis Group
MRC Clinical Trials Unit at UCL
Aviation House
125 Kingsway
London WC2B 6NH

Stenning S

Senior Statistician
MRC Clinical Trials Unit at UCL
Aviation House
125 Kingsway
London WC2B 6NH

Meredith S

Deputy Director
MRC Clinical Trials Unit at UCL
Aviation House
125 Kingsway
London WC2B 6NH

Harding S

Program leader: Ethnicity and Health
MRC Social & Public Health Sciences Unit
4 Lilybank Gardens
Glasgow G12 8RZ

[1] This article is based on a Cochrane Review to be published in the Cochrane Database of Systematic Reviews (CDSR) 2013, Issue X, DOI:10.1002/14651858.CD00xxxx (see www.thecochranelibrary.com for information)

For peer review only

BMJ Open REVIEW submitted 07.08.2013

Nazareth I

Professor of Primary Care
PRIMENT Clinical Trials Unit
Research Department of Primary care and Population Health
UCL Medical School
Rowland Hill Street
London
NW3 2PF

Rait G

Clinical Senior Lecturer
PRIMENT Clinical Trials Unit
Research Department of Primary care and Population Health
UCL Medical School
Rowland Hill Street
London
NW3 2PF

For peer review only

For peer review only - http://bmjopen.bmj.com/site/about/guidelines.xhtml

BMJ Open REVIEW submitted 07.08.2013

Objective

To quantify the effect of strategies to improve retention in randomised trials.

Design

Systematic review and meta-analysis.

Data sources

Sources searched: MEDLINE, EMBASE, PsycINFO, DARE, CENTRAL, CINAHL, C2-SPECTR, ERIC, PreMEDLINE, Cochrane Methodology Register, Current Controlled Trials metaRegister, WHO trials platform, Society for Clinical Trials (SCT) conference proceedings, and a survey of all UK clinical trial research units.

Review methods

Included trials were randomised evaluations of strategies to improve retention embedded within host randomised trials. The primary outcome was retention of trial participants. Data from trials were pooled using the fixed-effect model. Subgroup analyses were used to explore heterogeneity and to determine whether there were any differences in effect by type of strategy.

Results

38 retention trials were identified. Six broad types of strategies were evaluated. Strategies that increased postal questionnaire responses were: adding i.e. giving a monetary incentive (RR 1.18; 95% CI 1.09-1.28) and higher valued incentives (RR 1.12; 95% CI 1.04-1.22). Offering a monetary incentive i.e. incentive given on receipt of a completed questionnaire, also increased electronic questionnaire response (RR 1.25; 95% CI 1.14-1.38). The evidence for shorter questionnaires (RR 1.04; 95% CI 1.00-1.08) and questionnaires relevant to the disease/condition (RR 1.07; 95% CI 1.01-1.14) is less clear.

Based on the results of single trials the following strategies appeared effective at increasing questionnaire response: recorded delivery of questionnaires (RR 2.08; 95% CI 1.11-3.87); a "package" of postal communication strategies (RR 1.43; 95% CI 1.22-1.67), and an open trial design (RR 1.37; 95% CI 1.16 -1.63). There is no good evidence that the following strategies impact on trial response/retention: adding a non-monetary incentive (RR=1.00; 95% CI 0.98-1.02); offering a non-monetary incentive (RR=0.99; 95% CI 0.95-1.03); "enhanced" letters (RR=1.01; 95% CI 0.97-1.05); monetary incentives compared to offering prize draw entry (RR=1.04; 95% CI 0.91- 1.19); priority postal delivery (RR=1.02; 95% CI 0.95 - 1.09); behavioural motivational strategies (RR= 1.08; 95% CI 0.93-1.24); additional reminders to

For peer review only

For peer review only - http://bmjopen.bmj.com/site/about/guidelines.xhtml

participants (RR=1.03; 95% CI 0.99-1.06); and questionnaire question order (RR=1.00, 0.97-1.02).

Also based on single trials, these strategies do not appear effective: a telephone survey compared to a monetary incentive plus questionnaire (RR=1.08; 95% CI 0.94-1.24); offering a charity donation (RR =1.02, 95% CI; 0.78-1.32); sending sites reminders (RR= 0.96; 95% CI 0.83-1.11); sending questionnaires early (RR=1.10; 95% CI 0.96-1.26); longer and clearer questionnaires (RR= 1.01, 0.95-1.07) and participant case management by trial assistants (RR=1.00; 95% CI 0.97-1.04).

Conclusion

Most trials evaluated questionnaire response rather than ways to improve participants return to site for follow-up. Monetary incentives and offers of monetary incentives increase postal and electronic questionnaire response. Some strategies need further evaluation. Application of these results would depend on trial context and follow-up procedures.

For peer review only - http://bmjopen.bmj.com/site/about/guidelines.xhtml

BMJ Open REVIEW submitted 07.08.2013

Introduction

Loss of participants during study follow-up can introduce bias and reduce power affecting the generalisability, validity, and reliability of results[1;2]~~[1,2]~~ . If losses are fewer than 5% they may lead to minimum bias, while 20% loss can threaten trial validity[2]~~[2]~~. While missing data from losses to follow-up can be dealt with statistically, the risk of bias can remain[3]~~[3]~~.

Trialists adopt various strategies to try to improve retention and generate maximum data return or compliance to follow-up procedures. These strategies are designed to motivate and keep participants or site clinicians engaged in a trial, but many are untested[4;5]~~[4,5]~~. A systematic review of strategies to retain participants cohort studies suggests that providing incentives can improve retention[6]~~[6]~~. Edwards systematic review on methods to increase response rates to postal and electronic questionnaires across a range of study types found that including monetary incentives, keeping the questionnaire short and contacting people before questionnaires were sent were ways to increase response rates[7]~~[7]~~. However, heterogeneity of effects was an issue and it is unclear which strategies are applicable to randomised trials. Moreover, reasons for loss to follow-up in cohort studies and surveys may differ from randomised trials. In trials, participants may be randomised to a study arm that is not their preferred choice and so strategies that improve retention in other study types cannot necessarily be extrapolated to randomised trials.

As loss to follow-up can compromise the validity of findings from randomised trials, delay results and potentially increase trial costs, we conducted a systematic review to assess the effect of strategies to improve retention in randomised trials.

Methods

The methods were pre-specified in the Cochrane review protocol[8]~~[8]~~.

Trials included

We included randomised trials that compared strategies to increase participant retention embedded in "host" randomised trials across disease areas and settings. These strategies should have been designed for use after participants were recruited and randomised. Retention trials embedded in cohort studies and surveys were excluded.

Identification of retention trials

We searched MEDLINE (1950 to May 2012), EMBASE (1980 to May 2012), PsycINFO (1806 to May 2012), DARE(to May 2012), Cochrane CENTRAL and CINAHL (1981 to May

Formatted: Do not check spelling or grammar, Superscript

Formatted: Do not check spelling or grammar, Superscript

Formatted: Do not check spelling or grammar, Superscript

Formatted: Do not check spelling or grammar, Superscript

Formatted: Do not check spelling or grammar, Superscript

Formatted: Do not check spelling or grammar, Superscript

Formatted: Do not check spelling or grammar, Superscript

Formatted: Font: (Default) Times New Roman, 12 pt, Font color: Auto

Formatted: Font: (Default) Times New Roman, 12 pt, Font color: Auto

Formatted: Font: 12 pt, Font color: Auto

Formatted: Font: (Default) Times New Roman, 12 pt, Font color: Auto

Formatted: Font: (Default) Times New Roman, 12 pt, Font color: Auto

For peer review only - http://bmjopen.bmj.com/site/about/guidelines.xhtml

2012) using randomised controlled trial filters, where possible and free text terms for retention. C2-SPECTR (to May 2009) and ERIC (1966 to May 2009) were only searched to May 2009 because of difficulties encountered with database and search platform changes. PreMedline was searched to May 2009 but not subsequently because the free text records ultimately appear in MEDLINE. For search updates we also included the Cochrane Methodology Register, Current Controlled Trials metaRegister of Controlled Trials and WHO trials registry. Reference lists of relevant publications, reviews, included studies and abstracts of Society for Clinical Trials meetings from 1980-2012 were also reviewed. No language restrictions were applied. All UK clinical trial units were surveyed to identify further eligible trials and the review was advertised at the Society for Clinical Trials Meeting in 2010.

Formatted: Font: 12 pt, Font color: Auto

Trial selection

Two reviewers (VB, GR) independently screened potentially eligible trials with disagreements resolved by a third author (SS). Information was sought from investigators to clarify eligibility where this was unclear.

Data extraction

Data were extracted for each retention and host trial by one author (VB) and checked by another (JT). For retention trials, data were extracted on start time in relation to the host trial, aim, primary outcome, follow-up type, strategy to improve retention and comparator/s, including the frequency and time the strategy was administered, and numbers randomised, included and retained at the primary analysis. Data on sequence generation, allocation concealment, blinding and outcome reporting were extracted for each retention trial to assess risk of bias[99]. Data extracted for each host trial were: aim, comparators, primary outcome, disease area and setting. In addition, information on the sequence generation and allocation concealment was extracted to confirm that host trials were randomised. Missing or ambiguous data were queried or obtained through contact with trial authors.

Formatted: Do not check spelling or grammar, Superscript

Statistical analysis

Retention was the primary outcome. Most retention strategies were applied during follow-up for the host trial. For three host trials the retention strategy was applied in further follow-up of trial participants after completion. For four host trials the strategy was applied during the pilot phase and for one other host trial the retention strategy was applied before the host trial commenced. Where retention trials specified the primary outcome as the retention rate at a particular time point, this was used in the analysis. Where trials reported retention at multiple time points, without specifying which one was the primary outcome, we used the earliest time point in the analysis to see the initial impact on retention or response of introducing the

Formatted: Normal, Space Before: Auto, After: Auto, Line spacing: At least 18 pt

For peer review only - http://bmjopen.bmj.com/site/about/guidelines.xhtml

BMJ Open REVIEW submitted 07.08.2013

strategy. Where trials reported time to retention, without specifying the primary time point, we used the final time point in the analysis, taking account of any censoring if data were available.

Formatted: Font color: Blue

Retention trials with insufficient data could not be included in meta-analyses and were described qualitatively. Otherwise, risk ratios and their 95% confidence intervals for retention were used to determine the effect of strategies on this outcome. The participant was the unit of analysis. Where clustering was ignored in the analysis of cluster randomised trials we inflated the standard errors using the intra-class correlation coefficients from appropriate external sources[10;11][10,11] [12][12].

Formatted: Do not check spelling or grammar, Superscript

Formatted: Do not check spelling or grammar, Superscript

For factorial trials [13;14][13,14] that investigated different categories of strategies to improve retention, we included all trial comparisons in the relevant analyses and labelled these accordingly. For one factorial trial[15][15], where the data were not available to do this, only the broad trial comparisons (main effects) were included in the analyses. Where there were multiple comparisons in a single trial[16][16] within the same category of strategy, to avoid double counting, the intervention arms were combined and compared with the control arm. Similarly, for three-armed trials[17;18][17,18] that compared two similar intervention arms with one control arm, the intervention arms were combined and compared with the control arm. For these trials, we also compared each intervention arm with the control arm, as separate trial comparisons, in exploratory analyses. Note that these approaches resulted in more trial comparisons than trials.

Formatted: Do not check spelling or grammar, Superscript

Formatted: Do not check spelling or grammar, Superscript

Formatted: Do not check spelling or grammar, Superscript

Formatted: Do not check spelling or grammar, Superscript

Heterogeneity was examined by the chi^2 test, at 0.10 level of significance, and the I^2 statistic[19][19], and explored through subgroup analyses. If there was no substantial heterogeneity, risk ratios were pooled using the fixed effect model, but if heterogeneity was detected and was not explained by subgroup or sensitivity analyses, we did not pool results. If heterogeneity could not be explained we used the random effects model to assess the robustness of the results to the choice of model. To assess the robustness of the results, sensitivity analyses were conducted that excluded quasi-randomised trials.

Formatted: Do not check spelling or grammar, Superscript

The diversity of trials and interventions identified meant that not all of our pre-specified subgroup analyses were appropriate or possible. Therefore, different types of strategies were analysed separately and new subgroups were defined within these prior to analysis. These new analyses are listed in tables 1- 4.

Absolute benefits of effective retention strategies were based on applying meta-analysis risk ratios to representative control arm retention rates[20][20]. All statistical analyses were conducted using RevMan5.

Formatted: Do not check spelling or grammar, Superscript

Results

For peer review only - http://bmjopen.bmj.com/site/about/guidelines.xhtml

BMJ Open REVIEW submitted 07.08.2013

We identified 38 eligible randomised retention trials from 24,304 records (Fig 4). Twenty-eight of these were published in full[13-18;21-38][~~13-18,21-38~~], two in the grey literature[14;34][~~14,34~~] and eight are unpublished (*unpublished trials by Edwards, Svobodva, Letley, Maclennan, Land, Bailey 1, Bailey 2 Marson*). Unpublished trials were identified by word of mouth, reference lists of relevant literature and a survey of UK clinical trials units. Four retention trial publications contained two trials each[18;32;33;35][~~18,32,33,35~~].

Participants and settings

Eligible retention trials were from different geographical areas and clinical settings. Clinical areas ranged from exercise and alcohol dependency to treatment and screening for cancer (Tables 1- 4)[12][~~12~~].

Outcomes for strategies to improve retention were measured by: return of postal or electronic questionnaires[13-15;18;21;22;24;25;27;29-34;36-41][~~13-15,18,21,22,24,25,27,29-34,36-41~~] (*unpublished trials by Edwards, Svobodva, Letley, Maclennan, Land, Bailey 1, Bailey 2 Marson*) or biomedical data [17][~~17~~] (*Bailey unpublished*) a combination of postal, telephone, and email follow-up[35][~~35~~] or face to face follow-up/retention[16;42][~~16,42~~].

Design of included retention trials

One retention trial was cluster randomised (*Land unpublished*), four were factorial trials[13-16][~~13-16~~] and there was one three-armed[17][~~17~~] and three four-armed trials[18;32][~~18,32~~]. Five trials were quasi randomised[16;29;33;42][~~16,29,33,42~~], allocating participants by either their identification numbers[29;42][~~29,42~~], day of clinic visit[16][~~16~~] or by random selection of half the sample for the intervention and half for the control group[33][~~33~~]. All strategies targeted individual trial participants except one which targeted sites (*Land unpublished*).

Twenty nine retention trials commenced during follow-up of the host trial[13;15;16;18;21;22;24-27;29-36;38;42;43][~~13,15,16,18,21,22,24-27,29-36,38,42,43~~] (*Edwards, Land, Maclennan, Bailey, Svoboda, unpublished*). One trial followed children of mothers who participated in the MRC ORACLE trial[39][~~39~~]. Two trials followed up participants in smoking cessation trials after the host trial finished[17;40][~~17,40~~]. Another retention trial randomised participants before the host trial commenced[23][~~23~~]. Four trials commenced during the pilot phase of the host trial[18;32;37][~~18,32,37~~]

Formatted: Do not check spelling or grammar, Superscript
Formatted: Do not check spelling or grammar, Superscript
Formatted: Do not check spelling or grammar, Superscript
Formatted: Do not check spelling or grammar, Superscript
Formatted: Do not check spelling or grammar, Superscript
Formatted: Do not check spelling or grammar, Superscript
Formatted: Do not check spelling or grammar, Superscript
Formatted: Do not check spelling or grammar, Superscript
Formatted: Do not check spelling or grammar, Superscript
Formatted: Do not check spelling or grammar, Superscript
Formatted: Do not check spelling or grammar, Superscript
Formatted: Do not check spelling or grammar, Superscript
Formatted: Do not check spelling or grammar, Superscript
Formatted: Do not check spelling or grammar, Superscript
Formatted: Do not check spelling or grammar, Superscript
Formatted: Do not check spelling or grammar, Superscript
Formatted: Do not check spelling or grammar, Superscript
Formatted: Do not check spelling or grammar, Superscript

For peer review only

(*Letley unpublished*). For one trial it is unclear when the retention trial commenced in relation to the host trial[14][14].

Incentive strategies

There were 14 retention trials of incentives and 19 trial comparisons. Thirteen trials investigating incentive strategies targeted questionnaire response, with only one targeting participant retention[16][16]. Incentive strategies aimed at improving questionnaire response were: vouchers[18:29:39][18,29,39], cash[25][25], a charity donation[18][18], entry into a prize draw[14:18:30][14,18,30], cheques[14:17][14,17] offers of study results[24:40][24,40] and a certificate of appreciation[15:16][15,16]. Incentive strategies aimed at participant retention were: lapel pins and a certificate of appreciation[16][16]. UK incentives ranged in value from £5-£20[18:29:39][18,29,39] (*Bailey unpublished*) and from $2-$10 for US based trials, and were provided as either cash or voucher. Offers of entry into prize draws ranged from £25- £250 for UK[18:30][18,30] and $US50 for US based trials[14][14] (Table 1), there was no information available on the chance of winning a prize. One trial evaluated giving a monetary incentive with a promise of a further incentive for return of trial data (*Bailey 2 unpublished*).

Communication strategies

There were 14 retention trials of communication strategies and 20 trial comparisons. Most communication strategies targeted questionnaire response, with only one targeted at the return of biomedical test kits[35][35]. Strategies evaluated were: enhanced letters i.e. those with additional information about trial processes or with an extra feature e.g. signed by a principal investigator[15:15][15] (*Marson unpublished*) use of additional telephone reminders[35][35] (*Maclennan unpublished*); a calendar including reminders of when to return a questionnaire[34][34]; text and/or email reminders[21:31:35][21,31,35] and reminders to sites of upcoming assessments versus no additional reminder (*Land unpublished*). One trial used a package of postal communication strategies called the Total Design Method (TDM)[37][37] and another used recorded delivery of questionnaires[38][38] (Table 2).

Five trials evaluated both communication and incentive strategies[13-15;25;35][13-15,25,35] (Tables 1 and 2). The incentives were: certificates of appreciation for study involvement[15][15], study branded pens[13][13], a US$2 coin[14][14] and a US$5 bill [25][25] or fridge magnets[35][35]. The communication strategies were: 1st or 2nd class outward post[13-15][13-15] stamped and business reply envelopes[13][13], letters signed by different study personnel[15][15], letters posted at different times[15][15], telephone survey[25][25] and text messages[35][35].

New questionnaire formats

Formatted: Do not check spelling or grammar, Superscript
Formatted: Do not check spelling or grammar, Superscript
Formatted: Do not check spelling or grammar, Superscript
Formatted: Do not check spelling or grammar, Superscript
Formatted: Do not check spelling or grammar, Superscript
Formatted: Do not check spelling or grammar, Superscript
Formatted: Do not check spelling or grammar, Superscript
Formatted: Do not check spelling or grammar, Superscript
Formatted: Do not check spelling or grammar, Superscript
Formatted: Do not check spelling or grammar, Superscript
Formatted: Do not check spelling or grammar, Superscript
Formatted: Do not check spelling or grammar, Superscript
Formatted: Do not check spelling or grammar, Superscript
Formatted: Do not check spelling or grammar, Superscript
Formatted: Do not check spelling or grammar, Superscript
Formatted: Do not check spelling or grammar, Superscript
Formatted: Do not check spelling or grammar, Superscript
Formatted: Do not check spelling or grammar, Superscript
Formatted: Do not check spelling or grammar, Superscript
Formatted: Do not check spelling or grammar, Superscript
Formatted: Do not check spelling or grammar, Superscript
Formatted ... [1]
Formatted ... [2]
Formatted ... [3]
Formatted ... [4]
Formatted ... [5]
Formatted ... [6]
Formatted ... [7]
Formatted ... [8]
Formatted ... [9]
Formatted ... [10]

For peer review only

For peer review only - http://bmjopen.bmj.com/site/about/guidelines.xhtml

The effect of a change in questionnaire format on response to questionnaires was evaluated in eight trials. The 10 comparison formats evaluated were (Table 3): questionnaire length[27;32;36][27,32,36] (*Edwards unpublished Svoboda unpublished*) order of questions (Letley *unpublished*)[33][33] and relevance of questionnaires in the context of research in alcohol dependence[32][32].

Formatted: Do not check spelling or grammar, Superscript

Formatted: Do not check spelling or grammar, Superscript

Formatted: Do not check spelling or grammar, Superscript

Behavioural strategies

There were two retention trials of motivational behavioural strategies, one in an exercise trial[26][26] and another in a parenting trial[23][23] (Table 4). A behavioural strategy was defined as giving participants information about goal setting and time management to facilitate successful trial completion. One retention trial was run prior to the host trial[23][23], where only participants who completed the orientation/retention trial were included in the subsequent parenting trial.

Formatted: Do not check spelling or grammar, Superscript

Formatted: Font: (Default) Times New Roman, 12 pt, Font color: Auto

Formatted: Body Text Char, Font: (Default) Times New Roman, 14.5 pt, Font color: Auto

Formatted: Body Text Char, Font: (Default) Times New Roman, 14.5 pt, Font color: Auto

Formatted: Body Text Char, Font: (Default) Times New Roman, 14.5 pt, Font color: Auto

Formatted: Do not check spelling or grammar, Superscript

Case management

Case management defined as outreach, service planning linkage, monitoring, and advocacy, was compared within usual follow-up in a cancer screening trial[28][28](Table 4). This strategy involved trial assistants managing participant follow-up by arranging services to enable participants to keep trial follow-up appointments.

Formatted: Do not check spelling or grammar, Superscript

Methodology strategies

One trial included an open trial versus blind trial design to evaluate the impact on questionnaire response[22][22] (Table 4).

Formatted: Do not check spelling or grammar, Superscript

Trials not included in the meta-analyses

Two included trials could not be included in the meta-analysis[30][30] (*Letley unpublished*). For one, the host trial participants included randomised and non-randomised participants[30][30] and the author confirmed that participants in the retention trial were from both cohorts and these data could not be separated. For the other, retention trial (*Letley unpublished*) outcome data were not available.

Formatted: Do not check spelling or grammar, Superscript

Formatted: Do not check spelling or grammar, Superscript

Risk of bias in included trials

Twenty four trials describe adequate sequence generation[15;16;18;22-24;26;30-32;34;35;37;39;40][15,16,18,22-24,26,30-32,34,35,37,39,40] (*unpublished trials Bailey2 Bailey1 Letley, Land, Maclennan, Marson*). There was insufficient information about the sequence generation for

Formatted: Do not check spelling or grammar, Superscript

For peer review only - http://bmjopen.bmj.com/site/about/guidelines.xhtml

ten trials, but they were all described as randomised[13;14;17;21;25;27;36;38][13,14,17,21,25,27,36,38] (*Edwards, Svoboda unpublished*). Five trials used quasi randomisation[16;28;29;33][16,28,29,33]. Fifteen trials reported both adequate sequence generation and allocation concealment[18;22;24;26;31;32;34;39;40][18,22,24,26,31,32,34,39,40] (*Letley, Maclennan, Bailey* [1,2,] *unpublished*).

Blinding of participants to the intervention was not possible for incentive strategies offers of incentives, behavioural or case management strategies, and different types of communication and questionnaire format strategies and for one trial that evaluated the effect of a blind versus open design on retention this was not applicable[22][22]. For some trials, participants were aware of the intervention but unaware of the evaluation[14;16;23;30;33;39][14,16,23,30,33,39] (*Maclennan, Marson unpublished*). For another trial[26][26] exercise sessions were not separated according to the behavioural intervention i.e. walking and swimming, and potential contamination between groups could have led to bias. For other trials, blinding of participants or trial personnel to the outcome or intervention was not reported. The primary outcome measure for this review was retention, and this was well reported. Authors were contacted for clarification of any exclusions after randomisation if this was unclear from retention trial reports. Although retention trial protocols were not available for included trials, the published and unpublished reports included reported all expected outcomes for retention.

The effects of strategies

1. Incentive Strategies

There were 14 retention trials of incentives, 19 trial comparisons with 16,253 comparisons. Across incentive subgroups there was considerable heterogeneity (p<0.00001) Figure 1a. So we did not pool the results for incentives. Unless otherwise stated results from the random effects model were similar. Three trials (3166 participants) that evaluated the effect of giving monetary incentives to participants showed that the addition of monetary incentives is more effective than no incentive at increasing response to postal questionnaires (RR=1.18; 95% CI 1.09-1.28; p<0.0001, heterogeneity p=0.21 Figure 1a). A sensitivity analysis excluding the quasi randomised trial by Gates shows a similar effect (RR=1.31; 95% CI 1.11-1.55; p=0.002)[29][29]. Also, based on two web based trials (3613 participants, Figure 1a), an offer of a monetary incentive promotes greater return of electronic questionnaires than no offer (RR=1.25; 95% CI 1.14-1.38, p<0.00001, heterogeneity p=0.14). However, a single trial

Formatted: Do not check spelling or grammar, Superscript

Formatted: Do not check spelling or grammar, Superscript

Formatted: Do not check spelling or grammar, Superscript

Formatted: Do not check spelling or grammar, Superscript

Formatted: Do not check spelling or grammar, Superscript

Formatted: Do not check spelling or grammar, Superscript

Formatted: Do not check spelling or grammar, Superscript

For peer review only - http://bmjopen.bmj.com/site/about/guidelines.xhtml

comparison suggests that an offer of a monetary donation to charity does not increase response to electronic questionnaires (RR =1.02, 95% CI; 0.78-1.32; p=0.90 Figure 1a)

Based on three trials (6322 participants) there is no clear evidence that the addition of non-monetary incentives improved questionnaire response (RR=1.00; 95% CI 0.98-1.02; p=0.91) but there is some heterogeneity (p=0.02 Figure 1a). A sensitivity analysis excluding the quasi randomised trial by Bowen showed a similar effect (RR=1.00; 95% CI 0.93-1.08; p=0.99, heterogeneity p=0.01) [1616]. Two trials (1,138 participants) evaluating offers of non-monetary incentives suggest that an offer of a non-monetary incentive is neither more nor less effective than no offer (RR=0.99; 95% CI 0.95-1.03; p=0.60; heterogeneity p=0.52) at improving questionnaire response Figure 1a.

Formatted: Do not check spelling or grammar, Superscript

In exploratory analyses, the different incentive arms that were combined for the main analysis do not appear to show differential effects (Figure 5).

Two trials (902 participants) show that higher value incentives are better at increasing response to postal questionnaires than lower value incentives (RR 1.12; 95% CI 1.04-1.22; p =0.005; heterogeneity p=0.39) irrespective of how they are given (Figure 1b).

Two trial comparisons (297 participants) provide no clear evidence that giving a monetary incentive is better than an offer of entry into a prize draw for improving response to postal questionnaires (RR=1.04; 95% CI 0.91- 1.19; p=0.56, heterogeneity p=0.18, Figure 1c).

One trial could not be included in the analysis[3030], but showed a higher response in the group offered entry into a prize draw (70.5%) compared with the group not offered entry into the draw (65.8%).

Formatted: Do not check spelling or grammar, Superscript

2. Communication strategies

There were 14 trials of communication strategies and 20 comparisons with 9,822 participants. The communication strategies were so diverse that these were analysed separately.

Results from two trials (2479 participants) show that an enhanced letter is neither more nor less effective than a standard letter for increasing response to postal questionnaires (RR=1.01; 95% CI 0.97-1.05; p=0.70; heterogeneity p=0.80, Figure 2a) . Although based on a single trial (226 participants), the TDM package seems much more effective than a customary postal

For peer review only

For peer review only - http://bmjopen.bmj.com/site/about/guidelines.xhtml

communication method at increasing questionnaire return (RR=1.43, 95% CI 1.22-1.67; p<0.0001 Figure 2b). Based on the relevant arms of three trials (1888 participants), there is no clear evidence that priority post is either more or less effective than regular post at increasing trial questionnaire return (RR=1.02; 95% CI 0.95-1.09; p=0.55; heterogeneity p=0.53 Figure 2c).

Six trials (3401 participants) evaluated the effect of different types of reminders to participants on questionnaire response. There is no clear evidence that a reminder is either more or less effective than no reminder (RR=1.03; 95% CI 0.99-1.06; p=0.13; heterogeneity p=0.73) at improving trial questionnaire response (Figure 2d). One trial (700 participants) showed no clear evidence that a telephone survey is either more or less effective than a monetary incentive and a questionnaire for improving questionnaire response (RR=1.08; 95% CI 0.94-1.24; p=0.27, Fig 2e). Based on one cluster randomised trial (272 participants), a monthly reminder to sites of upcoming assessment was neither more nor less effective than the usual follow-up (RR=0.96; 95% CI 0.83-1.11; p=0.57). However, one small trial (192 participants) suggested that recorded delivery is more effective than a telephone reminder (RR= 2.08; 95% CI 1.11-3.87; p=0.02). Based on one other trial (664 participants), there is no clear evidence that sending questionnaires early increased or decreased response (RR=1.10; 95% CI 0.96-1.26; p=0.19).

3. New questionnaire strategies

Eight trials with ten comparisons (21,505 participants) evaluated the effect of a new questionnaire format on questionnaire response. Although there is only some ~~modest~~ heterogeneity between the questionnaire subgroups p=0.11 (Figure 3), it did not seem reasonable to pool the results based on such different interventions.

Five trials (7277 participants) compared the effect of short questionnaires versus long on postal questionnaire response. There is only a suggestion that short questionnaires may be better (RR=1.04; 95% CI 1.00-1.08; p=0.07, heterogeneity p=0.14, Figure 3). Based on one trial (900 participants), there is no clear evidence that long and clear questionnaires are more or less effective than shorter condensed questionnaires for increasing questionnaire response (RR= 1.01, 0.95-1.07; p=0.86, Figure 3). Two quasi randomised trials (9435 participants) also show no good evidence that placing disease/condition questions before generic questions is either more or less effective than vice versa at increasing questionnaire response (RR=1.00,

For peer review only - http://bmjopen.bmj.com/site/about/guidelines.xhtml

0.97-1.02; p=0.75, heterogeneity (p=0.44), Figure 3). One trial by Letley (*unpublished*) not included in this analysis, provided no estimate of effect.

In the context of research on reducing alcohol consumption there is also evidence that more relevant questionnaires i.e. those relating to alcohol use, increase response rates (RR 1.07; 95% CI 1.01-1.14; p= 0.03, Figure 3).

4. Behavioural / motivational strategies

Two community based trials (273 participants) show no clear evidence that the behavioural / motivational strategies used are either more or less effective than standard information for retaining participants (RR= 1.08; 95% CI 0.93-1.24; p=0.31 heterogeneity p=0.93)

5. Case management strategies

One trial (703 participants) evaluated the effect of intensive case management procedures on retention. There is no evidence that intensive case management is either more or less effective than usual follow-up in the population examined (RR=1.00; 95% CI 0.97-1.04; p=0.99)

6. Methodology strategies

One fracture prevention trial (538 participants) evaluated the effect of participants knowing their treatment allocation (open trial) compared to participants blind/unaware of their allocation on questionnaire response. The open design led to higher response rates (RR=1.37; 95% CI 1.16 -1.63; p=0.0003).

Absolute benefits of strategies to improve retention

The absolute benefits of effective strategies on typical questionnaire response are illustrated in Table 5. Based on a 40% baseline response rate for postal questionnaires, the addition of a monetary incentive is estimated to increase response by 92 questionnaires per 1000 sent (95% CI 50-131). With a baseline response rate of 30%, as seen in the included online trial, the addition of an offer of a monetary incentive is estimated to increase response by 140 questionnaires per 1000 (95% CI 86-193).

Discussion

Thirty-eight randomised retention trials were included in this review, evaluating six broad types of strategies to increase questionnaire response and retention in randomised trials. Trials

For peer review only - http://bmjopen.bmj.com/site/about/guidelines.xhtml

were conducted across a spectrum of disease areas, countries, health care, and community settings. Strategies with the clearest impact on questionnaire response were: addition of monetary incentives compared to no incentive for return of postal questionnaires, addition of an offer of a monetary incentive when compared to none for return of electronic questionnaires, and an offer of £20 vouchers when compared to £10 for return of postal questionnaires and biomedical test kits. The evidence was less clear about the effect of shorter questionnaires rather than longer questionnaires and for questionnaires of greater relevance to the questions being studied. Recorded delivery of questionnaires, the Total Design Method a "package" of postal communication strategies with reminder letters and an open trial design appear more effective than standard procedures. These strategies were tested in single trials and may need further evaluation. The addition of a non-monetary incentive or an offer of a non-monetary incentive compared to no incentive did not increase or decrease trial questionnaire response. "Enhanced" letters, letters delivered by priority post or additional reminders were also no more effective than standard communication. Altering questionnaire structure does not seem to increase response. No strategy had a clear impact on increasing the number of participants returning to sites for follow-up.

Strengths and weaknesses

This is the most comprehensive review of strategies specifically designed to improve retention in randomised trials, including many unpublished trials and data. Although our searches were extensive, some less well reported, on-going, or unpublished trials, or trials conducted outside the UK might have been missed.

Most trials used appropriate methods for randomisation or at least stated that they were randomised. For trials that did not describe their methods well or provide further information, there remains a potential risk of selection bias. Sensitivity analyses excluding quasi-randomised trials did not affect the results. In this context, where motivating participants to provide data or attend clinics is often the target of the interventions and so appropriately influences the outcome, lack of blinding is less of a concern. Retention is the outcome and was obtained for all but two trials so similarly, attrition and selective outcome reporting bias are probably unimportant. Although the retention trials were fairly well conducted, this could be improved, and they were often poorly reported. This may be because they were designed

For peer review only - http://bmjopen.bmj.com/site/about/guidelines.xhtml

BMJ Open REVIEW submitted 07.08.2013

when loss to follow-up became a problem in a trial, rather than pre planned prior to host trial commencement.

Few trials are available for behavioural, case management and methodological strategies (only one or two each) and this affects the power of the result for these strategies. The use of open trials to increase questionnaire response can only be applied to trials where blinding is not required, based on our result this strategy would need to be evaluated in different trial contexts if it were to be applied in other areas. All included studies were conducted in higher income countries. Therefore, the effective strategies may not be socially, culturally or economically appropriate to trials conducted in low resource settings. The diversity of strategies and the low number of trials meant that we could not examine the impact of, for example, trial setting and disease area as planned. Moreover, most of the evidence relates to increasing questionnaire response rather than participant retention in follow-up. Many trials require participants to return to sites for follow-up and monitoring; however barriers to follow-up do exist and are trial and participant specific depending on the disease area, treatment and population group. Return for follow-up at sites depends upon participant preferences and the demands of the trial.[44] Barriers to follow-up at site could be alleviated by using tailored strategies to encourage participants to return to sites for follow-up and monitoring. Studies that evaluate such strategies are particularly needed.

Formatted: Do not check spelling or grammar, Superscript

Edwards extensive review of methods to increase response to postal and electronic questionnaires found that monetary incentives and recorded delivery of questionnaires improved response[77]. However, unlike our review they also found that non-monetary incentives, shorter questionnaires, use of handwritten addresses, stamped return envelopes (as opposed to franked return envelopes) and, first class outward mailing were effective. We did however find that a "package" including an enhanced letter with several reminders was effective. The trials included in the Edwards review were embedded in surveys, cohort studies and trials and there was substantial heterogeneity in the results, which was not a particular problem in this review [77]. Moreover, we included seven unpublished trials and 18 other trials not included by Edwards[1212].

Formatted: Do not check spelling or grammar, Superscript

Formatted: Do not check spelling or grammar, Superscript

Formatted: Do not check spelling or grammar, Superscript

Nakash's small systematic review of ways to increase response to postal questionnaires in health care was not exclusive to randomised trials[4544]. They found reminder letters, telephone contact, and short questionnaires increased response to postal questionnaires. There was no

Formatted: Do not check spelling or grammar, Superscript

16

For peer review only

evidence that incentives were effective. A systematic review of methods to increase retention in population based cohort studies had no meta-analysis, but suggested that incentives were associated with increased retention[66].

Formatted: Do not check spelling or grammar, Superscript

Prior to our review, it was not clear which if any of these strategies could be extrapolated to randomised trials. We also identified additional strategies that may improve trial questionnaire response or retention for example, methodological strategies.

Implications

Formatted: Font: (Default) Times New Roman, 12 pt, Font color: Auto

Formatted: Justified, Space After: 0 pt, Line spacing: 1.5 lines

Although giving monetary incentives up front seems effective, offering and giving these after receipt of data could be a cost effective strategy, because those not returning questionnaires would not receive an incentive. The addition of non-monetary incentives for example, lapel pins and certificates of appreciation, or offers of these did not increase response or retention, perhaps because these items are not valued by participants. Offers of monetary incentives were also an effective strategy in the context of an online electronic questionnaire, thus it would be beneficial for trialists to know which is more effective: an offer of a monetary incentive or an upfront monetary incentive in a head to head trial comparison.

The value of incentives used in UK evaluations ranged from GBP5 to GBP20 and for US-based studies was USD2 to USD10. For offers of entries into prize draws, the values were higher, ranging from GBP25 to GBP250 for UK prize draws and USD50 for US-based prize draws. The value of monetary incentive should not be so high as to be perceived as payment or coercion for data but more as an appreciation for efforts made by participants. A cost effectiveness analysis for additional responses gained after incentive strategies were introduced was reported for only some incentive trials. As costs increase the cost benefit associated with incentive strategies would need to be updated if incentives were to be used to improve retention in future trials [25;29;39 18;30].

Priority post, enhanced letters (e.g. signed by the principal investigator) and different types of additional reminders are used by trialists in current research practice, but were not found to be effective. The former may not be considered important and too many reminders, over and above standard procedures, could be counterproductive.

For peer review only - http://bmjopen.bmj.com/site/about/guidelines.xhtml

BMJ Open REVIEW submitted 07.08.2013

Although appearing very effective, the total design method for postal questionnaires could be labour intensive to implement, expensive, and may no longer be applicable to some participant groups e.g. young people used to other modes of communication, or in trials using email, text or online data collection. Recorded delivery could be useful to ensure trial follow-up supplies reach their intended destination, but careful planning to avoid inconvenience for the participant might be necessary. Open trials to increase questionnaire response can only be used where blinding is not required. This could be counterproductive, however, as unblinded trials can cause biased outcome assessment or loss to follow-up if a participant or clinician has a treatment preference.

Questionnaire length and relevance may need further evaluation as there is only a suggestion that these are effective in the context of randomised trials. Also, telephone follow-up compared with a monetary incentive sent with a questionnaire needs further evaluation possibly with a cost benefit analysis as both could be expensive in time and human resources. Evaluations of strategies that encourage participants to return to sites for follow-up visits and monitoring are particularly needed because many trials collect outcome data in this way.

Trialists should consider including well thought out and adequately powered evaluations of strategies to increase retention in randomised trials with a clear definition of retention strategies and retention measures. Trialists could incorporate evaluations of strategies to improve retention at the design stage so that power, sample size and funding are taken into account. Retention trials were often poorly reported and trialists should adhere to the consort guidelines for trial reporting to facilitate the synthesis of results in future methodology reviews.

There is less research on ways to increase return of participants to trial sites for follow-up and on the effectiveness of strategies to retain trial sites in cluster and individual randomised trials. Research in both areas would be very beneficial to trialists. Application of the results of this review would depend on trial setting, population, disease area, budget allowance and follow-up procedures.

Conclusions

For peer review only - http://bmjopen.bmj.com/site/about/guidelines.xhtml

BMJ Open REVIEW submitted 07.08.2013

Trialists should consider using monetary incentives and offers of monetary incentives to increase postal and electronic questionnaire response, depending on trial setting, population, disease area, budget, and usual follow-up procedures.

Future evaluations of retention strategies in randomised trials should be carefully planned and adequately powered, and the retention strategies and measures of retention clearly defined. More research on ways to increase return of participants to sites for follow-up, and on ways to retain sites in cluster and individual randomised trials are also needed.

Acknowledgements

We would like to thank the following: all authors of included published trials for providing extra unreported data; principal investigators for data on trials in progress or completed and unpublished (Julia Bailey UCL for data for Bailey 1 and Bailey 2; Graeme MacLennan for data for MacLennan; Stephanie Land data for Land) and the coordinators of UK Clinical Trials Units who responded to our survey with information about on going and/or unpublished completed trials. We also thank Cara Booker, SPHRU for search strategy information; Angela Young, Librarian UCL for assistance with searching databases and Ian White MRC Bio Statistics Unit Cambridge for helpful comments on the analysis of multi arm trials. We also acknowledge Shaun Treweek, Mike Clarke, Andy Oxman, Karen Robinson and Anne Eisinga for comments on the review protocol; and Phil Edwards, Elie Akl, Lisa Maguire, Jean Suvan, Shaun Treweek, Mike Clarke and Karen Robinson for comments on the review. This project was funded by the MRC Population Health Sciences Research Network (Grant Number PHSRN 30).

Statement of funding and competing interests

All authors have completed the ICMJE uniform disclosure form and declare no competing interests. This project was funded by the Medical Research Council Population Health Sciences Research Network grant number PHSRN 30.

For peer review only

Article summary

Article focus

Loss to follow-up in randomised trials can cause bias and loss of power.

Many strategies are routinely used in an attempt to improve retention in randomised trials.

The effect of strategies used to improve retention in randomised trials has not been formally evaluated until now. This systematic review identifies strategies that have been evaluated in randomised trials and quantifies the effect of these strategies to improve retention in randomised trials.

Key messages

This is the first systematic review to evaluate the effect of strategies to improve retention in randomised trials.

Effective strategies for increasing postal questionnaire response were: monetary incentives, offers of monetary incentives, and higher valued incentives.

Strategies that encourage participant to return to sites for follow-up visits and monitoring are particularly needed. Other strategies need further evaluation.

Such evaluations need to be rigorous and adequately reported

Strengths and limitations of this study

This is the most comprehensive review of strategies specifically designed to improve retention in randomised trials, including many unpublished trials and data.

Although our searches were extensive, some less well reported, on-going, or unpublished trials, or trials conducted outside the UK might have been missed.

For peer review only

BMJ Open REVIEW submitted 07.08.2013

Reference List

(1) Fewtrell MS, Kennedy K, Singhal A, Martin RM, Ness A, Hadders-Algra M et al. How much loss to follow-up is acceptable in long-term randomised trials and prospective studies? *Arch Dis Child* 2008; 93(6):458-461.

(2) Schulz KF, Grimes DA. Sample size slippages in randomised trials: exclusions and the lost and wayward. *The Lancet* 2002; 359(9308):781-785.

(3) Hollis S, Campbell F. What is meant by intention to treat analysis? Survey of published randomised controlled trials. *BMJ* 1999; 319:670-674.

(4) Robinson KA, Dennison CR, Wayman DM, Pronovost PJ, Needham DM. Systematic review identifies number of strategies important for retaining study participants. *J Clin Epidemiol* 2007; 60(8):757.

(5) Davis L, Broome M, Cox R. Maximizing Retention in Community-based Clinical Trials. *Journal of Nursing Scholarship* 2002; 34(1):47-53.

(6) Booker C, Harding S, Benzeval M. A systematic review of the effect of retention methods in population-based cohort studies. *BMC Public Health* 2011; 11(1):249.

(7) Edwards PJ, Roberts IG, Clarke MJ, DiGuiseppi C, Wentz R, Kwan I et al. Methods to increase response rates to postal and electronic questionnaires. *Cochrane Database of Systematic Reviews 2009, Issue 3 Art No : MR000008* 2009;(3).

(8) Brueton VC, Rait G, Tierney J, Meredith S, Darbyshire J, Harding S et al. Strategies to reduce attrition in randomised trials. *Cochrane Database of Systematic Reviews Art No :MR000032 DOI: 10 1002/14651858 MR000032* 2011;(2).

Formatted: Font: 12 pt, Do not check spelling or grammar

Formatted: Space After: Auto

Formatted: Font: Italic, Do not check spelling or grammar

Formatted: Font: Italic, Do not check spelling or grammar

Formatted: Font: Italic, Do not check spelling or grammar

Formatted: Font: Italic, Do not check spelling or grammar

Formatted: Font: Italic, Do not check spelling or grammar

Formatted: Font: Italic, Do not check spelling or grammar

Formatted: Font: Italic, Do not check spelling or grammar

Formatted: Font: AGaramond-Regular, Do not check spelling or grammar

Formatted: Font: Italic, Do not check spelling or grammar

For peer review only

(9) Higgins J, Altman D. Assessing risk of bias in included studies. In: Higgins J, Sally Green, editors. Cochrane Hanbook for Systematic Reviews of Interventions. John Wiley; 2008. 187-242.

(10) Higgins J, Deeks.J, Altman D. Special topics in statistics. In: Julian PT Higgins, Sally Green, editors. Cochrane Handbook for Systematic Reviews of Intervenions. John Wiley; 2008. 482-529.

(11) University of Aberdeen. Aberdeen ICCs. 2013.

Ref Type: Online Source

(12) Brueton VC, Tierney J, Stenning S, Nazareth I, Meredith S, Harding S et al. Strategies to improve retention in randomised trials . *Cochrane Methodology Group* 2013; in press.

(13) Sharp L, Cochran C, Cotton SC, Gray NM, Gallagher ME. Enclosing a pen with a postal questionnaire can significantly increase the response rate. *J Clin Epidemiol* 2006; 59(7):747-754.

(14) Kenton L, Dennis CL, Weston J, Kiss A. Abstracts from the 28th Meeting of the Society of Clinical Trials, Montreal, May 20–23, 2007:The effect of incentives and high priority mailing on postal questionnaire response rates: A Mini-RCT. Clinical Trials 4[4], 371-455. 1-8-2007.

Ref Type: Abstract

(15) Renfroe EG, Heywood G, Foreman L, Schron E, Powell J, Baessler C et al. The end-of-study patient survey: methods influencing response rate in the AVID Trial. *Control Clin Trials* 2002; 23(5):521-533.

(16) Bowen D, Thornquist M, Goodman G, Omenn GS, Anderson K, Barnett M et al. Effects of Incentive Items on Participation in a Randomized Chemoprevention Trial. *J Health Psychol* 2000; 5(1):109-115.

(17) Bauer JE, Rezaishiraz H, Head K, Cowell J, Bepler G, Aiken M et al. Obtaining DNA from a geographically dispersed cohort of current and former smokers: Use of mail-based mouthwash collection and monetary incentives. *Nicotine & Tobacco Research* 2004; 6(3):439-446.

(18) Khadjesari Z, Murray E, Kalaitzaki E, White I, Mc Cambridge J, Thompson S et al. Impact and costs of incentives to reduce attrition in online trials:Two randomised controlled trials. *Journal of Medical Internet Research* 2011; 13(1):e26.

(19) Higgins JPT, Thompson SG, Deeks JJ, Altman DG. Measuring inconsistency in meta-analyses. *BMJ* 2003; 327(7414):557-560.

(20) Schunemann H, Oxman AD, Visr G, Higgins J, Deeks D, Glasziou P et al. Interpreting results and drawing conclusions. In: Higgens J, Green S, editors. Cochrane Handbook

Formatted: Space After: Auto

Formatted: Font: Italic, Do not check spelling or grammar

Formatted: Font: Italic, Do not check spelling or grammar

Formatted: Font: Symbol, Do not check spelling or grammar

Formatted: Space After: Auto

Formatted: Font: Italic, Do not check spelling or grammar

Formatted: Font: Italic, Do not check spelling or grammar

Formatted: Font: Italic, Do not check spelling or grammar

Formatted: Font: Italic, Do not check spelling or grammar

Formatted: Font: Italic, Do not check spelling or grammar

For peer review only

For peer review only - http://bmjopen.bmj.com/site/about/guidelines.xhtml

BMJ Open REVIEW submitted 07.08.2013

for Systematic Reviews of Interventions. Chichester West Sussex: John Wiley and Sons Ltd; 2008. 359-387.

(21) Ashby R, Turner G, Cross B, Mitchell N, Torgerson D. A randomized trial of electronic reminders showed a reduction in the time to respond to postal questionnaires. *J Clin Epidemiol* 2011; 64(2):208-212.

(22) Avenell A, Grant AM, McGee M, McPherson G, Campbell MK, McGee MA et al. The effects of an open design on trial participant recruitment, compliance and retention - a randomized controlled trial comparison with a blinded, placebo-controlled design. *Clinical Trials* 2004; 1(6):490-498.

(23) Chaffin M, Valle LA, Funderburk B, Gurwitch R, Silovsky J, Bard D et al. A Motivational Intervention Can Improve Retention in PCIT for Low-Motivation Child Welfare Clients. *Child Maltreatment* 2009; 14(4):356-368.

(24) Cockayne S, Torgerson D. A randomised controlled trial to assess the effectiveness of offering study results as an incentive to increase response rates to postal questionnaires [ISRCTN26118436]. *BMC Medical Research Methodology* 2005; 5(1):34.

(25) Couper PM, Peytchev A, Strecher JV, Rothert K, Anderson J. Following Up Nonrespondents to an Online Weight Management Intervention: Randomized Trial Comparing Mail versus Telephone. *J Med Internet Res* 2007; 9(2):e16.

(26) Cox KL, Burke V, Beilin LJ, Derbyshire AJ, Grove JR, Blanksby BA et al. Short and long-term adherence to swimming and walking programs in older women -- The Sedentary Women Exercise Adherence Trial (SWEAT 2). *Prev Med* 2008; 46(6):511-517.

(27) Dorman P, Slattery J, Farrell B, Dennis MS, Sandercock PA. A randomised comparison of the EuroQol and Short Form-36 after stroke. United Kingdom collaborators in the International Stroke Trial. *BMJ* 1997; 315(7106):461.

(28) Ford ME, Havstad S, Vernon SW, Davis SD, Kroll D, Lamerato L et al. Enhancing Adherence Among Older African American Men Enrolled in a Longitudinal Cancer Screening Trial. *Gerontologist* 2006; 46(4):545-550.

(29) Gates S, Williams M, Withers E, Williamson E, Mt-Isa S, Lamb S. Does a monetary incentive improve the response to a postal questionnaire in a randomised controlled trial? The MINT incentive study. *Trials* 2009; 10(1):44.

(30) Leigh Brown AP, Lawrie H, Kennedy A, Webb A, Torgerson D, Grant A. Cost effectiveness of a prize draw on response to a postal questionnaire:results of a randomised trial among orthopaedic outpatients in Edinburgh. *Journal of Epidemiology and Public Health* 1997; 51:463-464.

Formatted: Font: Italic, Do not check spelling or grammar

Formatted: Font: Italic, Do not check spelling or grammar

Formatted: Font: Italic, Do not check spelling or grammar

Formatted: Font: Italic, Do not check spelling or grammar

Formatted: Font: Italic, Do not check spelling or grammar

Formatted: Font: Italic, Do not check spelling or grammar

Formatted: Font: Italic, Do not check spelling or grammar

Formatted: Underline, Do not check spelling or grammar

Formatted: Font: Italic, Do not check spelling or grammar

Formatted: Font: Italic, Do not check spelling or grammar

Formatted: Font: Italic, Do not check spelling or grammar

For peer review only - http://bmjopen.bmj.com/site/about/guidelines.xhtml

BMJ Open REVIEW submitted 07.08.2013

(31) Man MS, Tilbrook HE, Jayakody S, Hewitt CE, Cox H, Cross B et al. Electronic reminders did not improve postal questionnaire response rates or response times: a randomized controlled trial. *J Clin Epidemiol* 2011; 64(9):1001-1004.

(32) McCambridge J, Kalaitzaki E, White RI, Khadjesari Z, Murray E, Linke S et al. Impact of Length or Relevance of Questionnaires on Attrition in Online Trials: Randomized Controlled Trial. *J Med Internet Res* 2011; 13(4):e96.

(33) McColl EM, Eccles MPM, Rousseau NSB, Steen INP, Parkin DWD, Grimshaw JMP. From the Generic to the Condition-specific?: Instrument Order Effects in Quality of Life Assessment. [Article]. *Med Care* 2003; 41(7):777-790.

(34) Nakash R. A study of response and non-response to postal questionnaire follow-up in clinical trials. Chapter 6: A radomised controlled trial of a method of improving response to postal questionnaire follow-up in a clinical trial. [University of Warwick; 2007.

(35) Severi E, Free C, Knight R, Robertson S, Edwards P, Hoile E. Two controlled trials to increase participant retention in a randomized controlled trial of mobile phone-based smoking cessation support in the United Kingdom. *Clinical Trials* 2011; 8(5):654-660.

(36) Subar AF, Ziegler RG, Thompson FE, Johnson CC, Weissfeld JL, Reding D et al. Is Shorter Always Better? Relative Importance of Questionnaire Length and Cognitive Ease on Response Rates and Data Quality for Two Dietary Questionnaires. *Am J Epidemiol* 2001; 153(4):404-409.

(37) Sutherland HJ, Beaton M, Mazer R, Kriukov V, Boyd NF. A randomized trial of the total design method for the postal follow-up of women in a cancer prevention trial. *Eur J Cancer Prev* 1996; 5(3):165-168.

(38) Tai SS, Nazareth I, Haines A, Jowett C. A randomized trial of the impact of telephone and recorded delivery reminders on the response rate to research questionnaires. *J Public Health* 1997; 19(2):219-221.

(39) Kenyon S, Pike K, Jones D, Taylor D, Salt A, Marlow N et al. The effect of a monetary incentive on return of a postal health and development questionnaire: a randomised trial [ISRCTN53994660]. *BMC Health Services Research* 2005; 5(1):55.

(40) JR Hughes. Free reprints to increase the return of follow-up questionnaires. Controlled Clinical Trials . 1989.

Ref Type: Abstract

(41) The International Stroke Trial (IST): a randomised trial of aspirin, subcutaneous heparin, both, or neither among 19[punctuation space]435 patients with acute ischaemic stroke. *The Lancet* 1997; 349(9065):1569-1581.

Formatted: Font: Italic, Do not check spelling or grammar

Formatted: Font: Italic, Do not check spelling or grammar

Formatted: Font: Italic, Do not check spelling or grammar

Formatted: Font: Italic, Do not check spelling or grammar

Formatted: Font: Italic, Do not check spelling or grammar

Formatted: Font: Italic, Do not check spelling or grammar

Formatted: Font: Italic, Do not check spelling or grammar

Formatted: Font: Italic, Do not check spelling or grammar

Formatted: Space After: Auto

Formatted: Font: Italic, Do not check spelling or grammar

For peer review only - http://bmjopen.bmj.com/site/about/guidelines.xhtml

BMJ Open REVIEW submitted 07.08.2013

(42) Ford ME, Havstad S, Vernon SW, Davis SD, Kroll D, Lamerato L et al. Enhancing Adherence Among Older African American Men Enrolled in a Longitudinal Cancer Screening Trial. *Gerontologist* 2006; 46(4):545-550.

Formatted: Font: Italic, Do not check spelling or grammar

(43) Marson A, Appleton R, BakerG, Chadwick D, Doughty J, Eaton B et al. A randomised controlled trial examining the longer-term outcomes of standard versus new antiepileptic drugs The SANAD trial. *NIHR HTA Report* 2007; 11(37).

Formatted: Font: Italic, Do not check spelling or grammar

(44) Ross S, Grant A, Counsell C, Gillespie W, Russell I, Prescott R. Barriers to Participation in Randomised Controlled Trials: A Systematic Review. *J Clin Epidemiol* 1999; 52(12):1143-1156.

Formatted: Font: Italic, Do not check spelling or grammar

(45) Nakash R, Hutton J, Jorstad-Stein E, Gates S, Lamb S. Maximising response to postal questionnaires - A systematic review of randomised trials in health research. *BMC Medical Research Methodology* 2006; 6(1):5.

Formatted: Font: Italic, Do not check spelling or grammar

(46) Boyd N, Cousins M, Lockwood G, Tritchler D. Dietary fat and breast cancer risk: The feasibility of a clinical trial of breast cancer prevention. *Lipids* 1992; 27(10):821-826.

Formatted: Font: Italic, Do not check spelling or grammar

(47) Buys S, Partridge E, Greene M, Prorok P, Reding D, Riley T et al. Ovarian cancer screening in the Prostate, Lung, Colorectal and Ovarian (PLCO) cancer screening trial: Findings from the initial screen of a randomized trial. *Am J Obstet Gynecol* 2005; 193(5):1630-1639.

Formatted: Font: Italic, Do not check spelling or grammar

(48) Cooke MW, Marsh JL, Clarke M, Nakash R, Jarvis RM, Hutton JL et al. Treatment of severe ankle sprain: a pragmatic randomised controlled trial comparing the clinical effectiveness and cost effectiveness of three types of mechanical ankle support with tubular bandage. The CAST trial. 13, 1-144. 2009. NIHR Health Technology Assessment Programme.

Ref Type: Report

Formatted: Space After: Auto

(49) CRASH trial collaborators. Effect of intravenous corticosteroids on death within 14 days in 10[punctuation space]008 adults with clinically significant head injury (MRC CRASH trial): randomised placebo-controlled trial. *The Lancet* 2004; 364(9442):1321-1328.

Formatted: Font: Italic, Do not check spelling or grammar

(50) Dennis CL, Hodnett E, Kenton L, Weston J, Zupancic J, Stewart DE et al. Effect of peer support on prevention of postnatal depression among high risk women: multisite randomised controlled trial. *BMJ* 2009; 338(jan15_2):a3064.

Formatted: Font: Italic, Do not check spelling or grammar

(51) Eccles M, McColl E, Steen N, Rousseau N, Grimshaw J, Parkin D et al. Effect of computerised evidence based guidelines on management of asthma and angina in adults in primary care: cluster randomised controlled trial. *BMJ* 2002; 325(7370):941.

Formatted: Font: Italic, Do not check spelling or grammar

(52) Free C, Knight R, Robertson S, Whittaker R, Edwards P, Zhou W et al. Smoking cessation support delivered via mobile phone text messaging (txt2stop): a single-blind, randomised trial. *The Lancet* 2011; 378(9785):49-55.

Formatted: Font: Italic, Do not check spelling or grammar

For peer review only - http://bmjopen.bmj.com/site/about/guidelines.xhtml

BMJ Open REVIEW submitted 07.08.2013

(53) Gail MH, Byar DP, Pechacek TF, Corle DK. Aspects of statistical design for the community intervention trial for smoking cessation (COMMIT). *Control Clin Trials* 1992; 13(1):6-21.

(54) Hughes JR, Hatsukami D, Pickens R, Krahn D, Malin S, Luknic A. Effect of nicotine on the tobacco withdrawal syndrome. *Psychopharmacology (Berl)* 1984; 83(1):82-87.

(55) Kenyon SL, Taylor DJ, Tarnow-Mordi W. Broad-spectrum antibiotics for preterm, prelabour rupture of fetal membranes: the ORACLE I randomised trial. *The Lancet* 2001; 357(9261):979-988.

(56) Kenyon SL, Taylor DJ, Tarnow-Mordi W. Broad-spectrum antibiotics for spontaneous preterm labour: the ORACLE II randomised trial. *The Lancet* 2001; 357(9261):989-994.

(57) Leigh Brown A, Kennedy A, Torgerson D, Campbell J, Webb J, Grant A. The OMENS trial: opportunistic evaluation of musculo-skeletal physician care among orthopaedic outpatients unlikely to require surgery. *Health Bull (Edinb)* 2001; 59(3):198-210.

(58) Marson AG, Al Kharusi AM, Alwaidh M, Appleton R, Baker GA, Chadwick DW et al. The SANAD study of effectiveness of valproate, lamotrigine, or topiramate for generalised and unclassifiable epilepsy: an unblinded randomised controlled trial. *The Lancet* 2007; 369(9566):1016-1026.

(59) Lamb S, Gates S, Underwood M, Cooke M, Ashby D, Szczepura A et al. Managing Injuries of the Neck Trial (MINT): design of a randomised controlled trial of treatments for whiplash associated disorders. *BMC Musculoskeletal Disorders* 2007; 8(1):7.

(60) Murray E, McCambridge J, Khadjesari Z, White I, Thompson S, Godfrey C et al. The DYD-RCT protocol: an on-line randomised controlled trial of an interactive computer-based intervention compared with a standard information website to reduce alcohol consumption among hazardous drinkers. *BMC Public Health* 2007; 7(1):306.

(61) Omenn GS, Goodman GE, Thornquist MD, Balmes J, Cullen MR, Glass A et al. Effects of a Combination of Beta Carotene and Vitamin A on Lung Cancer and Cardiovascular Disease. *N Engl J Med* 1996; 334(18):1150-1155.

(62) Porthouse J, Sarah C, Christine K, Lucy S, Elizabeth S, Terry A et al. Randomised controlled trial of calcium and supplementation with cholecalciferol (vitamin D3) for prevention of fractures in primary care. *BMJ* 2005; 330.

(63) The RECORD Trial Group. Oral vitamin D3 and calcium for secondary prevention of low-trauma fractures in elderly people (Randomised Evaluation of Calcium Or vitamin D, RECORD): a randomised placebo-controlled trial. *The Lancet* 2007; 365(9471):1621-1628.

Formatted: Font: Italic, Do not check spelling or grammar

Formatted: Font: Italic, Do not check spelling or grammar

Formatted: Font: Italic, Do not check spelling or grammar

Formatted: Font: Italic, Do not check spelling or grammar

Formatted: Font: Italic, Do not check spelling or grammar

Formatted: Font: Italic, Do not check spelling or grammar

Formatted: Font: Italic, Do not check spelling or grammar

Formatted: Font: Italic, Do not check spelling or grammar

Formatted: Font: Italic, Do not check spelling or grammar

Formatted: Font: Italic, Do not check spelling or grammar

Formatted: Font: Italic, Do not check spelling or grammar

For peer review only - http://bmjopen.bmj.com/site/about/guidelines.xhtml

BMJ Open REVIEW submitted 07.08.2013

(64) Tai S, Nazareth I, Donegan C, Haines A. Evaluation of General Practice Computer Templates. *Methods Inf Med* 1999; 38:177-181.

(65) The Antiarrhythmics versus Implantable Defibrillators (AVID) Investigators. A Comparison of Antiarrhythmic-Drug Therapy with Implantable Defibrillators in Patients Resuscitated from Near-Fatal Ventricular Arrhythmias. *N Engl J Med* 1997; 337(22):1576-1584.

(66) Tilbrook HE, Cox H, Hewitt CE, Kang'ombe AR, Chuang LH, Jayakody S et al. Yoga for Chronic Low Back PainA Randomized Trial. *Ann Intern Med* 2011; 155(9):569-578.

(67) Rothert K, Strecher VJ, Doyle LA, Caplan WM, Joyce JS, Jimison HB et al. Web-based Weight Management Programs in an Integrated Health Care Setting: A Randomized, Controlled Trial[ast]. *Obesity* 2006; 14(2):266-272.

(68) TOMBOLA Group. Cytological surveillance compared with immediate referral for colposcopy in management of women with low grade cervical abnormalities: multicentre randomised controlled trial. *BMJ* 2009; 339(jul28_2):b2546.

(69) TOMBOLA Group. Biopsy and selective recall compared with immediate large loop excision in management of women with low grade abnormal cervical cytology referred for colposcopy: multicentre randomised controlled trial. *BMJ* 2009; 339(jul28_2):b2548.

(70) UK BEAM Trial Team. United Kingdom back pain exercise and manipulation (UK BEAM) randomised trial: effectiveness of physical treatments for back pain in primary care. *BMJ* 2004;bmj.

Formatted: Font: Italic, Do not check spelling or grammar

Formatted: Font: Italic, Do not check spelling or grammar

Formatted: Font: Italic, Do not check spelling or grammar

Formatted: Font: Italic, Do not check spelling or grammar

Formatted: Font: Italic, Do not check spelling or grammar

Formatted: Font: Italic, Do not check spelling or grammar

Formatted: Font: Italic, Do not check spelling or grammar

Formatted: Indent: Left: 0", Hanging: 0.5", Space After: Auto, Line spacing: At least 12 pt, Tab stops: 0.38", Right + 0.5", Left

~~Reference List~~

~~(1) Fewtrell MS, Kennedy K, Singhal A, Martin RM, Ness A, Hadders-Algra M et al. How much loss to follow-up is acceptable in long-term randomised trials and prospective studies? *Arch Dis Child* 2008; 93(6):458-461.~~

~~(2) Schulz KF, Grimes DA. Sample size slippages in randomised trials: exclusions and the lost and wayward. *The Lancet* 2002; 359(9308):781-785.~~

~~(3) Hollis S, Campbell F. What is meant by intention to treat analysis? Survey of published randomised controlled trials. *BMJ* 1999; 319:670-674.~~

~~(4) Robinson KA, Dennison CR, Wayman DM, Pronovost PJ, Needham DM. Systematic review identifies number of strategies important for retaining study participants. *J Clin Epidemiol* 2007; 60(8):757.~~

For peer review only

BMJ Open REVIEW submitted 07.08.2013

(5) Davis L, Broome M, Cox R. Maximizing Retention in Community-based Clinical Trials. *Journal of Nursing Scholarship* 2002; 34(1):47-53.

(6) Booker C, Harding S, Benzeval M. A systematic review of the effect of retention methods in population-based cohort studies. *BMC Public Health* 2011; 11(1):249.

(7) Edwards PJ, Roberts IG, Clarke MJ, DiGuiseppi C, Wentz R, Kwan I et al. Methods to increase response rates to postal and electronic questionnaires. *Cochrane Database of Systematic Reviews 2009, Issue 3 Art No : MR000008* 2009;(3).

(8) Brueton VC, Rait G, Tierney J, Meredith S, Darbyshire J, Harding S et al. Strategies to reduce attrition in randomised trials. *Cochrane Database of Systematic Reviews Art No :MR000032 DOI: 10 1002/14651858 MR000032* 2011;(2).

(9) Higgins J, Altman D. Assessing risk of bias in included studies. In: Higgins J, Sally Green, editors. Cochrane Hanbook for Systematic Reviews of Interventions. John Wiley; 2008. 187-242.

(10) Higgins J, Deeks.J, Altman D. Special topics in statistics. In: Julian PT Higgins, Sally Green, editors. Cochrane Handbook for Systematic Reviews of Intervenions. John Wiley; 2008. 482-529.

(11) University of Aberdeen. Aberdeen ICCs. 2013.

Ref Type: Online Source

(12) Brueton VC, Tierney J, Stenning S, Nazareth I, Meredith S, Harding S et al. Strategies to improve retention in randomised trials . *Cochrane Methodology Group* 2013; in press.

(13) Sharp L, Cochran C, Cotton SC, Gray NM, Gallagher ME. Enclosing a pen with a postal questionnaire can significantly increase the response rate. *J Clin Epidemiol* 2006; 59(7):747-754.

(14) Kenton L, Dennis CL, Weston J, and Kiss A. Abstracts from the 28th Meeting of the Society of Clinical Trials, Montreal, May 20–23, 2007:The effect of incentives and high priority mailing on postal questionnaire response rates: A Mini-RCT. *<[11] Journal>* 1-8-2007; 4(4):371-455.

(15) Renfroe EG, Heywood G, Foreman L, Schron E, Powell J, Baessler C et al. The end-of-study patient survey: methods influencing response rate in the AVID Trial. *Control Clin Trials* 2002; 23(5):521-533.

(16) Bowen D, Thornquist M, Goodman G, Omenn GS, Anderson K, Barnett M et al. Effects of Incentive Items on Participation in a Randomized Chemoprevention Trial. *J Health Psychol* 2000; 5(1):109-115.

For peer review only

For peer review only - http://bmjopen.bmj.com/site/about/guidelines.xhtml

BMJ Open REVIEW submitted 07.08.2013

(17) Bauer JE, Rezaishiraz H, Head K, Cowell J, Bepler G, Aiken M et al. Obtaining DNA from a geographically dispersed cohort of current and former smokers: Use of mail-based mouthwash collection and monetary incentives. *Nicotine & Tobacco Research* 2004; 6(3):439-446.

(18) Khadjesari Z, Murray E, Kalaitzaki E, White I, Mc Cambridge J, Thompson S et al. Impact and costs of incentives to reduce attrition in online trials:Two randomised controlled trials. *Journal of Medical Internet Research* 2011; 13(1):e26.

(19) Higgins JPT, Thompson SG, Deeks JJ, Altman DG. Measuring inconsistency in meta-analyses. *BMJ* 2003; 327(7414):557-560.

(20) Schunemann H, Oxman AD, Visr G, Higgins J, Deeks D, Glasziou P et al. Interpreting results and drawing conclusions. In: Higgens J, Green S, editors. Cochrane Handbook for Systematic Reviews of Interventions. Chichester West Sussex: John Wiley and Sons Ltd; 2008. 359-387.

(21) Ashby R, Turner G, Cross B, Mitchell N, Torgerson D. A randomized trial of electronic reminders showed a reduction in the time to respond to postal questionnaires. *J Clin Epidemiol* 2011; 64(2):208-212.

(22) Avenell A, Grant AM, McGee M, McPherson G, Campbell MK, McGee MA et al. The effects of an open design on trial participant recruitment, compliance and retention -- a randomized controlled trial comparison with a blinded, placebo-controlled design. *Clinical Trials* 2004; 1(6):490-498.

(23) Chaffin M, Valle LA, Funderburk B, Gurwitch R, Silovsky J, Bard D et al. A Motivational Intervention Can Improve Retention in PCIT for Low-Motivation Child Welfare Clients. *Child Maltreatment* 2009; 14(4):356-368.

(24) Cockayne S, Torgerson D. A randomised controlled trial to assess the effectiveness of offering study results as an incentive to increase response rates to postal questionnaires [ISRCTN26118436]. *BMC Medical Research Methodology* 2005; 5(1):34.

(25) Couper PM, Peytchev A, Strecher JV, Rothert K, Anderson J. Following Up Nonrespondents to an Online Weight Management Intervention: Randomized Trial Comparing Mail versus Telephone. *J Med Internet Res* 2007; 9(2):e16.

(26) Cox KL, Burke V, Beilin LJ, Derbyshire AJ, Grove JR, Blanksby BA et al. Short and long-term adherence to swimming and walking programs in older women -- The Sedentary Women Exercise Adherence Trial (SWEAT 2). *Prev Med* 2008; 46(6):511-517.

(27) Dorman P, Slattery J, Farrell B, Dennis MS, Sandercock PA. A randomised comparison of the EuroQol and Short Form-36 after stroke. United Kingdom collaborators in the International Stroke Trial. *BMJ* 1997; 315(7106):461.

For peer review only - http://bmjopen.bmj.com/site/about/guidelines.xhtml

(28) Ford ME, Havstad S, Vernon SW, Davis SD, Kroll D, Lamerato L et al. Enhancing Adherence Among Older African American Men Enrolled in a Longitudinal Cancer Screening Trial. *Gerontologist* 2006; 46(4):545-550.

(29) Gates S, Williams M, Withers E, Williamson E, Mt-Isa S, Lamb S. Does a monetary incentive improve the response to a postal questionnaire in a randomised controlled trial? The MINT incentive study. *Trials* 2009; 10(1):44.

(30) Leigh Brown AP, Lawrie H, Kennedy A, Webb A, Torgerson D, Grant A. Cost effectiveness of a prize draw on response to a postal questionnaire:results of a randomised trial among orthopaedic outpatients in Edinburgh. *Journal of Epidemiology and Public Health* 1997; 51:463-464.

(31) Man MS, Tilbrook HE, Jayakody S, Hewitt CE, Cox H, Cross B et al. Electronic reminders did not improve postal questionnaire response rates or response times: a randomized controlled trial. *J Clin Epidemiol* 2011; 64(9):1001-1004.

(32) McCambridge J, Kalaitzaki E, White RI, Khadjesari Z, Murray E, Linke S et al. Impact of Length or Relevance of Questionnaires on Attrition in Online Trials: Randomized Controlled Trial. *J Med Internet Res* 2011; 13(4):e96.

(33) McColl EM, Eccles MPM, Rousseau NSB, Steen INP, Parkin DWD, Grimshaw JMP. From the Generic to the Condition-specific?: Instrument Order Effects in Quality of Life Assessment. [Article]. *Med Care* 2003; 41(7):777-790.

(34) Nakash R. A study of response and non-response to postal questionnaire follow-up in clinical trials. Chapter 6: A radomised controlled trial of a method of improving response to postal questionnaire follow-up in a clinical trial. [University of Warwick; 2007.

(35) Severi E, Free C, Knight R, Robertson S, Edwards P, Hoile E. Two controlled trials to increase participant retention in a randomized controlled trial of mobile phone-based smoking cessation support in the United Kingdom. *Clinical Trials* 2011; 8(5):654-660.

(36) Subar AF, Ziegler RG, Thompson FE, Johnson CC, Weissfeld JL, Reding D et al. Is Shorter Always Better? Relative Importance of Questionnaire Length and Cognitive Ease on Response Rates and Data Quality for Two Dietary Questionnaires. *Am J Epidemiol* 2001; 153(4):404-409.

(37) Sutherland HJ, Beaton M, Mazer R, Kriukov V, Boyd NF. A randomized trial of the total design method for the postal follow-up of women in a cancer prevention trial. *Eur J Cancer Prev* 1996; 5(3):165-168.

(38) Tai SS, Nazareth I, Haines A, Jowett C. A randomized trial of the impact of telephone and recorded delivery reminders on the response rate to research questionnaires. *J Public Health* 1997; 19(2):219-221.

For peer review only - http://bmjopen.bmj.com/site/about/guidelines.xhtml

BMJ Open REVIEW submitted 07.08.2013

(39) Kenyon S, Pike K, Jones D, Taylor D, Salt A, Marlow N et al. The effect of a monetary incentive on return of a postal health and development questionnaire: a randomised trial [ISRCTN53994660]. *BMC Health Services Research* 2005; 5(1):55.

(40) JR Hughes. Free reprints to increase the return of follow-up questionnaires. *<[11] Journal>* 1989.

(41) The International Stroke Trial (IST): a randomised trial of aspirin, subcutaneous heparin, both, or neither among 19[punctuation space]435 patients with acute ischaemic stroke. *The Lancet* 1997; 349(9065):1569-1581.

(42) Ford ME, Havstad S, Vernon SW, Davis SD, Kroll D, Lamerato L et al. Enhancing Adherence Among Older African American Men Enrolled in a Longitudinal Cancer Screening Trial. *Gerontologist* 2006; 46(4):545-550.

(43) Marson A, Appleton R, BakerG, Chadwick D, Doughty J, Eaton B et al. A randomised controlled trial examining the longer-term outcomes of standard versus new antiepileptic drugs The SANAD trial. *NIHR HTA Report* 2007; 11(37).

(44) Nakash R, Hutton J, Jorstad-Stein E, Gates S, Lamb S. Maximising response to postal questionnaires – A systematic review of randomised trials in health research. *BMC Medical Research Methodology* 2006; 6(1):5.

Reference list of host trials within which retention trials were embedded [23;26;41;46-701-28].

Formatted: Do not check spelling or grammar, Superscript

Reference List

(1) Boyd N, Cousins M, Lockwood G, Tritchler D. Dietary fat and breast cancer risk: The feasibility of a clinical trial of breast cancer prevention. *Lipids* 1992; 27(10):821-826.

(2) Buys SS, Partridge E, Greene MH, Prorok PC, Reding D, Riley TL et al. Ovarian cancer screening in the Prostate, Lung, Colorectal and Ovarian (PLCO) cancer screening trial: Findings from the initial screen of a randomized trial. *Am J Obstet Gynecol* 2005; 193(5):1630-1639.

For peer review only - http://bmjopen.bmj.com/site/about/guidelines.xhtml

BMJ Open REVIEW submitted 07.08.2013

~~(3) Chaffin M, Valle LA, Funderburk B, Gurwitch R, Silovsky J, Bard D et al. A Motivational Intervention Can Improve Retention in PCIT for Low-Motivation Child Welfare Clients. *Child Maltreatment* 2009; 14(4):356-368.~~

~~(4) Cox KL, Burke V, Beilin LJ, Derbyshire AJ, Grove JR, Blanksby BA et al. Short and long-term adherence to swimming and walking programs in older women -- The Sedentary Women Exercise Adherence Trial (SWEAT 2). *Prev Med* 2008; 46(6):511-517.~~

~~(5) Cooke MW, Marsh JL, Clarke M, Nakash R, Jarvis RM, Hutton JL et al. Treatment of severe ankle sprain: a pragmatic randomised controlled trial comparing the clinical effectiveness and cost effectiveness of three types of mechanical ankle support with tubular bandage. The CAST trial. 13, 1-144. 2009. NIHR Health Technology Assessment Programme.~~
~~Ref Type: Report~~

~~(6) Effect of intravenous corticosteroids on death within 14 days in 10[punctuation space]008 adults with clinically significant head injury (MRC CRASH trial): randomised placebo-controlled trial. *The Lancet* 2004; 364(9442):1321-1328.~~

~~(7) Dennis CL, Hodnett E, Kenton L, Weston J, Zupancic J, Stewart DE et al. Effect of peer support on prevention of postnatal depression among high risk women: multisite randomised controlled trial. *BMJ* 2009; 338(jan15_2):a3064.~~

~~(8) Eccles M, McColl E, Steen N, Rousseau N, Grimshaw J, Parkin D et al. Effect of computerised evidence based guidelines on management of asthma and angina in adults in primary care: cluster randomised controlled trial. *BMJ* 2002; 325(7370):941.~~

~~(9) Free C, Knight R, Robertson S, Whittaker R, Edwards P, Zhou W et al. Smoking cessation support delivered via mobile phone text messaging (txt2stop): a single-blind, randomised trial. *The Lancet* 2011; 378(9785):49-55.~~

~~(10) Gail MH, Byar DP, Pechacek TF, Corle DK. Aspects of statistical design for the community intervention trial for smoking cessation (COMMIT). *Control Clin Trials* 1992; 13(1):6-21.~~

~~(11) Hughes JR, Hatsukami D, Pickens R, Krahn D, Malin S, Luknic A. Effect of nicotine on the tobacco withdrawal syndrome. *Psychopharmacology (Berl)* 1984; 83(1):82-87.~~

~~(12) The International Stroke Trial (IST): a randomised trial of aspirin, subcutaneous heparin, both, or neither among 19[punctuation space]435 patients with acute ischaemic stroke. *The Lancet* 1997; 349(9065):1569-1581.~~

~~(13) Kenyon SL, Taylor DJ, Tarnow-Mordi W. Broad-spectrum antibiotics for preterm, prelabour rupture of fetal membranes: the ORACLE I randomised trial. *The Lancet* 2001; 357(9261):979-988.~~

~~(14) Kenyon SL, Taylor DJ, Tarnow-Mordi W. Broad-spectrum antibiotics for spontaneous preterm labour: the ORACLE II randomised trial. *The Lancet* 2001; 357(9261):989-994.~~

~~(15) Leigh Brown A, Kennedy A, Torgerson D, Campbell J, Webb J, Grant A. The OMENS trial: opportunistic evaluation of musculo-skeletal physician care among orthopaedic outpatients unlikely to require surgery. *Health Bull (Edinb)* 2001; 59(3):198-210.~~

~~(16) Marson AG, Al Kharusi AM, Alwaidh M, Appleton R, Baker GA, Chadwick DW et al. The SANAD study of effectiveness of valproate, lamotrigine, or topiramate for generalised and~~

For peer review only - http://bmjopen.bmj.com/site/about/guidelines.xhtml

BMJ Open REVIEW submitted 07.08.2013

unclassifiable epilepsy: an unblinded randomised controlled trial. *The Lancet* 2007; 369(9566):1016-1026.

(17) Lamb S, Gates S, Underwood M, Cooke M, Ashby D, Szczepura A et al. Managing Injuries of the Neck Trial (MINT): design of a randomised controlled trial of treatments for whiplash associated disorders. *BMC Musculoskeletal Disorders* 2007; 8(1):7.

(18) Murray E, McCambridge J, Khadjesari Z, White I, Thompson S, Godfrey C et al. The DYD-RCT protocol: an on-line randomised controlled trial of an interactive computer-based intervention compared with a standard information website to reduce alcohol consumption among hazardous drinkers. *BMC Public Health* 2007; 7(1):306.

(19) Omenn GS, Goodman GE, Thornquist MD, Balmes J, Cullen MR, Glass A et al. Effects of a Combination of Beta Carotene and Vitamin A on Lung Cancer and Cardiovascular Disease. *N Engl J Med* 1996; 334(18):1150-1155.

(20) Porthouse J, Sarah C, Christine K, Lucy S, Elizabeth S, Terry A et al. Randomised controlled trial of calcium and supplementation with cholecalciferol (vitamin D3) for prevention of fractures in primary care. *BMJ* 2005; 330.

(21) Oral vitamin D3 and calcium for secondary prevention of low-trauma fractures in elderly people (Randomised Evaluation of Calcium Or vitamin D, RECORD): a randomised placebo-controlled trial. *The Lancet* 2007; 365(9471):1621-1628.

(22) Tai S, Nazareth I, Donegan C, Haines A. Evaluation of General Practice Computer Templates. *Methods Inf Med* 1999; 38:177-181.

(23) The Antiarrhythmics versus Implantable Defibrillators (AVID) Investigators. A Comparison of Antiarrhythmic-Drug Therapy with Implantable Defibrillators in Patients Resuscitated from Near-Fatal Ventricular Arrhythmias. *N Engl J Med* 1997; 337(22):1576-1584.

(24) Tilbrook HE, Cox H, Hewitt CE, Kang'ombe AR, Chuang LH, Jayakody S et al. Yoga for Chronic Low Back PainA Randomized Trial. *Ann Intern Med* 2011; 155(9):569-578.

(25) Rothert K, Strecher VJ, Doyle LA, Caplan WM, Joyce JS, Jimison HB et al. Web-based Weight Management Programs in an Integrated Health Care Setting: A Randomized, Controlled Trial[ast]. *Obesity* 2006; 14(2):266-272.

(26) TOMBOLA Group. Cytological surveillance compared with immediate referral for colposcopy in management of women with low grade cervical abnormalities: multicentre randomised controlled trial. *BMJ* 2009; 339(jul28_2):b2546.

(27) TOMBOLA Group. Biopsy and selective recall compared with immediate large loop excision in management of women with low grade abnormal cervical cytology referred for colposcopy: multicentre randomised controlled trial. *BMJ* 2009; 339(jul28_2):b2548.

(28) UK BEAM Trial Team. United Kingdom back pain exercise and manipulation (UK BEAM) randomised trial: effectiveness of physical treatments for back pain in primary care. *BMJ* 2004;bmj.

For peer review only - http://bmjopen.bmj.com/site/about/guidelines.xhtml

For peer review only

Strategies to improve retention in randomised trials: a systematic review and meta-analysis: tables

Table 1 Characteristics of included incentive trials

Trial	Number randomised	Disease/Condition	Participant in main trials	Setting	Intervention(s)	Control	Outcome attrition trial	Time point used in analysis
**Addition of monetary incentive vs none**								
Bauer 2004 (ab)	300	Treatment smoking dependence	Smokers (Gail 1992)	USA Community	*a)* $10 cheque *b)* $2 cheque Arms combined	No cheque	DNA specimen kit return plus postal questionnaire response	Overall number of kits returned
Gates 2009	2144	Treatment neck injury	Patients with whiplash injury (Lamb 2007)	UK hospital trusts	£5 voucher	No voucher	Postal questionnaire response at 2 weeks	2 week response
Kenyon 2005	722	Treatment preterm labour	Women 7 years post participation in ORACLE trial (Kenyon 2001)	UK secondary care/community	£5 voucher	No voucher	Postal questionnaire response	Overall response
**Addition of offer of monetary incentive/prize draw vs none**								
Khadjesari 2011 (1ac)	1022	Treatment alcohol dependence	Adults scoring +5 on Audit C (Murray 2007)	UK Community: Web based	*a)* Offer £5 voucher *c)* Offer entry £250 prize draw Arms combined	No offer	Web based questionnaire response	Response within 40 days of first reminder
Khadjesari 2011 (2)	2591	Treatment alcohol dependence	Adults scoring +5 on Audit C (Murray 2007)	Community: Web based	Offer £10 Amazon voucher	No offer	Web based questionnaire response	Response within 40 days of first reminder
**Addition of non-monetary incentive vs none**								

For peer review only

Trial	Number randomised	Disease/Condition	Participant in main trials	Setting	Intervention(s)	Control	Outcome attrition trial	Time point used in analysis
Bowen 2000 (abc)	4728	Prevention lung cancer	Adults exposed to smoking and asbestos (Omenn 1996)	USA sites	a) Certificate<br>b) Pin<br><br>c) Pin and certificate<br><br>Arms combined	No certificate /pin	Trial retention	Time from randomisation to first inactivation (stop taking vitamins or placebo) during PRIDE 2 year follow-up
Renfroe 2002 (a)	664	Treatment ventricular fibrillation ventricular tachycardia	Adultscardioverted from VT or resuscitated from VF (AVID 1997)	USA hospital	Certificate of appreciation	No certificate	Postal questionnaire response	Overall response
Sharp 2006 (a)	231	Screening cervical cancer	Women with low grade abnormal cervical smear (TOMBOLA group 2009)	UK primary care	Pen	No pen	Postal questionnaire response	Overall response
Sharp 2006 (b)	232	Screening cervical cancer	Women with low grade abnormal cervical smear (TOMBOLA Group 2009)	UK primary care	Pen	No pen	Postal questionnaire response	Overall response
Sharp 2006 (c)	233	Screening cervical cancer	Women with low grade abnormal cervical smear (TOMBOLA	UK primary care	Pen	No pen	Postal questionnaire response	Overall response

For peer review only

For peer review only - http://bmjopen.bmj.com/site/about/guidelines.xhtml

Trial	Number randomised	Disease/Condition	Participant in main trials	Setting	Intervention(s)	Control	Outcome attrition trial	Time point used in analysis
			Group 2009)					
Sharp 2006 (d)	234	Screening cervical cancer	Women with low grade abnormal cervical smear (TOMBOLA Group 2009)	UK primary care	Pen	No pen	Postal questionnaire response	Overall response
**Addition of offer of non-monetary incentive vs no offer**								
Cockayne 2005	1038	Prevention fracture	Women with hip fracture risk factors micro nutrient trial (Porthouse 2005)	UK primary care	Offer of study results	No offer	Postal questionnaire response	Overall response
Hughes 1989	100	Treatment smoking dependence	Adult smokers (Hughes 1984)	USA community	Offer results reprint	No offer	Postal questionnaire response	Overall response
**Addition of offer of monetary donation to charity vs no offer**								
Khadjesari 2011 (1b)	815	Treatment alcohol dependence	Adults scoring +5 on Audit C (Murray 2007)	Community: on line	Offer £5 charity donation	No offer	Web based questionnaire response	Response within 40 days of first reminder
**Addition of £10 plus offer of £10 vs addition of £5 plus offer of £5**								
Bailey (unpublished)	417	Promotion sexual health	Young people (feasibility study sex un zipped	Community UK on line	Offer of £20 shopping voucher	Offer of £10 shopping	Postal questionnaire response	Response at 3 month follow-up

For peer review only - http://bmjopen.bmj.com/site/about/guidelines.xhtml

Trial	Number randomised	Disease/Condition	Participant in main trials	Setting	Intervention(s)	Control	Outcome attrition trial	Time point used in analysis
			trial)			voucher		
**Addition of £20 voucher offer vs addition of £10 voucher offer**								
Bailey (unpublished)	485	Promotion sexual health	Young (feasibility study sex un zipped trial)	Community UK on line	£10 shopping voucher + offer of £10 shopping voucher	£5 shopping voucher + offer of £5 shopping voucher	Postal questionnaire response and chlamydia kit return	Response at 3 month follow-up
**Addition of monetary incentive vs offer of entry into prize draw**								
Kenton 2007 (a)	147	Prevention post natal depression	Women postpartum at high risk of postnatal depression (Dennis 2009)	Canada community	$2 coin	Draw for $50 gift voucher	Postal questionnaire response	Overall Response
Kenton 2007 (b)	150	Prevention post natal depression	Women postpartum at high risk of postnatal depression (Dennis 2009)	Canada community	$2 coin	Draw for $50 gift voucher	Postal questionnaire response	Overall response
**Offer of prize draw entry vs no offer**								
Leighbrown 1997	1307	Clinical management orthopaedics	Adults non-surgical musculoskeletal conditions (Leigh Brown 2001)	UK Hosp out patients department	Aware Offer of monthly prize draw of £25 gift voucher	No offer	Postal questionnaire response after first and 2nd reminder	No data available

For peer review only - http://bmjopen.bmj.com/site/about/guidelines.xhtml

Table 2 Characteristics of included communication trials

Trial	Number randomised	Main/ Attrition trial area	Participants	Setting	Intervention	Control	Outcome attrition trial	Time point used in analysis
**Enhanced letter vs standard letter**								
Renfroe 2002 (c)	664	Treatment ventricular fibrillation ventricular tachycardia	Adults cardioverted from VT or resuscitated from VF (AVID Investigators 1997)	USA hospital	Cover letter signed by physician	Cover letter signed by coordinator	Postal questionnaire response	Overall response
Marson 2007	1815	Treatment epilepsy	Adults with epilepsy mean SANAD trial. (Marson 2007)	UK hospital outpatient departments	Letter explaining the approximate time needed to complete questionnaire	Standard letter	Postal questionnaire response	Overall response
**Total design postal method for postal questionnaires vs customary method**								
Sutherland 1 996	226	Prevention breast cancer	Women with 50% breast volume dysplasia (Boyd 1992)	Canada Hosp clinic	Total design method for postal follow-up	Customary method for postal follow-up	Postal questionnaire response	Response at day 70.
**Priority vs regular post**								
Renfroe 2002 (b)	664	Treatment ventricular fibrillation ventricular tachycardia	Adults cardioverted from VT or resuscitated from VF (AVID) Investigators 1997)	USA hospital	Overnight questionnaire delivery	Standard questionnaire delivery	Postal questionnaire response	Overall response No of questionnaires returned

For peer review only - http://bmjopen.bmj.com/site/about/guidelines.xhtml

Trial	Number randomised	Main/ Attrition trial area	Participants	Setting	Intervention	Control	Outcome attrition trial	Time point used in analysis
Sharp 2006 (*e*)	233	Screening cervical cancer	Women with low grade abnormal cervical smear (TOMBOLA Group 2009)	UK primary care	1st class outward post	2nd class outward post	Postal questionnaire response	Overall response
Sharp 2006 (*f*)	231	Screening cervical cancer	Women with low grade abnormal cervical smear (TOMBOLA Group 2009)	UK primary care	1st class outward post	2nd class outward post	Postal questionnaire response	Overall response
Sharp 2006 (*g*)	240	Screening cervical cancer	Women with low grade abnormal cervical smear (TOMBOLA Group 2009)	UK primary care	Stamped reply envelope	Business reply envelope	Postal questionnaire response	Overall response
Sharp 2006 (*h*)	223	Screening cervical cancer	Women with low grade abnormal cervical smear (TOMBOLA Group 2009)	UK primary care	Stamped reply envelope	Business reply envelope	Postal questionnaire response	Overall response
Kenton 2007 (*c*)	149	Screening post natal depression	Women postpartum at high risk of postnatal depression (Dennis 2009)	Canada community	Priority outward mail	Regular outward mail	Postal questionnaire response	Overall response
Kenton 2007	148	Screening	Women postpartum at	Canada	Priority	Regular outward mail	Postal	Overall

For peer review only

Trial	Number randomised	Main/ Attrition trial area	Participants	Setting	Intervention	Control	Outcome attrition trial	Time point used in analysis
(*d*)		post natal depression	high risk of postnatal depression (Dennis 2009)	community	outward mail		questionnaire response	response
**Additional reminder vs usual follow-up procedures**								
Ashby 2011	148	Prevention migraine	Adults history of two migraine attacks	UK community	Electronic reminder (email and /or SMS text)	No electronic reminder	Postal questionnaire response	Response at 40 days
Maclennan unpublished	753	Prevention fracture	Adults with history of osteoporotic fracture (RECORD Trial Group 2005)	UK hospital	Telephone reminder (before receiving first reminder)	No telephone reminder	Postal questionnaire response	Overall response Response rate
Nakash unpublished	298	Treatment of ankle injury	Cast trial: Adults with acute severe ankle sprain (Cooke 2009)	UK Accident and emergency departments	Trial calendar with questionnaire. due dates	No calendar	Postal questionnaire response at 4, 12 weeks, and 9 months.	Response at 4 weeks
Severi 2011 (1)	1950	Treatment smoking dependence	Adult smokers willing to quit in Txt2stop (Free 2011)	UK community	Text message and fridge magnet emphasising social benefits of study	Text message 3 days after questionnaire sent reminding questionnaire is due	Postal questionnaire response	Response at 30 weeks from randomisation.

For peer review only - http://bmjopen.bmj.com/site/about/guidelines.xhtml

Trial	Number randomised	Main/ Attrition trial area	Participants	Setting	Intervention	Control	Outcome attrition trial	Time point used in analysis
					participation.			
Severi 2011 (2)	127	Treatment smoking dependence	Adult smokers willing to quit in Txt2stop (Free 2011)	UK community	Telephone reminder from principle investigator that participants six weeks overdue returning their specimens	Standard text and no phone call from principle investigator	Return of cotinine samples	Completed cotinine sample follow-up for Txt2stop at end of May 2009
Man 2011	125	Treatment back pain	Adults with back pain (Tilbrook 2011)	UK primary care	SMS text reminder message as follow-up questionnaire sent out	No SMS text message	Postal questionnaire response	Overall Response rate
**Monthly reminder of upcoming assessment to site vs usual reminders**								
Land 2007	429	Treatment breast cancer	Women with ductal carcinoma in situ (unpublished)	Hospital sites USA, Canada, Puerto Rico	Prospective monthly reminder of upcoming assessments to sites	No extra reminders to sites	Postal questionnaire response	Overall Response rate
**Early vs late administartion of questionnaire**								
Renfroe 2002 (d)	664	Treatment ventricular fibrillation ventricular tachycardia	Adults cardioverted from VT or resuscitated from VF (AVID) Investigators 1997)	USA hospital	Questionnaire sent 2-3 weeks after last AVID follow-up visit	Questionnaire sent 1-4 months after last AVID follow-up visit	Postal questionnaire response	Overall response Number of questionnaires returned
**Recorded delivery vs telephone reminder**								

For peer review only - http://bmjopen.bmj.com/site/about/guidelines.xhtml

Trial	Number randomised	Main/ Attrition trial area	Participants	Setting	Intervention	Control	Outcome attrition trial	Time point used in analysis
Tai 1997	192	Clinical management asthma and diabetes	Adults with asthma or diabetes (Tai 1999)	UK primary care	Recorded delivery reminder	Telephone reminder	Postal questionnaire response	Overall response Number of questionnaires returned used
**Telephone interview vs questionnaire and monetary incentive**								
Couper 2007	700	Weight management	Adults with BMI >25 (Rothert 2006	USA community web based	Telephone interview by trained interviewer	Postal questionnaires with $5 bill	Post and telephone questionnaire response	Response at 6 months

For peer review only

Table 3 Characteristics of included trials evaluating new questionnaire strategies

Trial	Number of participants	Main/ attrition trial area	Participants	Setting	Intervention	Control	Outcome attrition trial	Time point used in analysis
**Short versus long questionnaire**								
Dorman 1997	2253	Treatment Stroke	Stroke patients (International Stroke Trial 1997)	UK hospital	Short EUROQOL questionnaire	Long SF 36 questionnaire	Postal questionnaire response after first mail out and reminder	Response at first time point.
Edwards 2001 unpublished	99	Treatment head injury	Head injury patients (CRASH Trial 2004)	UK hospital intensive care units	1-page, 7 question functional dependence questionnaire	3-page, 16 question functional dependence questionnaire.	Postal questionnaire response	Response at 3 months
Svoboda 2001 unpublished	91	Treatment head injury	Head injury patients (CRASH Trial 2004)	Czech republic hospital intensive care units	1-page, 7 question functional dependence questionnaire	3-page, 16 question functional dependence questionnaire.	Postal questionnaire response	Response at 3 months
Mc Cambridge 2011 1b	2835	Treatment alcohol dependence	Adults scoring +5 on Audit C (Murray 2007)	Community web based	Audit Short (alcohol use disorders questionnaire) + LDQ (Leeds dependancy	APQ (alcohol problems questionnaire)	Web based questionnaire response at 1 month and 3 months	Response at 1 month

For peer review only - http://bmjopen.bmj.com/site/about/guidelines.xhtml

Trial	Number of participants	Main/ attrition trial area	Participants	Setting	Intervention	Control	Outcome attrition trial	Time point used in analysis
					questionnaire)			
Mc Cambridge 2011 2b	1999	Treatment Alcohol dependence	Adults scoring +5 on Adults scoring +5 on Audit C (Murray 2007)	Community web based	Audit Short (alcohol use disorders questionnaire)<br>+<br>LDQ (Leeds dependancy questionnaire)	APQ (alcohol problems questionnaire)	Web based questionnaire response at 3 month and 12 months	Response at 3 months
**Long and clear versus short and condensed questionnaires**								
Subar 2001	900	Screening prostate, lung, ovarian, colorectal cancer	Adults in PLCO trial (Prorok 2000)	USA sites	DHQ (36-page food frequency questionnaire)	PLCO (16-page food frequency questionnaire)	Postal questionnaire/ response on site completion	Overall response
**Question order: condition first vs generic first questions**								
Mc Coll 2003 (1)	4751	Clinical management asthma	Adult with asthma in COGENT Trial: (Eccles 2002)	UK primary care	Condition specific questions first followed by generic	Generic questions followed by condition specific	Postal questionnaireresponse	Overall response
Mc Coll 2003 (2)	4684	Clinical management angina	Adult with angina in the COGENT Trial: (Eccles 2002)	UK primary care	Condition specific questions followed by generic	Generic questions followed by condition specific	Postal questionnaire response	Overall response

For peer review only - http://bmjopen.bmj.com/site/about/guidelines.xhtml

Trial	Number of participants	Main/ attrition trial area	Participants	Setting	Intervention	Control	Outcome attrition trial	Time point used in analysis
Letley unpublished. No data available	Data not available	Treatment back pain	Adults with low back pain (UK BEAM trial team 2004)	UK primary care	23 page self-completion questionnaire Roland disability questionnaire at front and SF 36 at back	vice versa	Questionnaire response	No data
**Questionnaire: relevant versus less relevant to condition**								
Mc Cambridge 2011 1a	1892	Treatment alcohol dependence	Adults scoring +5 on Audit C (Murray 2007)	Community web based	Alcohol problem questionnaire (APQ)23 items	Core OM Mental health assessment 23/34 items	Web based questionnaire response at 1 and 3 months	Response at 1 month
Mc Cambridge 2011 2a	2001	Treatment alcohol dependence	Adults scoring +5 on Audit C (Murray 2007)	Community web based	Audit Short (alcohol use disorders questionnaire) + LDQ (Leeds dependancy questionnaire)	Core OM Mental health assessment 10 items	Web based questionnaire response at 3 month and 12 months	Response at 3 months

For peer review only - http://bmjopen.bmj.com/site/about/guidelines.xhtml

Table 4 Characteristics of other trials

Trial	Number of participants	Main/ attrition trial area	Participants	Country	Behavioural strategy	Control arms	Outcome attrition trial	Time point used in analysis
**Motivation vs information**								
Cox 2008	120	Exercise improvement	Sedentary Women in SWEAT 2 Trial (Cox 2008)	Australia Community	Motivational workshops and newsletters	Information sheets and newsletters	Program and trial retention at 6 and 12 months	6 month and 12 month data. Data for 6 months used
Chaffin 2009	153	Parenting improvement	Adults referred for parenting improvement (Chaffin 2009)	USA community	Self-motivation information	Standard information	Program attendance/ trial retention	Retention at 12 weeks
**Case management vs usual follow-up**								
Ford 2006	703	Screening prostate, lung, ovarian, colorectal cancer	Adults in the PLCO screening trial (Prorok 2000)	USA sites	In-depth case management	Regular trial procedures	Attendance at face to face cancer screening	Retention at 3 years
**Open vs blind trial design**								
Avenell 2004	538	Prevention fracture	Adults with history of osteoporotic fracture in the RECORD micronutrient trial (RECORD Trial Group 2005)	UK hospital	Open trial design	Blind trial design	Postal questionnaire response at 4, 8,12 months	Response at12 months

For peer review only - http://bmjopen.bmj.com/site/about/guidelines.xhtml

Table 5 Absolute benefit of effective strategies to improve retention

**Example of proportion of questionnaires returned in control arm**			**30%**	**40%**	**50%**	**60%**	**70%**	**80%**	**90%**
Strategy to improve retention	RR	1/ RR							
Addition of monetary incentive versus no incentive	1.18	0.847	107	92	76	61	5	3	2
Addition of offer of monetary incentive/prize draw versus no offer	1.25	0.800	140	120	100	80	60	40	20
Addition of higher value monetary incentive versus addition of lower amount	1.12	0.890	77	66	55	44	33	22	11

For peer review only

For peer review only - http://bmjopen.bmj.com/site/about/guidelines.xhtml

Figure 1Incentive strategies: main analysis addition of incentive versus no incentive

Incentives	Incentive	None	RR 95% CI
**Addition of monetary incentive**			
Bauer (ab)	77/200	34/100	1.13 (0.82 to 1.57)
Gates	560 /1070	493/1074	1.14 (1.05 to 1.24)
Kenyon	156 /369	108/353	1.38 (1.13 to 1.68)
**Total**	**793**/1639	**635**/1527	**1.18 (1.09 to 1.28)**
Heterogeneity: X² = 3.13, df = 2, P = 0.21, I² = 36%			
Overall effect: Z = 4.21 (P < 0.0001)			
**Addition of offer of monetary incentive/prize draw**			
Khadjesari (1ac)	120/411	162 611	1.10 (0.90 to1.35)
Khadjesari (2)	476/1296	364 1295	1.31 (1.17 to 1.46)
**Total**	**596**	**526**/1906	**1.25 (1.14 to 1.38)**
Heterogeneity: X² = 2.13, df = 1 (P = 0.14); I² = 53%			
Overall effect: Z = 4.50 (P < 0.00001)			
**Addition of non-monetary incentive**			
Bowen (abc)	3225 /3542	1082/1186	1.00 (0.98 to 1.02)
Renfroe (a)	171/332	203/332	
Sharp (a)	79/115	70/116	1.14 (0.94 to 1.38)
Sharp (b)	85/125	71/107	1.02 (0.86 to 1.23)
Sharp (c)	81/118	63/115	1.25 (1.02 to 1.54)
Sharp (d)	81/118	75/116	1.06 (0.89 to 1.27)
**Total**	**3722**/4350	**1564**/1972	**1.00 (0.98 to 1.02)**
Heterogeneity: X² = 13.06, df = 5 (P = 0.02); I² = 62%			
Overall effect: Z = 0.11 (P = 0.91)			
**Addition of offer of non-monetary incentive**			
Cockayne (1)	721/788	233/250	0.98 (0.94 to 1.02)
Hughes	37/50	35/50	1.06 (0.83 to 1.35)
**Total**	**758**/838	**268**/300	**0.99 (0.95 to 1.03)**
Heterogeneity: X ² = 0.40, df = 1 (P = 0.52); I² = 0%			
Overall effect: Z = 0.53 (P = 0.60)			
**Addition of offer of monetary donation to charity**			
Khadjesari (1b)	55/204	162 611	1.02 (0.78 to 1.32)
**Total**	55/204	162 **611**	**1.02 (0.78 to 1.32)**
Overall effect: Z = 0.13 (P = 0.90)			
Sub group differences: X ² = 35.55, df = 4 (P < 0.00001), I² = 88.7%			

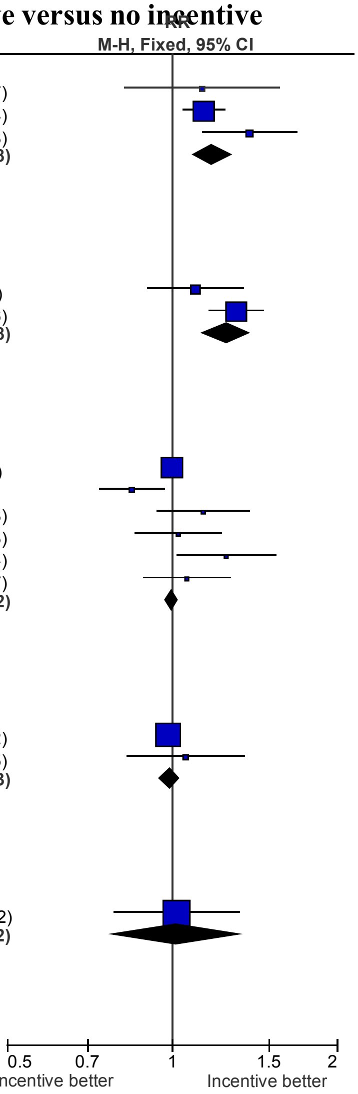

Fig 1b Incentives: addition of £20 vs £10 incentive

Incentive	£20 voucher offer	£10 voucher offer	RR 95% CI
Addition of £10 voucher and £10 offer vs addition of £5 and £5 offer			
Bailey (2)	190 /249	155 /236	1.16 (1.04 to 1.30)
Subtotal	190 /249	155 /236	1.16 (1.04 to 1.30)
Overall effect: Z = 2.55 (P = 0.01)			
Addition of £20 voucher offer vs addition of £10 voucher offer			
Bailey (1)	166 215	144 202	1.08 (0.97 to 1.21)
Subtotal	166 215	144 202	1.08 (0.97 to 1.21)
Overall effect: Z = 1.38 (P = 0.17)			
Total	356 464	299 438	1.12 (1.04 to 1.22)
Heterogeneity: X² = 0.72, df = 1 (P = 0.39); I² = 0%			
Overall effect: Z = 2.82 (P = 0.005)			
Subgroup differences: X² = 0.72, df = 1 (P = 0.40), I² = 0%			

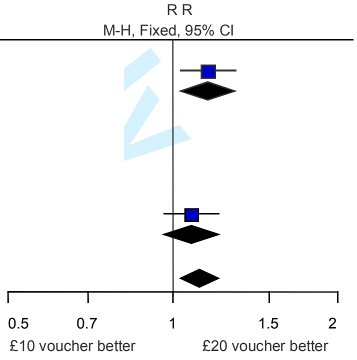

For peer review only

Fig 1c Incentives addition of: monetary incentive vs offer of entry into prize draw

Incentive	Monetary incentive	Entry into draw	RR Fixed, 95% CI
**Addition of monetary incentive vs offer of entry into prize draw**			
Kenton (a)	58 /72	53 /75	1.14 (0.95 to 1.37)
Kenton (b)	55 /77	55 /73	0.95 (0.78 to 1.15)
**Total**	113 /149	108 /148	**1.04 (0.91 to 1.19)**

Heterogeneity: Chi² = 1.83, df = 1 (P = 0.18); I² = 45%
Overall effect: Z = 0.58 (P = 0.56)

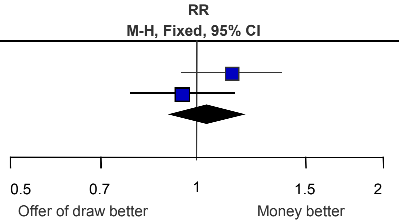

Fig 2a Communication strategies: enhanced vs standard letter

Letters	Enhanced	Standard	RR 95% CI
**Enhanced vs standard**			
Renfroe (c)	180 /332	181 /332	0.99 (0.87 to 1.14)
Marson	756 /891	775 /924	1.01 (0.97 to 1.05)
**Total**	**936 /1223**	**956 /1256**	**1.01 (0.97 to 1.05)**

Heterogeneity: X² = 0.06, df = 1 (P = 0.80); I² = 0%
Overall effect: Z = 0.39 (P = 0.70)

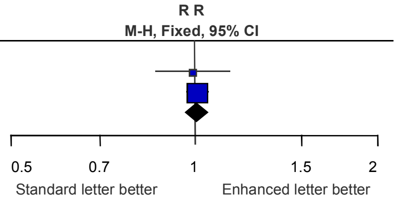

Fig 2b Communication: total design vs customary post

Postal method	Total design	Customary post	RR, 95% CI
**Total design for postal questionnaires vs customary method**			
Sutherland	100 /113	70/113	1.43 (1.22 to 1.67)
**Total**	100 /113	70 /113	**1.43 (1.22 to 1.67)**

Overall effect: Z = 4.39 (P < 0.0001)

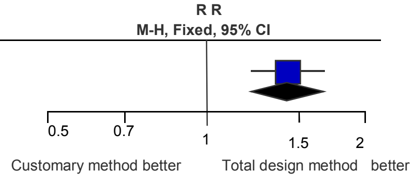

Fig 2c Communication: post priority vs regular post

Post type	Priority	Regular	RR, 95% CI
**Priority vs regular post**			
Renfroe (b)	188 /332	173/332	1.09 (0.95 to 1.25)
Sharp (f)	70 /116	63/115	1.10 (0.88 to 1.38)
Sharp (e)	79 /115	81/118	1.00 (0.84 to 1.19)
Sharp (h)	70 /116	71/107	0.91 (0.74 to 1.11)
Sharp (g)	79 /115	85/125	1.01 (0.85 to 1.20)
Kenton (d)	55 /73	53 /75	1.07 (0.88 to 1.30)
Kenton (c)	55 /77	58 /72	0.89 (0.74 to 1.06)
**Total**	**596 /944**	**584**/944	**1.02 (0.95 to 1.09)**

Heterogeneity: X² = 5.08, df = 6 (P = 0.53); I² = 0%
Overall effect: Z = 0.59 (P = 0.55)

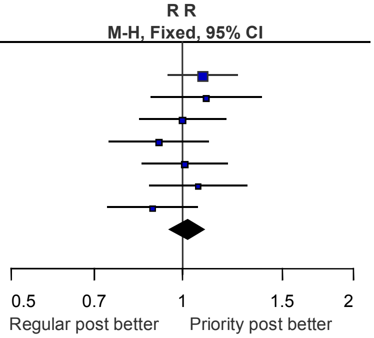

Fig 2d Communication: additional reminders to participants vs usual follow-up

Participant reminder	Additional reminder Response/ No reminders sent		Usual follow-up Response/No reminders sent		RR 95% CI
**Extra reminder vs usual follow-up**					
Ashby	68	74	64	74	1.06 (0.95 to 1.19)
MacLennan	267	390	227	363	1.09 (0.99 to 1.22)
Man	54	62	53	63	1.04 (0.90 to 1.20)
Nakash	117	152	114	146	0.99 (0.87 to 1.11)
Severi (2)	20	65	20	62	0.95 (0.57 to 1.59)
Severi (1)	813	976	801	974	1.01 (0.97 to 1.05)
**Total**	1339	**1719**	1279	**1682**	**1.03 (0.99 to 1.06)**

Heterogeneity: X² = 2.78, df = 5 (P = 0.73); I² = 0%
Overall effect: Z = 1.52 (P = 0.13)

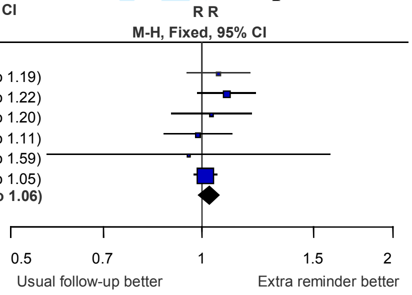

Fig 2e Communication: telephone survey versus monetary incentive and questionnaire

Study or Subgroup	Telephone survey Events	Total	Monetary + questionnaire Events	Total	Risk Ratio M-H, Fixed, 95% CI
**Telephone survey vs monetary incentive and questionnaire**					
Couper 2007	170	300	210	400	1.08 [0.94, 1.24]
**Subtotal (95% CI)**		**300**		**400**	**1.08 [0.94, 1.24]**
Total events	170		210		

Heterogeneity: Not applicable
Test for overall effect: Z = 1.10 (P = 0.27)

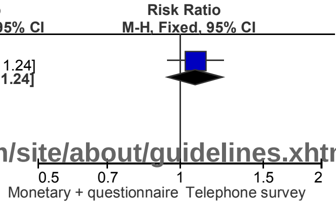

For peer review only - http://bmjopen.bmj.com/site/about/guidelines.xhtml

Fig 3 Questionnaires: new format vs standard format

Questionnaires	New questionnaires Response /No sent	Standard questionnaires Response /No sent	RR 95% CI
**Short vs long**			
Edwards	31/50	35/49	0.87 (0.66 to 1.15)
Dorman	747/1125	679/1128	1.10 (1.04 to 1.17)
Svoboda	29/45	31/46	0.96 (0.71 to 1.29)
Mc Cambridge 2(b)	653/1333	316/666	1.03 (0.94 to 1.14)
Mc Cambridge 1(b)	1049/1888	529/947	0.99 (0.93 to 1.07)
**Total**	**2509/4441**	**1590/2836**	**1.04 (1.00 to 1.08)**
Heterogeneity: X^2 = 6.87, df = 4 (P = 0.14); I^2 = 42%			
Overall effect: Z = 1.82 (P = 0.07)			
**Long and clear vs short and condensed**			
Subar	369/450	367/450	1.01 (0.95 to 1.07)
**Total**	**369/450**	**367/450**	**1.01 (0.95 to 1.07)**
Overall effect: Z = 0.17 (P = 0.86)			
**Question order: condition questions first vs generic first**			
Mc Coll (2)	1779/2363	1738/2321	1.01 (0.97 to 1.04)
Mc Coll (1)	1522/2382	1537/2369	0.98 (0.94 to 1.03)
**Total**	**3301/4745**	**3275/4690**	**1.00 (0.97 to1.02)**
Heterogeneity: X^2 = 0.59, df = 1 (P = 0.44); I^2 = 0%			
Overall effect: Z = 0.31 (P = 0.75)			
**Questionnaire relevant versus less relevant to condition**			
Mc Cambridge 2(a)	653/1333	308/668	1.06 (0.96 to 1.17)
Mc Cambridge 1(a)	529/947	489/945	1.08 (0.99 to 1.17)
**Total**	**1182/2280**	**797/1613**	**1.07 (1.01 to 1.14)**
Heterogeneity: X^2 = 0.06, df = 1 (P = 0.81); I^2 = 0%			
Overall effect: Z = 2.12 (P = 0.03)			
Subgroup differences: X^2 = 6.13, df = 3 (P = 0.11), I^2 = 51.0%			

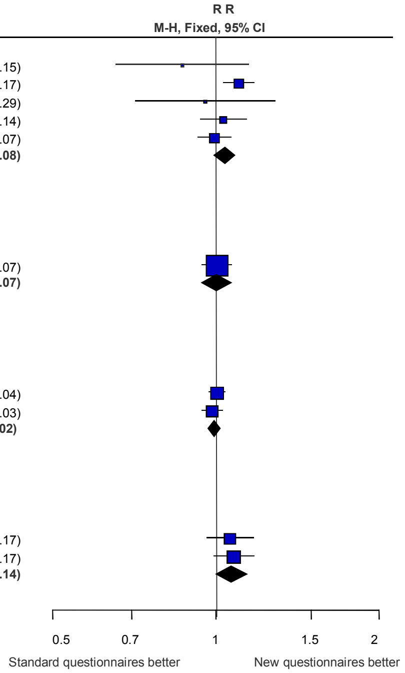

For peer review only

Fig 4 PRISMA diagram

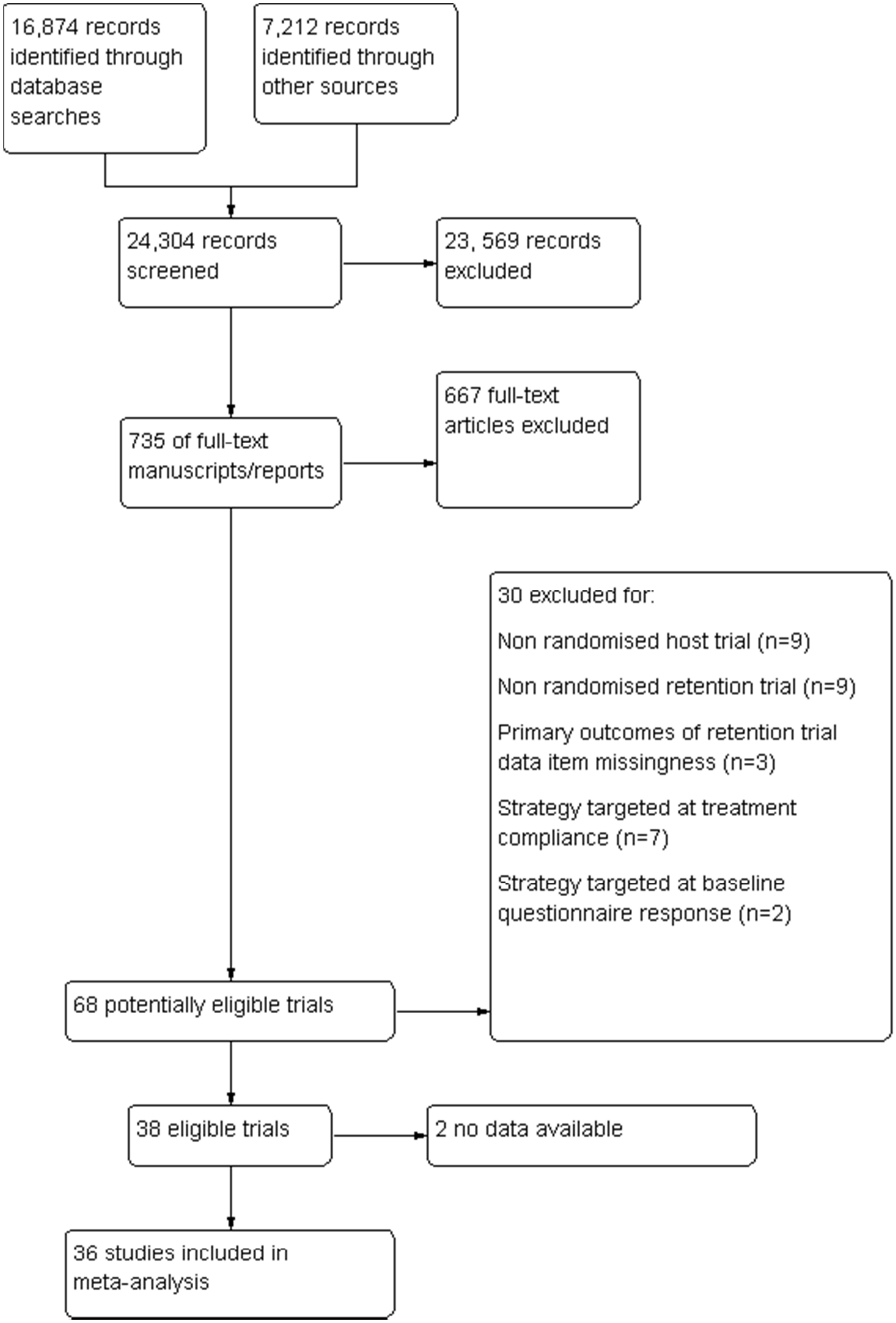

For peer review only - http://bmjopen.bmj.com/site/about/guidelines.xhtml

Figure 5 Exploratory analyses for the main incentives analysis (web appendix)

Study or Subgroup	Incentive Events	Incentive Total	No incentive Events	No incentive Total	Weight	Risk Ratio M-H, Fixed, 95% CI
**3.1.1 Addition of monetary incentive**						
Bauer 2004a	43	100	34	100	5.1%	1.26 [0.89, 1.80]
Bauer 2004b	34	100	34	100	5.1%	1.00 [0.68, 1.47]
Gates 2009	560	1070	493	1074	73.4%	1.14 [1.05, 1.24]
Kenyon 2005	156	369	108	353	16.5%	1.38 [1.13, 1.68]
**Subtotal (95% CI)**		**1639**		**1627**	**100.0%**	**1.18 [1.09, 1.27]**
Total events	793		669			
Heterogeneity: Chi² = 3.91, df = 3 (P = 0.27); I² = 23%						
Test for overall effect: Z = 4.24 (P < 0.0001)						
**3.1.2 Offer of monetary incentive**						
Khadjesari 2011 1a	66	206	162	611	15.5%	1.21 [0.95, 1.53]
Khadjesari 2011 1c	54	205	162	611	15.4%	0.99 [0.76, 1.29]
Khadjesari 2011 2	476	1296	364	1295	69.1%	1.31 [1.17, 1.46]
**Subtotal (95% CI)**		**1707**		**2517**	**100.0%**	**1.24 [1.13, 1.37]**
Total events	596		688			
Heterogeneity: Chi² = 3.57, df = 2 (P = 0.17); I² = 44%						
Test for overall effect: Z = 4.49 (P < 0.00001)						
**3.1.3 Addition of non monetary incentive**						
Bowen 2000a	988	1092	1082	1186	21.7%	0.99 [0.97, 1.02]
Bowen 2000b	1120	1211	1082	1186	22.9%	1.01 [0.99, 1.04]
Bowen 2000c	1117	1239	1082	1186	23.1%	0.99 [0.96, 1.01]
Renfroe 2002a	171	332	203	332	4.2%	0.84 [0.74, 0.96]
Sharp 2006a	79	115	70	116	1.5%	1.14 [0.94, 1.38]
Sharp 2006b	1201	1239	1062	1092	23.6%	1.00 [0.98, 1.01]
Sharp 2006c	81	118	75	116	1.6%	1.06 [0.89, 1.27]
Sharp 2006d	81	118	63	115	1.3%	1.25 [1.02, 1.54]
**Subtotal (95% CI)**		**5464**		**5329**	**100.0%**	**1.00 [0.98, 1.01]**
Total events	4838		4719			
Heterogeneity: Chi² = 15.47, df = 7 (P = 0.03); I² = 55%						
Test for overall effect: Z = 0.38 (P = 0.70)						

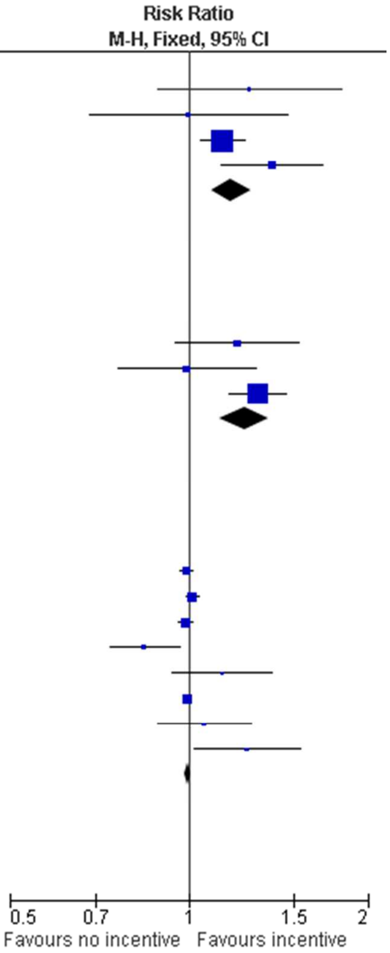

Test for subgroup differences: Chi² = 37.44, df = 2 (P < 0.00001), I² = 94.7%

For peer review only

For peer review only - http://bmjopen.bmj.com/site/about/guidelines.xhtml

MEDLINE search strategy

Search strategy for MEDLINE Cochrane Highly Sensitive Search Strategy for identifying randomized trials in MEDLINE: Sensitivity and precision maximising version, 2008 revision Lefebvre 2008; Ovid format.

randomized controlled trial.pt.

controlled clinical trial.pt.

randomized.ab.

placebo.ab.

clinical trials as topic.sh.

randomly.ab.

trial.ti.

exp animals/ not humans. sh.

(minimi$ adj2 attrition).ab,ti.

(prevent$ adj2 attrition).ab,ti.

(lessen$ adj2 attrition).ab,ti.

(decreas$ adj2 attrition).ab,ti.

(reduc$ adj2 attrition).ab,ti.

(minimi$ adj2 drop-out).ab,ti.

(prevent$ adj2 drop-out).ab,ti.

(lessen$ adj2 drop-out).ab,ti.

(decreas$ adj2 drop-out).ab,ti.

(reduc$ adj2 drop-out).ab,ti.

(minimi$ adj2 drop-out$).ab,ti.

(prevent$ adj2 drop-out$).ab,ti.

(lessen$ adj2 drop-out$).ab,ti.

(decreas$ adj2 drop-out$).ab,ti.

(reduc$ adj2 drop-out$).ab,ti.

(minimi$ adj2 drop$-out).ab,ti.

(prevent$ adj2 drop$-out).ab,ti.

(lessen$ adj2 drop$-out).ab,ti.

(decreas$ adj2 drop$-out).ab,ti.

For peer review only

(reduc$ adj2 drop$-out).ab,ti.

minimi$ adj2 dropout$).ab,ti.

(prevent$ adj2 dropout$).ab,ti.

(lessen$ adj2 dropout$).ab,ti.

(decreas$ adj2 dropout$).ab,ti.

(reduc$ adj2 dropout$).ab,ti

(strateg$ adj2 drop$-out) .ab,ti.

(strateg$ adj2 dropout$).ab,ti.

¬(loss adj2 follow-up).ab,ti.

(lost adj2 follow-up).ab,ti.

(loss adj2 followup).ab,ti.

(lost adj2 followup).ab,ti.

(minimi$ adj2 withdrawal).ab,ti.

(prevent$ adj2 withdrawal).ab,ti.

(lessen$ adj2 withdrawal).ab,ti.

(decreas$ adj2 withdrawal).ab,ti.

(reduc$ adj2 withdrawal).ab,ti.

(minimi$ adj2 withdrawal$).ab,ti.

(prevent$ adj2 withdrawal$).ab,ti.

(lessen$ adj2 withdrawal$).ab,ti.

(decreas$ adj2 withdrawal$).ab,ti.

(reduc$ adj2 withdrawal$).ab,ti.

(strateg$ adj2 attrition).ab,ti.

(strateg$ adj2 drop-out).ab,ti.

(strateg$ adj2 dropout).ab,ti.

(strateg$ adj2 follow-up).ab,ti.

(strateg$ adj2 followup).ab,ti.

(increas$ adj2 retention).ab,ti.

(encourag$ adj2 retention).ab,ti.

(maximi$ adj2 retention).ab,ti.

For peer review only

(promot$ adj2 retention).ab,ti.

(improv$ adj2 retention).ab,ti.

(strateg$ adj2 response$).ab,ti.

(strateg$ adj2 (questionnaire$ adj3 response$)).ab,ti.

(increas$ adj2 (questionnaire$ adj3 response$)).ab,ti.

(encourag$ adj2 (questionnaire$ adj3 response$)).ab,ti.

(maximi$ adj2 (questionnaire$ adj3 response$)).ab,ti.

(promot$ adj2 (questionnaire$ adj3 response$)).ab,ti.

(improv$ adj2 (questionnaire$ adj3 response$)).ab,ti.

(increas$ adj2 response$).ab,ti.

(encourag$ adj2 response$).ab,ti.

(maximi$ adj2 response$).ab,ti.

(promot$ adj2 response$).ab,ti.

(improv$ adj2 response$).ab,ti.

(retention adj2 strateg$).ab,ti.

retention rate$.ab,ti.

(retention adj2 method$).ab,ti.

(retention adj2 technique$).ab,ti.

attrition rate$.ab,ti.

(questionnaire$ adj3 (response$ adj2 method$)).ab,ti.

(questionnaire$ adj3 (response adj2 technique$)).ab,ti.

(questionnaire adj response rate$).ab,ti. (1145)

(difficult$ adj2 (retain$ or retention)).ab,ti.

Participant Dropouts/

The search syntax was adapted for different search interfaces

For peer review only
